# New-generation advanced PROTACs as potential therapeutic agents in cancer therapy

**DOI:** 10.1186/s12943-024-02024-9

**Published:** 2024-05-21

**Authors:** Chao Wang, Yujing Zhang, Wujun Chen, Yudong Wu, Dongming Xing

**Affiliations:** 1 Cancer Institute, The Affiliated Hospital of Qingdao University, Qingdao University, Qingdao, 266071 Shandong China; 2 The Affiliated Cardiovascular Hospital of Qingdao University, Qingdao University, Qingdao, 266071 Shandong China; 3 School of Life Sciences, Tsinghua University, Beijing, 100084 China

**Keywords:** New-generation PROTACs, Prodrug, Nanomedicine, Precise protein degradation, Cancer therapy

## Abstract

Proteolysis-targeting chimeras (PROTACs) technology has garnered significant attention over the last 10 years, representing a burgeoning therapeutic approach with the potential to address pathogenic proteins that have historically posed challenges for traditional small-molecule inhibitors. PROTACs exploit the endogenous E3 ubiquitin ligases to facilitate degradation of the proteins of interest (POIs) through the ubiquitin–proteasome system (UPS) in a cyclic catalytic manner. Despite recent endeavors to advance the utilization of PROTACs in clinical settings, the majority of PROTACs fail to progress beyond the preclinical phase of drug development. There are multiple factors impeding the market entry of PROTACs, with the insufficiently precise degradation of favorable POIs standing out as one of the most formidable obstacles. Recently, there has been exploration of new-generation advanced PROTACs, including small-molecule PROTAC prodrugs, biomacromolecule-PROTAC conjugates, and nano-PROTACs, to improve the in vivo efficacy of PROTACs. These improved PROTACs possess the capability to mitigate undesirable physicochemical characteristics inherent in traditional PROTACs, thereby enhancing their targetability and reducing off-target side effects. The new-generation of advanced PROTACs will mark a pivotal turning point in the realm of targeted protein degradation. In this comprehensive review, we have meticulously summarized the state-of-the-art advancements achieved by these cutting-edge PROTACs, elucidated their underlying design principles, deliberated upon the prevailing challenges encountered, and provided an insightful outlook on future prospects within this burgeoning field.

## Introduction

**Fig. 1:**
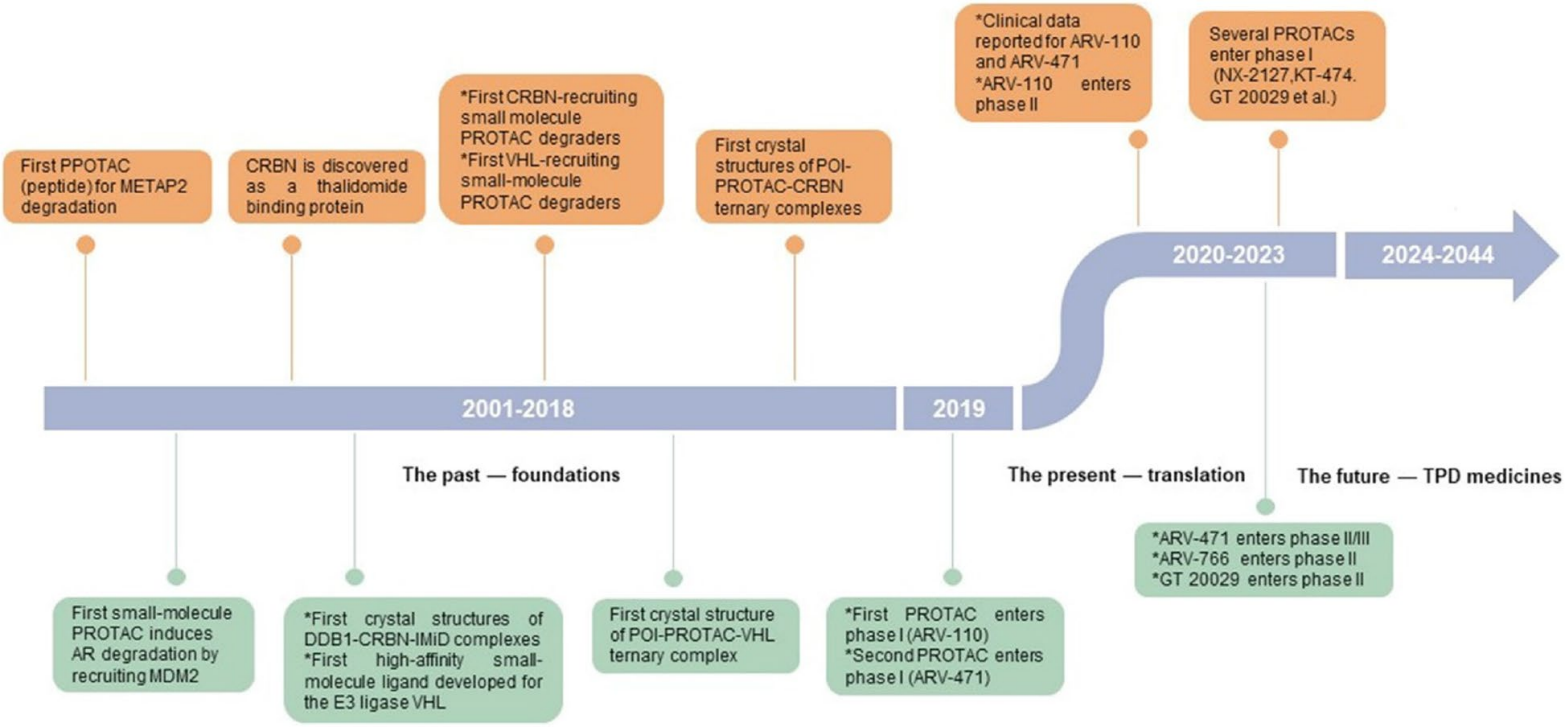
Timeline of PROTAC discoveries (adapted from [3])

**Fig. 2:**
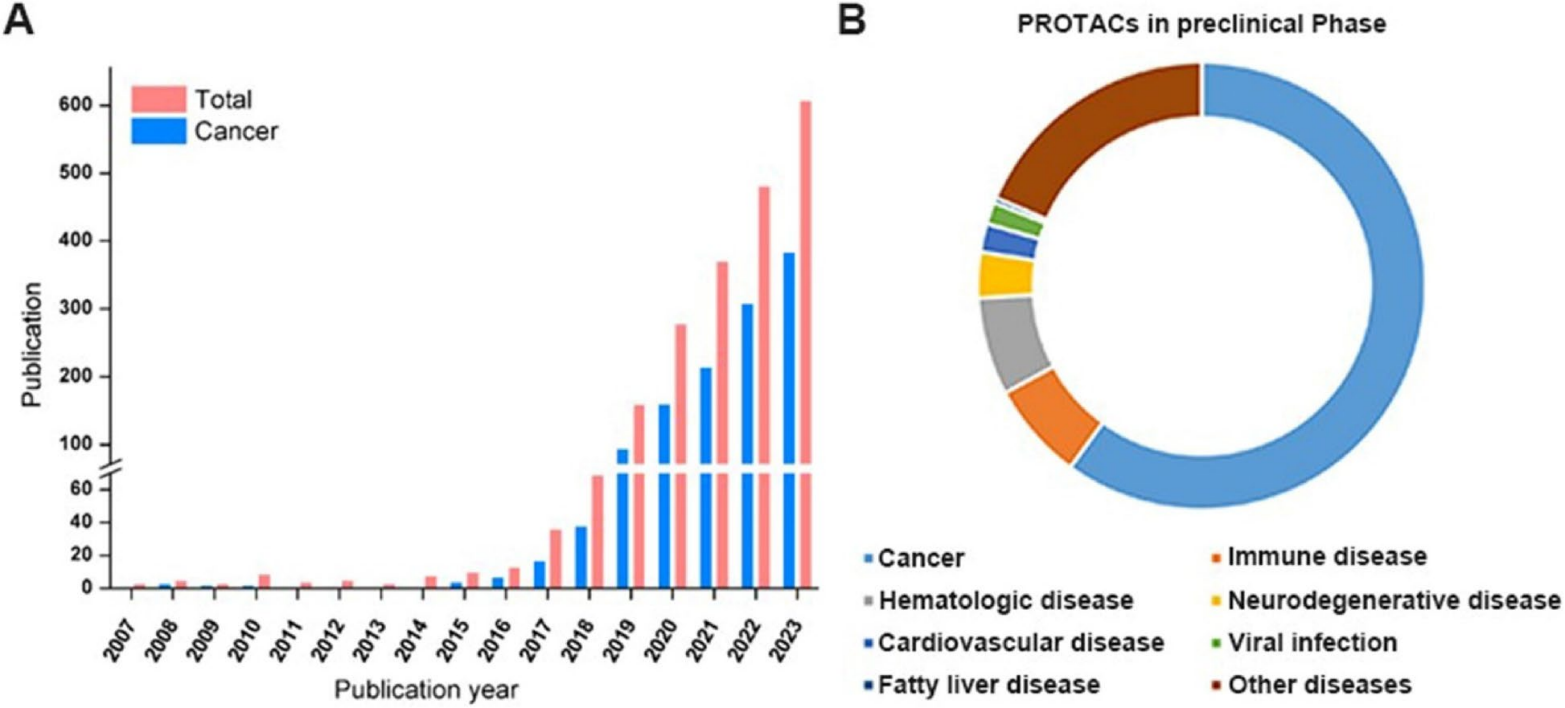
The development history of PROTACs. **A** The total number of PROTAC publications and the part of that about cancer each year (web of science core collection). **B** Percent of PROTACs in different disease fields which are in preclinical phase (web of science core collection)

Proteolysis-targeting chimeras (PROTACs) have emerged as a revolutionary category of therapeutic modalities since their initial documentation in 2001 (Fig. 1) [1–4]. These innovative molecules are meticulously designed to harness the power of the ubiquitin proteasome system (UPS) for targeted protein degradation, offering a promising approach to treating various diseases. Over the years, the field of PROTAC-mediated protein degradation has experienced exponential growth and garnered considerable attention from researchers and pharmaceutical companies alike. This surge in interest is primarily due to the remarkable translational potential demonstrated by these compounds (Fig. 2) [5, 6]. Initially developed as chimeric peptide-based compounds, PROTACs have evolved into cell-permeable small molecules that can efficiently enter cells and selectively degrade disease-causing proteins. The ability of PROTACs to specifically target proteins for degradation holds immense therapeutic promise across multiple areas of medicine. By eliminating disease-associated proteins at their source, these molecules offer a unique advantage over traditional drug therapies that often only inhibit or modulate protein activity. Moreover, PROTACs can potentially address previously "undruggable" targets by exploiting the UPS machinery's natural ability to degrade proteins [7–10]. Following the successful clinical trials of the first two small-molecule degraders against cancer in 2019, numerous other small-molecule PROTACs (Table 1) are now progressing into clinical settings for treating a variety of diseases [2, 11–15]. However, despite the promising preclinical research outcomes, a significant proportion of PROTACs encounter challenges in advancing to human clinical trials [16, 17].

**Table 1:** Summary of PROTAC molecules under and approaching clinical trials (https://clinicaltrials.gov/)

Molecule	Target protein	Indication	Company	Stage
CC-94676	AR	Prostate cancer	Bristol Myers Squibb	Phase I
ARV-766	AR	Prostate cancer	Arvinas	Phase II
ARV-110	AR	Prostate cancer	Arvinas	Phase II
GT20029	AR	Alopecia and acne	Kintor Pharmaceutical	Phase I
ARV-471	ER	Breast cancer	Arvinas	Phase III
AC862	ER	Breast cancer	Accutar Biotech	Phase I
DT2216	BCL-X_L_	Liquid and solid tumors	Dialectic	Phase I
CFT8364	BRD9	Synovial sarcoma, SMARCB1- tumors	C4 Therapeutics	Phase III
FHD-609	BRD9	Synovial sarcoma	Foghorn	Phase I
NX-2127	BTK, Ikaros, Aiolos	B-cell malignancies	Nurix	Phase I
NX-5948	BTK	B-cell malignancies	Nurix	Phase I
BGB-16673	BTK	B-cell malignancies	BeiGene	Phase I
HSK29116	BTK	Relapsed/Refractory B-cell malignancies	Haisco	Phase I
CFT8919	EGFR^L858R^	NSCLC	C4 Therapeutics	Phase I
KT-474	IRAK4	Atopic dermatitis	Kymera	Phase I
KT-413	IRAK4	B-cell NHL	Kymera	Phase I
KT-333	STAT3	Liquid and solid tumors	Kymera	Phase I
CG001419	TRK	Cancer and other diseases	Cullgen	Phase I
LNK-01002	Ras GTPase	Myelofibrosis or myeloid Leukemia	Lynk	Phase I

**Fig. 3:**
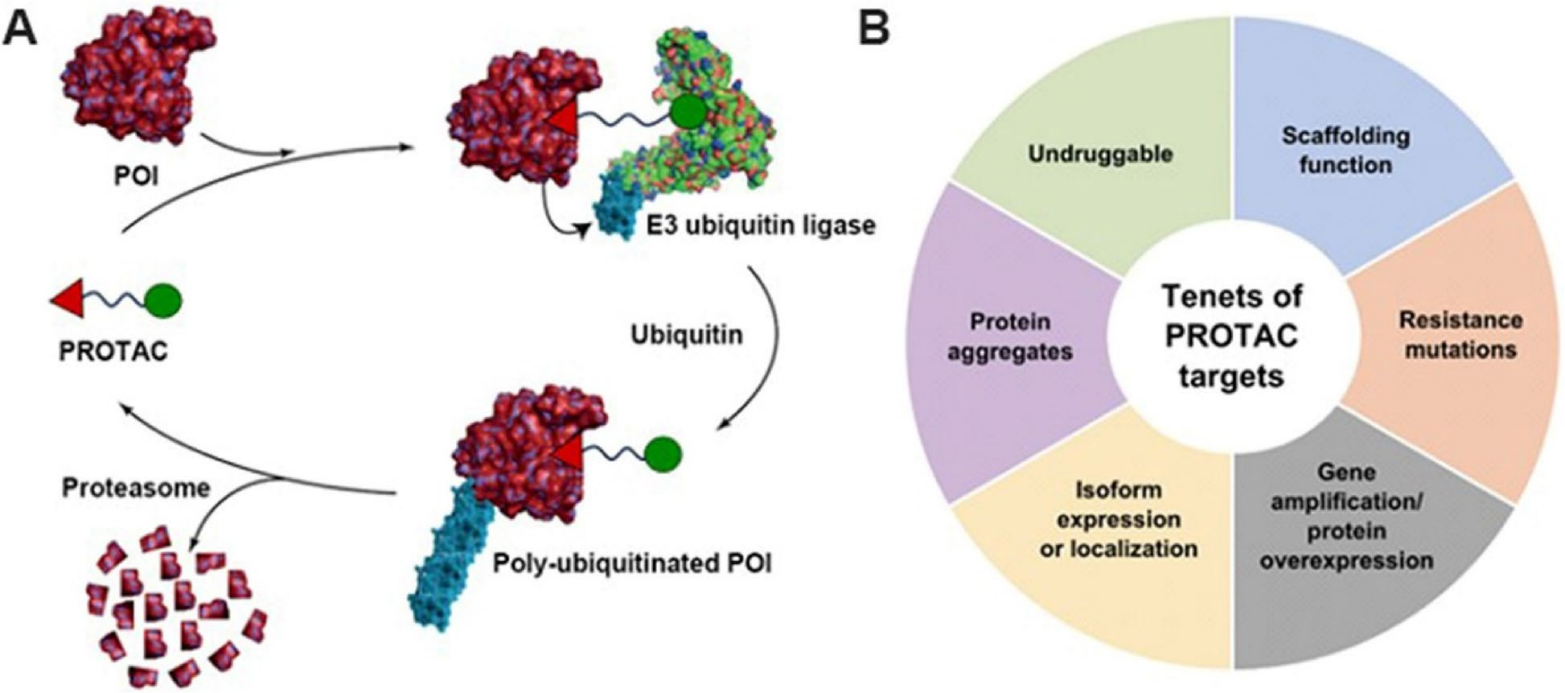
**A** PROTAC-mediated degradation of target proteins through the UPS; **B** The tenets of PROTAC targets

PROTACs effectively redirect the UPS to specifically recognize and degrade proteins of interest (POIs), which frequently play crucial roles in various disease contexts (Fig. 3). This UPS-involved cascade is orchestrated through two essential steps: firstly, the covalent attachment of ubiquitin molecules onto the POIs via tagging; secondly, the subsequent degradation of the polyubiquitinated POIs by the proteasome machinery. The utilization of heterobifunctional molecules facilitates the interaction between E3 ubiquitin ligase and POIs, thereby inducing the successive rounds of ubiquitylation for the substrates. This process ultimately results in the generation of a polyubiquitin chain consisting of four or more ubiquitin units, which is catalyzed by a recruited E2 ubiquitin ligase [18–21]. In view of their distinctive mechanism of action (MOA), PROTACs comprising regulatory ligands for E3 ubiquitin ligases and POIs that are connected by a unique linker offer multiple advantages in regulating POI-related cell function at the molecular level and controlling intracellular biological processes. These low-immunogenic chimeras reversibly and rapidly deplete target proteins with minimal impact on the transcriptome and genome, making them more promising for in vivo applications and potential drug-like properties compared to nucleic acid protein modulation techniques like CRISPR-Cas9 and RNA interference. Moreover, PROTACs possess the remarkable capability of being recycled subsequent to ubiquitination and degradation of the POIs, thereby enabling these compounds to catalyze the elimination of even more POIs [22, 23]. This recyclable attribute exhibited by PROTACs underscores their superiority over conventional small-molecule inhibitors that lack reusability, thus highlighting their potential for advancing therapeutic interventions. Furthermore, PROTACs induce a loss-of-function mechanism by repeatedly and transiently forming ternary complexes comprising a chimera molecule, E3 ubiquitin ligase, and POI. In addition, the binding affinity required for PROTACs is not as stringent or enduring as that needed for small molecule inhibitors which rely on robust occupancy over an extended period of time. Therefore, it is anticipated that numerous PROTACs will effectively surmount the mutation-induced resistance which significantly impacts their small-molecule inhibitor counterparts [9, 24]. Since the induction of proximity between E3 ubiquitin ligases and POIs can be achieved with just two binding ligands, PROTAC-mediated degradation exhibits immense potential in targeting a wide array of proteins, particularly those that were previously deemed 'undruggable'. The activity of PROTACs is primarily dictated by the affinity between chimeras and POIs, as well as their interactions with E3 ligases. These two factors intricately influence the stability of the ternary complex, thereby potentially enhancing selectivity over corresponding inhibitors for protein families harboring a conserved active site [25, 26]. Ultimately, the modular design of these PROTACs enables researchers to systematically enhance the physicochemical properties and efficacy of these compounds, thereby facilitating their optimization for potential applications in a more precise and targeted manner.

**Fig. 4:**
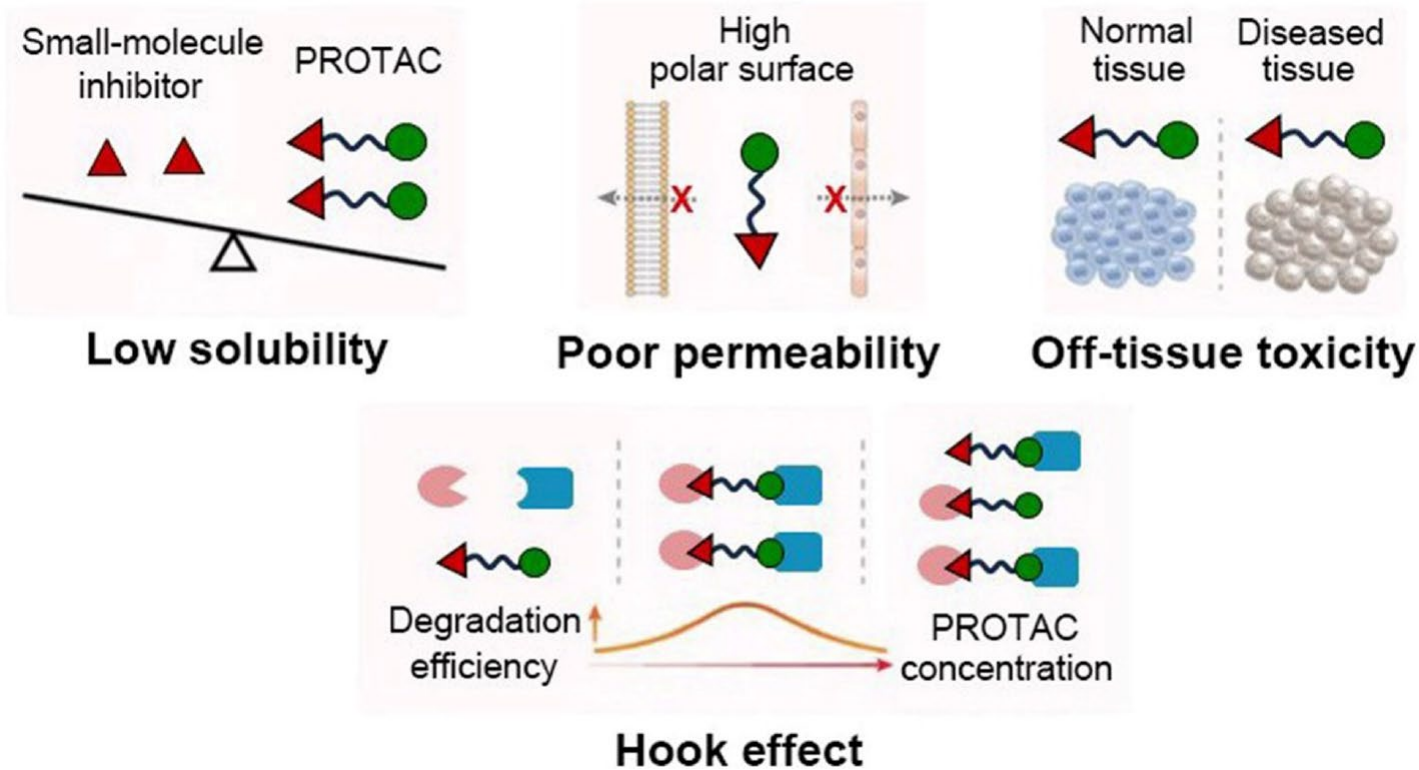
The typical shortcomings of PROTACs

PROTACs possess the potential to revolutionize the realm of drug discovery by offering a remarkably precise and universally applicable strategy for targeting POIs. Nevertheless, substantial challenges persist, and various limitations hinder their clinical applicability (Fig. 4) [27, 28]. Firstly, the occurrence of serious side effects is primarily attributed to off-target biodistribution of PROTACs resulting from non-selective expression of E3 ubiquitin ligases at both the targeted normal tissues and disease site. For example, while the inhibition of Bromodomain and Extra-Terminal (BET) is relatively well-tolerated, complete elimination of these components may lead to evident deterioration in lethargy, skin health, reduced mobility, and spinal hunching as observed in a study involving mice treated with BET PROTAC known as ARV-771 [29]. Secondly, the poor aqueous solubility of PROTACs with a large molecular weight (> 800 Da) often leads to low systemic bioavailability [30]. Thirdly, the high PROTACs' polar surface restricts their permeability, greatly hindering their ability to traverse the cell membrane and physiological barriers [31]. Additionally, the Hook effect—whereby higher intracellular concentrations of PROTACs leading to a higher formation of unproductive binary complexes rather than ternary complexes, compromises the efficacy of target degradation and poses challenges for the rational design of in vivo dosages when precise control over local disease site availability cannot be achieved [32]. Collectively, the non-specific biodistribution, suboptimal solubility, low bioavailability, limited permeability, and unpredictable Hook effect pose significant challenges to the clinical translation of PROTACs.

**Fig. 5:**
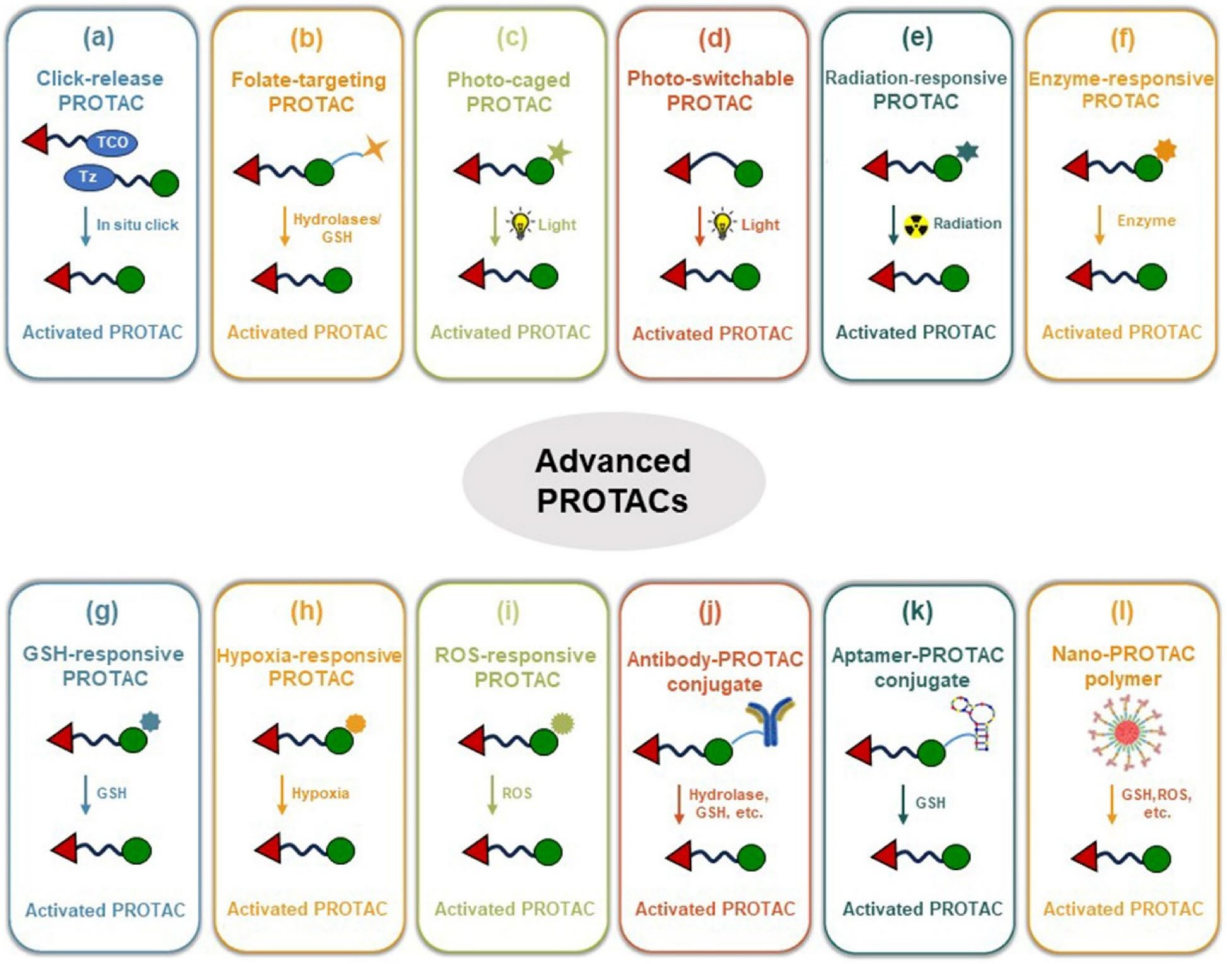
Overview of strategies utilized in the advanced PROTAC design

While structural modifications within PROTAC molecules hold promise in overcoming certain limitations, the simultaneous enhancement of all physicochemical properties for effective in vivo applications poses a formidable challenge [33–35]. Instead of relying solely on excessive chemical optimization, the design of the new-generation advanced PROTACs can potentially address these dilemmas (Fig. 5). These new-generation PROTACs exhibit restored functionality for degradation of POIs upon stimulation by either exogenous or endogenous stimuli in specific tissues, while remaining inactive elsewhere. This innovative approach holds promise for enabling highly targeted therapies with reduced side effects [34, 36–40]. For example, the click-release PROTAC prodrugs, enzyme-responsive PROTAC prodrugs, glutathione (GSH)-responsive PROTAC prodrugs, hypoxia-responsive PROTAC prodrugs, photo-activatable PROTAC prodrugs, radiation-responsive PROTA prodrugs, reactive oxygen species (ROS)-responsive PROTAC prodrugs, etc. Furthermore, the advanced PROTACs also exhibit the capability to selectively target particular cells through ligand optimization, encompassing folate, antibody, and aptamer moieties. In addition, the utilization of nanomedicine delivery system in PROTACs offers several advantages, including enhanced accumulation of PROTACs in diseased tissues and improved pharmacokinetic (PK) profile in vivo. This is exemplified by the application of nano-PROTAC polymers. To provide a comprehensive overview of the rapidly evolving field of advanced PROTACs for cancer therapy, we present an in-depth analysis of recent advancements in PROTAC discovery and the development of new-generation PROTACs (small-molecule PROTAC prodrugs, biomacromolecule-PROTAC conjugates, and nano-PROTACs). This review endeavors to augment our comprehension of this burgeoning field and make a substantial contribution to the advancement of PROTAC-based cancer therapies.

## Development of PROTACs

The pioneering concept of PROTACs was initially proposed by Crews et al*.* in 2001, unveiling the first-generation of PROTACs as a captivating heterobifunctional- molecule encompassing an exquisite 10-amino acid phosphopeptide and a potent angiogenesis inhibitor ovalicin [1]. This specific peptide sequence is recognized by the F-box protein β-TRCP E3 ubiquitin ligase, which serves as an E3 ligase subunit within the heterotetrameric Skp1-Cullin-F box complex. As anticipated, the first-generation of PROTACs effectively triggers the degradation of MetAP2 by enlisting β-TRCP E3 ubiquitin ligase. However, the advancement of these first-generation PROTACs was hindered due to their instability and limited cell permeability within biological systems. To overcome these challenges, Crews et al*.* introduced second-generation PROTACs in 2008, which demonstrated successful intracellular degradation of the Androgen Receptor (AR) within HeLa cells at a concentration of 10 μM [41]. This PROTAC combines nutlin to recruit the E3 ubiquitin ligase human homolog of Mouse Double Molecule 2 (MDM2) with the selective AR modulator (SARM). Subsequently, novel small-molecule PROTACs incorporating Inhibitor of Apoptosis (IAPs), von Hippel-Lindau (VHL), Cereblon (CRBN), as well as DDB1 and CUL4-related factors (DCAF15, DCAF16) ligands have emerged, gaining significant traction in the field. These advancements offer tremendous potential for the exploration and creation of innovative therapeutic agents [42–44]. Despite recent attempts to advance the clinical application of second-generation PROTACs, the majority of these compounds fail to progress beyond the preclinical stage in drug development. One of the most formidable challenges lies in achieving precise protein degradation of desired targets, which remains inadequately addressed. In response to the growing demand for expediting the translational process, the new-generation of advanced PROTACs has been investigated, including small-molecule PROTAC prodrugs, biomacromolecule-conjugated PROTACs, and nano-PROTAC polymers, with the aim of enhancing in vivo performance. These improved new-generation PROTACs have the capability to mitigate unfavorable physicochemical properties associated with traditional PROTACs, enhance their targetability, and minimize off-target side effects. The advent of these advanced and precise new-generation PROTACs will mark a significant milestone in targeted protein degradation field, paving the way for a promising future.

## Small-molecule PROTAC prodrugs

### Click-release PROTAC prodrugs

**Fig. 6:**
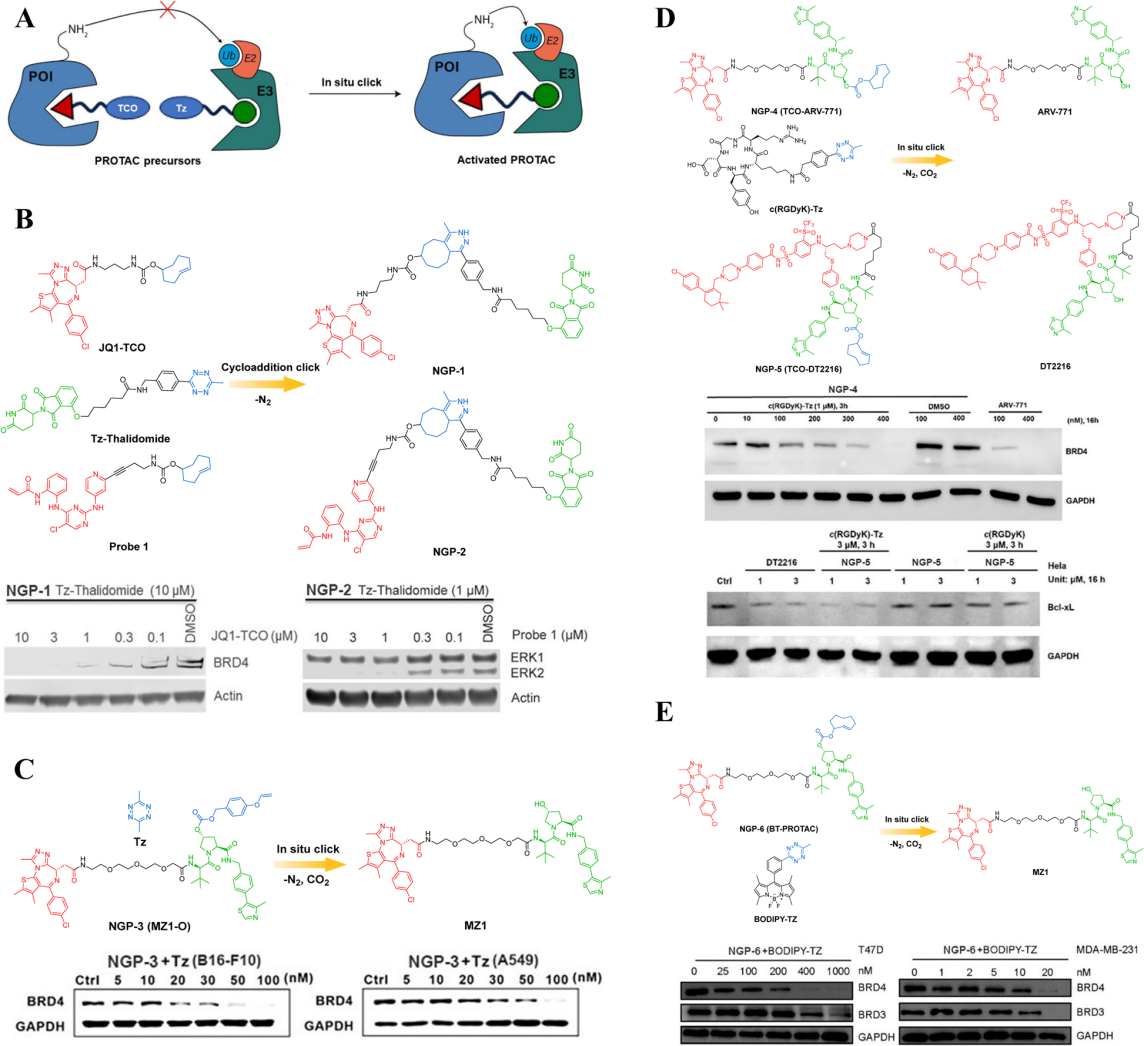
**A** Cartoon showing the structure of the click-release PROTAC prodrugs.** B** Chemical structures of the click-release PROTAC prodrugs (adapted from [49, 50]). **C** Chemical structures of the click-release PROTAC prodrug (adapted from [51]).** D** Chemical structures of the click-release PROTAC prodrugs (adapted from [52]).** E** Chemical structures of the click-release PROTAC prodrug (adapted from [53])

Traditional PROTACs often exhibit inadequate water solubility, tissue permeability, and off-target side effects primarily due to their distinctive molecular composition and structural characteristics [45–48]. To address these limitations and enhance the practicality of PROTACs as therapeutic agents, a modular reactive prodrug (click-release PROTAC prodrug, Fig. 6A) strategy could be implemented. This approach involves generating a heterobifunctional molecule within cells by combining two smaller precursors. By doing so, this strategy offers several advantages. Firstly, using smaller precursors allows for better control over the physicochemical properties of the resulting molecule. This means that issues such as water solubility and tissue permeability can be optimized during precursor selection and design. Secondly, employing a modular reactive prodrug strategy enables greater flexibility in targeting specific proteins while minimizing off-target effects. The use of two distinct precursors provides an opportunity to fine-tune selectivity towards desired target proteins while reducing interactions with non-specific cellular components. Moreover, this approach allows for potential modifications or adjustments in response to emerging scientific knowledge or new drug development strategies without completely redesigning the entire compound structure from scratch. Implementing a modular reactive prodrug strategy offers a more practical approach to overcome challenges associated with traditional PROTACs.

For instance, Lebraud et al*.* devised a click-release PROTAC prodrug (NGP-1) comprising of a *trans*-cyclo-octene ligand specific to the Bromodomain-Containing Protein 4 (BRD4) and a Tetrazine (Tz)-tagged thalidomide derivative [49]. NGP-1 (Fig. 6B) could be efficiently produced within cells by employing click chemistry, specifically the reaction between a small precursor containing Tz group and another *trans*-cyclo-octene small precursor. Another prerequisite for the activity of POI degradation in formed NGP-1 is the formation of an anticipated ternary complex, which relies on the lengths and positions of the linker between the two ligands [50]. A comprehensive screening of tagged groups was conducted to discover a CRBN-JQ1 based NGP-1 with a linker containing 25 separate bonds, encompassing short carbamate and methylene functionalities. In addition to the BRD4 protein, other oncoproteins such as Extracellular Signal-Regulated Protein Kinase 1/2 (ERK1/2) have also been utilized to showcase the modifiable efficacy of the click-release strategy. However, it is worth mentioning that these findings solely pertain to cellular-level demonstrations and do not address the limitations associated with PROTAC's high polar surface area and molecular weight, which hinder its penetration and solubility. Moreover, in the intracellular-generated PROTAC strategy for POI degradation, it is crucial to minimize or eliminate the extracellular combination reaction of precursors as it significantly compromises the efficiency of degradation. To tackle this issue, Lebraud et al*.* successfully optimized the position and rate of click reaction and designed NGP-2 (Fig. 6B) by separately administering the two precursors with an optimal order and time interval. This approach effectively mitigated the premature formation of PROTACs, demonstrating a significant advancement in the field of targeted protein degradation. However, further investigation is needed to determine the optimal dosage regimen of the two precursors in order to enhance the intracellular generation efficiency of PROTACs. The varying pharmacokinetic profiles of these precursors should be carefully considered in this process, as they can significantly impact their efficacy and potential therapeutic outcomes. The potential clinical application of this approach remains unexplored and requires additional research. It is essential to conduct preclinical studies to evaluate its safety, efficacy, and potential off-target effects before considering its translation into clinical practice. Additionally, exploring different delivery methods and formulations may also be necessary to optimize its bioavailability and tissue distribution for various disease indications. In conclusion, while Lebraud et al*.*'s findings are promising, there is still much work to be done before this approach can be considered for clinical use. Continued research efforts will be crucial in unlocking the full therapeutic potential of PROTAC-based therapies for treating a wide range of diseases.

Another instance of click-release PROTAC prodrugs was reported by Huang et al*. *[51]. They designed and synthesized a bioorthogonal click-release PROTAC (MZ1-O, NGP-3, Fig. 6C) by incorporating benzyl carbonate onto the VHL domain. This strategic design allowed for controlled activation of the PROTAC through an iEDDA reaction, leading to efficient degradation of the target protein. The click-release PROTAC remained inactive in the absence of Tz but could be triggered upon exposure to Tz, resulting in intracellular BRD4 protein degradation and subsequent induction of tumor cell apoptosis. Furthermore, their research demonstrated that systemic administration of NGP-3 encapsulated within tumor-targeting poly(lactic-co-glycolic acid) (PLGA) nanoparticles proved to be an effective method for delivering the drug to subcutaneous tumor sites. Once localized at the tumor site, these nanoparticles could be selectively activated by Tz embedded in a dissolvable MN, thereby facilitating precise initiation of the iEDDA bioorthogonal reaction. The development and successful application of this bioorthogonal click-release PROTAC represent a significant advancement in targeted cancer therapy. It offers potential for more precise and efficient treatment strategies with reduced off-target effects. This innovative approach holds promise for improving patient outcomes and advancing our understanding of targeted protein degradation as a therapeutic strategy in oncology.

To further enhance the application of bioorthogonal chemistry in PROTAC prodrug design, Chang et al*.* have successfully devised two novel click-release PROTAC prodrugs that exhibited selective activation within cancer cells and subsequent release of active PROTAC molecules [52]. Inactive PROTAC prodrugs TCO-ARV-771 (NGP-4, Fig. 6D) and TCO-DT2216 (NGP-5, Fig. 6D) have been strategically designed through the conjugation of a *trans*-cyclooctenes (TCO) bioorthogonal moiety into the VHL ubiquitin ligase ligand, demonstrating a rational approach in their development. The RGD peptide modified with Tz, known as c(RGDyK)-Tz, was designed to specifically target the integrin αvβ3 biomarker found in cancer cells. This modification served as the activating component for click-release of PROTAC prodrugs, enabling targeted degradation of BRD4 and B-Cell Lymphoma-X_L_ (BCL-X_L_) proteins in cancer cells while sparing noncancerous normal cells. The findings from studies evaluating the feasibility of this approach demonstrated that the PROTAC prodrugs exhibited selective activation in an integrin αvβ3-dependent manner, leading to the generation of PROTACs that effectively degraded POIs within cancer cells. The click-release PROTAC strategy holds promise as a universal, non-biological method for inducing targeted cell death in cancer through modulation of the ubiquitin–proteasome pathway. This innovative approach could potentially revolutionize cancer therapy by providing a more precise and effective way to target and eliminate cancerous cells without causing harm to healthy tissues. Further research and clinical trials are needed to fully explore the potential of this strategy, but initial findings are promising and warrant continued investigation into its therapeutic applications.

Recently, Bi et al*.* also developed a new click-release PROTAC prodrug BT-PROTAC (NGP-6, Fig. 6E) [53]. While NGP-6 alone does not exhibit degradation of the BRD4 protein, its activity could be triggered by highly reactive Tz precursors. However, it is worth noting that despite NGP-6 exhibiting 100-fold lower efficiency than MZ1 in degrading BRD4, the antitumor efficacy of NGP-6 remains comparable to that of MZ1. MZ1 is composed of three components: the E3 ubiquitin ligase ligand VHL, the BRD4-targeting warhead JQ1, and a PEG linker that connects these two elements. Previous investigations have demonstrated that the side chain carboxyl of JQ1 was located in the solvent-exposed region and modifications to this segment did not impact the crucial binding interactions with POI. Based on their findings, the authors concluded that the antitumor activity of NGP-6 primarily stemmed from the potent inhibition of BRD4 activity by the JQ1 moiety, rather than through degradation of POI. Therefore, considering the limitation associated with incorporating MZ1 into TCO and its inability to fully mask its antitumor activity, future studies should explore alternative caging groups with substantial steric effects that could potentially impede the binding of the JQ1 moiety to POI. Furthermore, the development of this synthetic methodology opens up new possibilities for the integration of TCO into a wide range of previously reported PROCTACs. This not only provides an alternative approach to designing bioorthogonal click-release PROTACs with reduced toxicity, but also offers potential for enhancing their efficacy and specificity in targeting specific proteins or pathways within cells. By expanding the scope of available tools for targeted protein degradation, this study contributes to the advancement of chemical biology and drug discovery efforts aimed at developing more effective therapeutics for various diseases.

### Folate-targeting PROTAC prodrugs

**Fig. 7:**
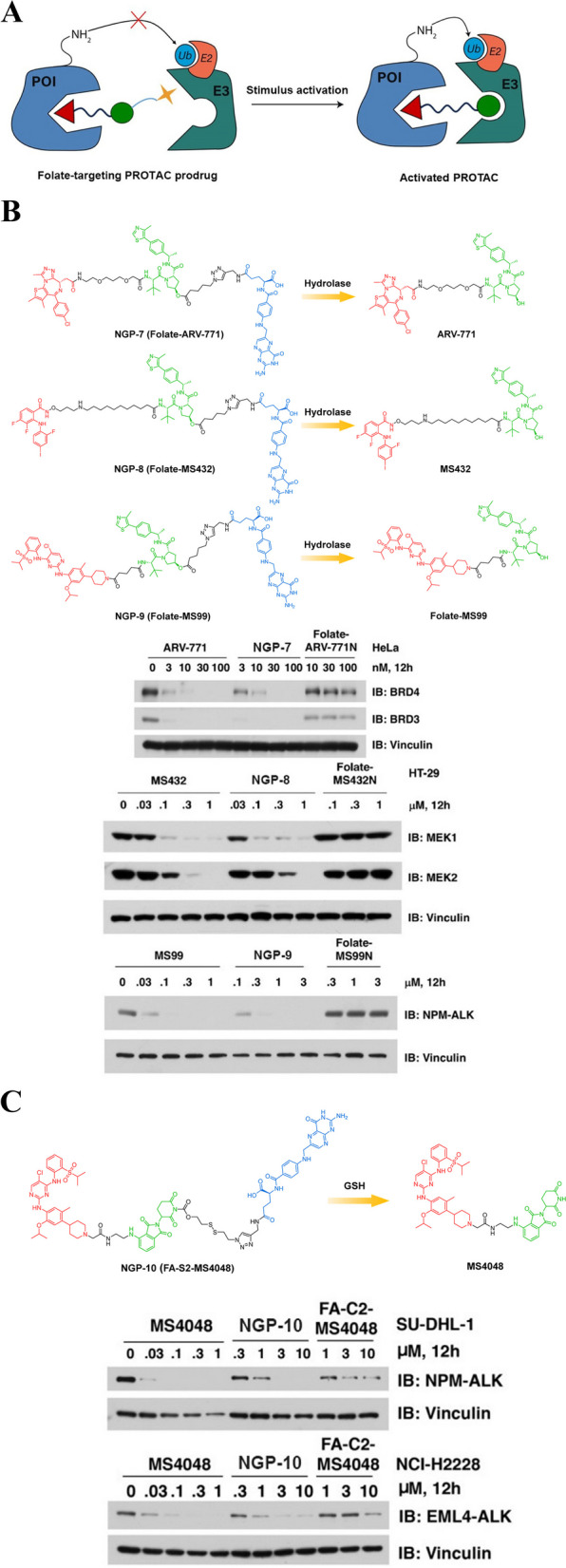
**A** Cartoon showing the structure of the folate-targeting PROTAC prodrugs.** B** Chemical structures of the folate-targeting PROTAC prodrugs (adapted from [57]).** C** Chemical structures of the folate-targeting PROTAC prodrug (adapted from [58])

Folate receptor α (FOLR1 or FRα) has emerged as a protein of immense interest in the realm of anticancer drug delivery, captivating attention as a precise therapeutic target [54, 55]. This is primarily due to its elevated expression levels in various tumor types compared to normal tissues, making it an attractive candidate for targeted therapy. In recent years, researchers have made remarkable progress in developing prodrugs incorporating small-molecule inhibitors with folate [56]. These prodrugs are designed to specifically target cancer cells that overexpress the FRα, while minimizing damage to healthy tissues. The use of folate as a targeting moiety allows for selective delivery of potent anticancer agents directly to tumor cells, enhancing their efficacy and reducing systemic toxicity. Clinical evaluation of these novel prodrugs is currently underway, offering hope for improved treatment options for patients with different types of cancers. Moreover, this approach has also been extended to the design of PROTAC prodrugs (Fig. 7A). By conjugating folates onto PROTAC molecules, they can be directed towards cancer cells expressing FRα while sparing normal tissues from potential harm. This strategy not only improves the safety profile but also enhances the therapeutic window by increasing drug accumulation at tumor sites. It offers a promising avenue for developing more effective targeted therapies against various malignancies while minimizing adverse effects on normal tissues.

Liu et al*.* designed a series of folate-targeting PROTACs to specifically degrade BRDs, Mitogen-Activated Extracellular Signal-Regulated Kinases (MEKs), and Anaplastic Lymphoma Kinases (ALKs) proteins [57]. In particular, NGP-7 (Fig. 7B) was synthesized by attaching folate to the hydroxyl group of a potent BRD4 PROTAC ARV-771 through a triazole linker. This design allows for FRα-mediated internalization and subsequent activation by endogenous hydrolases within the cell. NGP-7 exhibited an effective degradation of BRDs that was dependent on FRα. This suggests that targeting folate receptors could be an effective strategy for selectively degrading specific proteins implicated in diseases such as cancer. Furthermore, Liu et al*.* also synthesized new folate-targeting PROTAC prodrugs NGP-8 (Fig. 7B) and NGP-9 (Fig. 7B), which demonstrated robust degradation specifically targeting MEK and ALK proteins respectively. These findings highlight the versatility of this approach in designing targeted therapies against different protein targets. It is worth noting that the large molecular weight of these folate-targeting PROTAC prodrugs might influence their PK properties and oral bioavailability. The size and complexity of these molecules may affect their absorption, distribution, metabolism, and excretion in the body, which could ultimately influence their efficacy as therapeutic agents. Despite this potential limitation, the results of the study suggest that targeting folate with PROTACs holds promise for broader applicability to other VHL-derived PROTACs. This approach has the potential to expand the scope of targeted protein degradation and provide new opportunities for drug development in various disease areas. Continued research in this area will be crucial for elucidating the optimal strategies for utilizing these compounds in therapeutic interventions. By gaining a deeper understanding of how folate-targeting PROTACs can be effectively utilized, we may uncover novel therapeutic approaches for diseases where selective protein degradation is desired. This could lead to the development of innovative treatment options for conditions such as cancer, neurodegenerative disorders, and autoimmune diseases.

Chen et al*.* pioneered the development of the first pomalidomide-derived folate-targeting PROTAC prodrug NGP-10 [58]. This innovative molecule, as depicted in Fig. 7C, combines a folate moiety with a CRBN-based ALK degrader MS4048 through a disulfide reduction-cleavable linker. Their expertise in designing advanced molecular constructs was evident in this groundbreaking development. The chemical linkage of the folate moiety to the glutarimide motif effectively inhibits the interaction between CRBN and pomalidomide, ensuring precise targeting of the desired proteins. Furthermore, intracellular GSH plays a crucial role in reducing the disulfide bond on NGP-10, leading to spontaneous intramolecular cyclization and subsequent release of MS4048 as an active PROTAC. One particularly noteworthy achievement is that NGP-10 has demonstrated its ability to induce ALK-targeted degradation specifically in FRα + cells. This not only showcases the potential utility of folate-targeting PROTAC prodrugs but also opens up new possibilities for targeted therapy. Overall, Chen et al*.*'s pioneering work with NGP-10 represents a significant advancement in the field of targeted protein degradation and holds promise for future developments in precision medicine and cancer therapy.

### Photo-activatable PROTAC prodrugs

Light is a remarkable external control agent with exceptional spatial and temporal resolution, finding extensive applications in biomedicine, neurobiology, biochemistry, volatile release, fluorescence activation, and polymerization [59]. Photodynamic therapy (PDT) has garnered significant traction as an influential tool for precise drug delivery [60]. The emergence of photo-activatable PROTAC prodrugs represents another exciting development in targeted protein degradation strategies [61, 62]. These innovative compounds combine small molecule ligands that bind disease-causing proteins with an attached photocleavable linker group. Upon exposure to specific wavelengths of light, these linkers are cleaved off, leading to selective degradation of target proteins by cellular machinery. Photo-caged PROTAC prodrugs refer specifically to those compounds where the ligand's activity is masked until it is uncaged upon illumination with appropriate wavelengths of light. On the other hand, photo-switchable PROTAC prodrugs involve ligands that can switch between active and inactive states depending on whether they are exposed or shielded from certain wavelengths of light. Overall, these advancements highlight how harnessing the power of light enables precise control over biological processes and opens up new avenues for therapeutic interventions targeting diseases at their molecular level [63].

#### Photo-caged PROTAC prodrugs

**Fig. 8:**
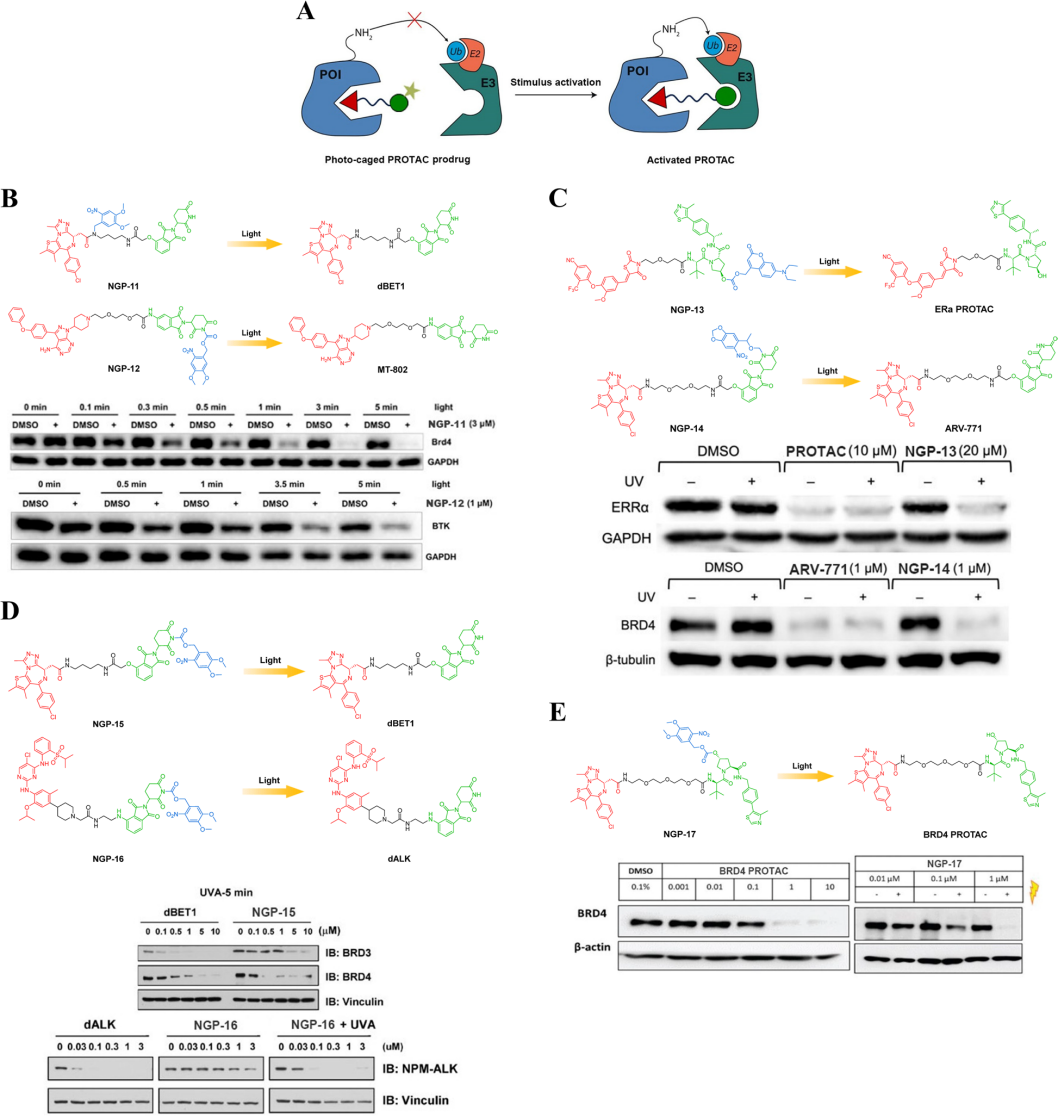
**A** Cartoon showing the structure of the photo-caged PROTAC prodrugs.** B** Chemical structures of the photo-caged PROTAC prodrugs (adapted from [64]).** C** Chemical structures of the photo-caged PROTAC prodrugs (adapted from [65]).** D** Chemical structures of the photo-caged PROTAC prodrugs (adapted from [66]).** E** Chemical structures of the photo-caged PROTAC prodrug (adapted from [67])

Photo-caged PROTAC prodrugs (Fig. 8A) generally incorporate photocleavable caging groups into the parent PROTACs to suppress binding affinities against E3 ubiquitin ligases or POIs under light-free conditions. Subsequently, upon light irradiation, the active PROTACs are promptly liberated, thereby facilitating the degradation of POIs.

Xue et al*.* developed a novel approach to target BRD4 using a photo-caged PROTAC prodrug NGP-11 (Fig. 8B), which involved the incorporation of the 4,5-dimethoxy-2-nitrobenzyl group (DMNB) into the BET binding region of dBET1, a potent BRD4 PROTAC degrader [64]. This strategic modification couldn’t effectively impede the binding capability of BRD4 in the absence of light irradiation, as anticipated. However, the binding affinity was restored upon exposure to UV light at a wavelength of 365 nm, leading to dose-dependent degradation of BRD4 in Ramos cells. Furthermore, in a zebrafish embryo model, NGP-11 demonstrated effective photoinduced degradation of BRD4, resulting in the anticipated phenotypic alterations. Significantly, this strategy was not limited to BRD4 alone; the authors also successfully incorporated the DMNB motif into the imide nitrogen of MT-802, a Bruton's Tyrosine Kinase (BTK) PROTAC, resulting in NGP-12 (Fig. 8B). Remarkably, upon light activation, this compound exhibited remarkable efficacy in inducing potent degradation of BTK within Ramos cells.

Naro et al*.* successfully devised a new photo-caged PROTAC prodrug for the degradation of proteins triggered by light, demonstrating their expertise in this field [65]. The approach involved incorporating a coumarin derivative (DEACM) cage group onto the hydroxyproline of VHL through a carbonate linker, resulting in NGP-13 (Fig. 8C) that specifically targeted Estrogen Receptor α (ERα). Unsurprisingly, the DEACM group in NGP-13 prevented the degradation of ERα under light-free conditions. However, efficient and fast photoactivation of protein degradation was observed during photolysis (≤ 405 nm). For CRBN-based PROTACs, Naro et al*.* incorporated a piperonyloxymethyl (NPOM) moiety onto the glutarimide nitrogen of thalidomide to generate photo-caged BRD4 PROTAC NGP-14 (Fig. 8C). With exposure to light, NGP-14 effectively removed the NPOM group and exhibited potent antiproliferative efficacy in 22Rv1 cells. Therefore, this strategy holds great promise for the development of VHL- and CRBN-based photo-caged PROTAC prodrugs targeting other different kinds of proteins. With further research and development, these photo-caged PROTAC prodrugs could pave the way for more precise and effective treatments for a variety of conditions, ultimately benefiting patients in need of innovative therapeutic options.

Liu et al*.* developed a series of novel photo-caged PROTAC prodrugs by introducing a nitroveratryloxycarbonyl group (NVOC) on the glutarimide nitrogen of the pomalidomide molecule [66]. Following this strategic approach, they successfully synthesized photo-caged NGP-15 (Fig. 8D) utilizing dBET1 as the exemplary template. As anticipated, the CRBN binding affinity of NGP-15 was significantly diminished as a result of the introduction of bulky NVOC substitution. Under UV irradiation at a wavelength of 365 nm, photolysis of NGP-15 resulted in the liberation of the active parent PROTAC (dBET1), thereby facilitating the degradation of BRDs in a dose-dependent manner. Similarly, a photo-caged NGP-16 (Fig. 8D) was devised to enable light-induced degradation of ALK fusion proteins. Consequently, this strategy holds promise as a versatile platform for the design and development of pomalidomide-based photo-activatable PROTAC prodrugs capable of photo-controllable protein degradation. This strategy opens up new possibilities for targeted therapy and drug delivery, offering opportunities for precise manipulation of protein levels within cells. Furthermore, the development of such photo-controllable prodrugs could lead to advancements in the treatment of various diseases, including cancer and neurodegenerative disorders. This innovative strategy holds great promise for improving therapeutic outcomes and expanding our understanding of targeted protein degradation mechanisms.

Kounde et al*.* developed an innovative photo-activatable NGP-17 (Fig. 8E) by incorporating a DMNB group into the crucial hydroxy motif of the VHL-binding moiety [67]. This novel approach allowed for precise control over protein degradation, as the caged form of NGP-17 exhibited no activity in the absence of light. However, upon irradiation at 365 nm, it triggered the degradation of BRD4, a protein involved in gene regulation. To visualize this process in real time, the researchers employed fluorescence imaging techniques using Green Fluorescent Protein (GFP)-tagged BRD4 in live HEK293 cells. The light-induced reduction in GFP-tagged BRD4 was observed and recorded over time, providing valuable insights into the dynamics and efficiency of protein degradation mediated by NGP-17. This study showcases the potential applications of light activation in targeted protein degradation strategies. By harnessing specific wavelengths of light to activate or deactivate therapeutic agents like PROTACs, researchers can achieve spatiotemporal control over protein levels within cells.

#### Photo-switchable PROTAC prodrugs

**Fig. 9:**
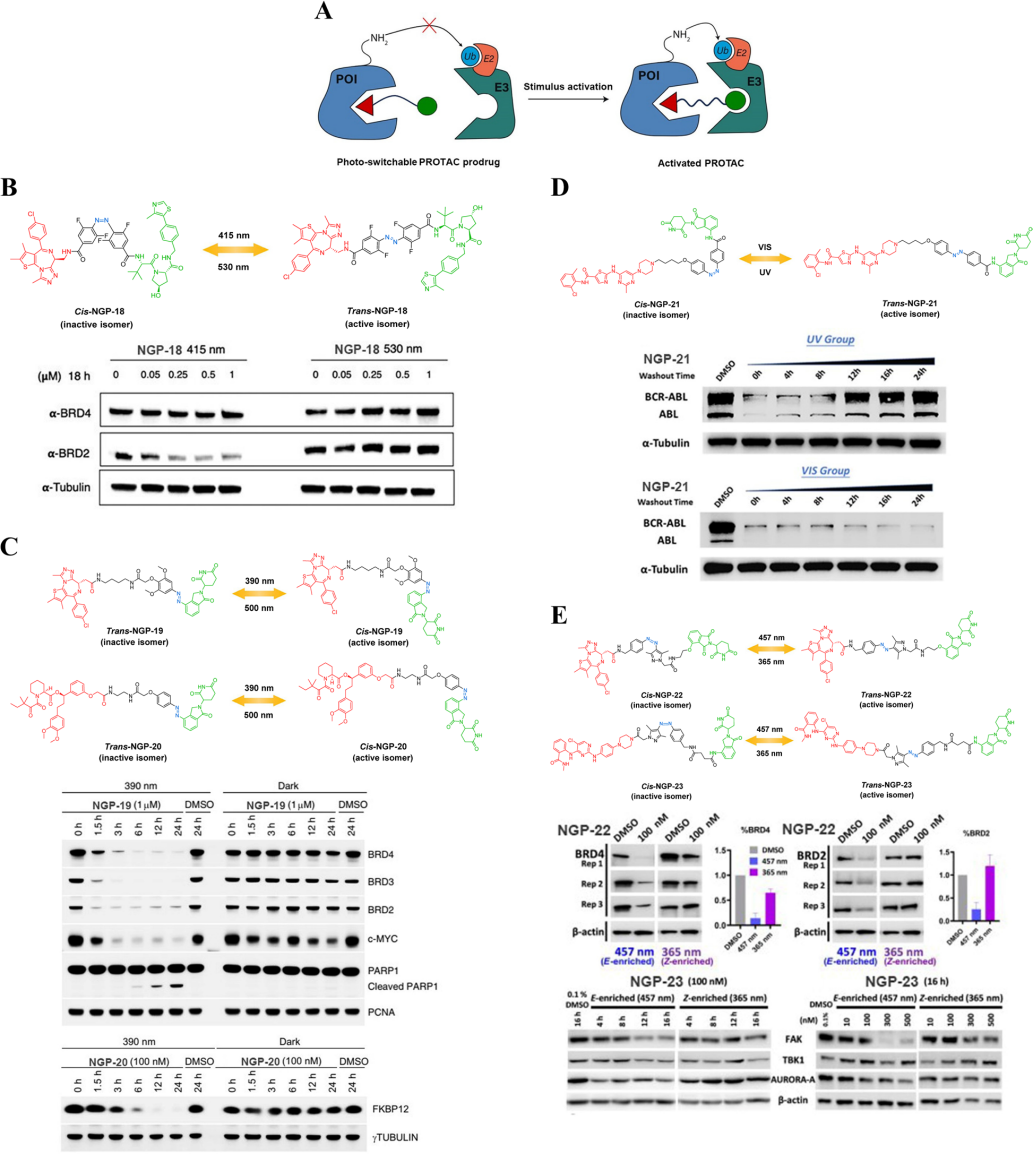
**A** Cartoon showing the structure of the photo-switchable PROTAC prodrugs (adapted from [68]).** B** Chemical structures of the photo-switchable PROTAC prodrugs (adapted from [68]).** C** Chemical structures of the photo-switchable PROTAC prodrugs (adapted from [69]).** D** Chemical structures of the photo-switchable PROTAC prodrugs (adapted from [70]).** E** Chemical structures of the photo-switchable PROTAC prodrugs (adapted from [71])

In addition to the photo-caged PROTACs, there has been a recent exploration of utilizing photo-switchable PROTACs (Fig. 9A) to attain a reversible manipulation of the protein degradation profile offered by the PROTACs [68]. The azobenzene moiety is commonly utilized as the photo-switchable group due to its exceptional resistance to fatigue, adjustable photothermal capability, and predictable geometric transformation. Thus, upon exposure to specific wavelengths of light, photo-switchable PROTACs can efficiently undergo transformation between the "*trans*" and "*cis*" isoforms, resulting in distinct biological activities attributed to significant alterations in the conformational arrangement and topological distance between the warheads of the POIs and the E3 ubiquitin ligase ligands.

Pfaff et al*.* were the first to pioneer the incorporation of photo-switchable handles into VHL-based PROTACs, thereby enabling photo-controllable activity of PROTACs [68]. In this study, they synthesized photo-switchable PROTAC *trans*-NGP-18 (Fig. 9B) by substituting the linear linker in the ARV-771 degrader with a bistable *o*-F4-azobenzene linker. *trans*-NGP-18 efficiently facilitated the degradation of BRD2 in Ramos cells while exhibiting selectivity towards preserving BRD4. In contrast, *cis*-NGP-18 (Fig. 9B) exhibited a shorter topological linkage length, leading to the inhibition of BRD2 degradation over the concentration range tested. Efficient conversion of both isomers was successfully accomplished through irradiation at 415 and 530 nm. Furthermore, it was observed that the photostationary state (PSS) of both isomers remained persistent without undergoing thermal back-isomerization, thereby demonstrating the dynamic switching capability of NGP-18 between these two states to induce distinct bioactivities. The precise spatiotemporal regulation of photoinduced degradation of target proteins using photo-switchable PROTACs incorporating an *o*-F4-azobenzene-containing linker represents a valuable tool for investigating intricate protein signaling pathways that remain poorly understood.

Reynders et al*.* conducted an investigation into the elaborate design of photo-switchable PROTACs and successfully synthesized *trans*-NGP-19 (Fig. 9C) by incorporating an azobenzene moiety into the lenalidomide component of a JQ1-based PROTAC [69]. In this particular case, it was observed that *trans*-NGP-19 did not exhibit any activity and did not have any impact on the degradation of BET proteins under dark conditions. However, an interesting phenomenon occurred when *trans*-NGP-19 was exposed to irradiation at 390 nm. It underwent a conversion into *cis*-NGP-19 (Fig. 9C), which had a significant effect on reducing the protein levels of BRD2-4 as well as Cellular-Myelocytomatosis Viral Oncogene (c-MYC). It is worth noting that *cis*-NGP-19 exhibited its inhibitory effects gradually over time and eventually reverted back to its inactive form. This observation suggests that the isomerization process from *cis*-NGP-19 to *trans*-NGP-19 is relatively slow. These findings provide valuable insights into the behavior and potential therapeutic applications of these compounds in regulating protein levels associated with BRD2-4 and c-MYC. Further investigations are imperative to unravel the intricate mechanisms underlying this photo-induced transformation and its implications for targeted therapies in various diseases where BET proteins play a crucial role. Using a similar approach, photo-switchable PROTAC *trans*-NGP-20 (Fig. 9C) achieved photocontrol of FKBP12 degradation in RS4;11 cells, suggesting that this strategy holds promise for the design of photo PROTACs to mitigate potential systemic toxicity.

Jin et al*.* developed photo-switchable PROTAC NGP-21 (Fig. 9D) as a novel photo-switchable PROTAC prodrug to target Breakpoint Cluster Region-Abelson Leukemia Virus (BCR-ABL) fusion and Abelson Leukemia Virus (ABL) proteins [70]. This novel compound presents a distinctive benefit by allowing the adjustability of both the orientation and length of its *azo*-linker under varying wavelengths of light. Upon treatment with *trans*-NGP-21, remarkable reductions in the levels of BCR-ABL and ABL proteins were observed, while no discernible changes in protein levels were detected for the corresponding *cis* isomer. Upon exposure to visible light, the inactive *cis*-NGP-21 underwent a remarkable activation and effectively triggered the degradation of POIs within the BCR-ABL-driven K562 cell line. Conversely, UV irradiation of live cells caused *trans*-NGP-21 to revert back to its inactive state. By targeting ABL and BCR-ABL fusion proteins, NGP-21 holds promise in treating diseases associated with these aberrant proteins such as Chronic Myeloid Leukemia (CML). The ability to selectively degrade these disease-causing proteins using light-controlled adjustments could potentially lead to more effective therapies with fewer side effects. Jin et al*.*'s development of NGP-21 represents an exciting advancement in protein targeting technology. Its adjustable *azo*-containing linker under different wavelengths of light offers researchers a powerful tool for investigating protein function while holding potential therapeutic implications for diseases involving ABL and BCR-ABL fusion proteins.

Zhang et al*.* developed a novel series of photo-switchable PROTACs by incorporating a photoswitch arylazopyrazole into the linker moiety [71]. Remarkably, *trans*-NGP-22 (Fig. 9E) exhibited remarkable degradation efficacy against BRDs, while its *cis* isomer failed to demonstrate any discernible effect in this regard. In addition, NGP-23 (Fig. 9E) represents a significant advancement in the field of targeted protein degradation. This represents a groundbreaking instance of a photo-switchable degradation activity in a multikinase targeting PROTAC. NGP-23 was specifically designed to target a wide range of kinases, and it successfully captured 235 different kinases across diverse families using its recruited BET ligand (CTX-0294885). However, despite its impressive kinase capturing abilities, *trans*-NGP-23 only exhibited degradation activity towards three specific kinases: Aurora Kinase A (AURORA-A), Focal Adhesion Kinase (FAK), and TANK Binding Kinase 1 (TBK1). This work suggests that new arylazopyrazole photo-switchable PROTACs have the potential to achieve a high abundance of PSS isomer, swift switching capability, and an optimal half-life (T_1/2_). Further research and development are needed to fully understand the mechanism behind this selectivity and explore the full therapeutic potential of photo-switchable PROTACs. Nonetheless, this pioneering work opens up new possibilities for designing future PROTACs with enhanced specificity towards desired targets while minimizing off-target effects.

### Radiation-responsive PROTAC prodrugs

Radiotherapy, also known as radiation therapy, is a widely used and highly effective treatment for cancer [72–74]. This form of treatment is often considered a first-line option due to its ability to provide precise targeting and deep tissue penetration [75]. One of the important advantages of radiotherapy is its ability to deliver localized release of X-ray-responsive prodrugs in tumor tissue. By combining radiotherapy with these X-ray-responsive prodrugs, it becomes possible to enhance the antitumor effects and potentially achieve superior outcomes. The synergistic effects between active drugs and X-rays further contribute to the effectiveness of radiotherapy. When combined with certain chemotherapy agents or targeted therapies, the simultaneous administration of these treatments can lead to enhanced tumor cell killing. This combination approach has shown promising results in clinical trials, offering new hope for patients battling various types of cancers.

**Fig. 10:**
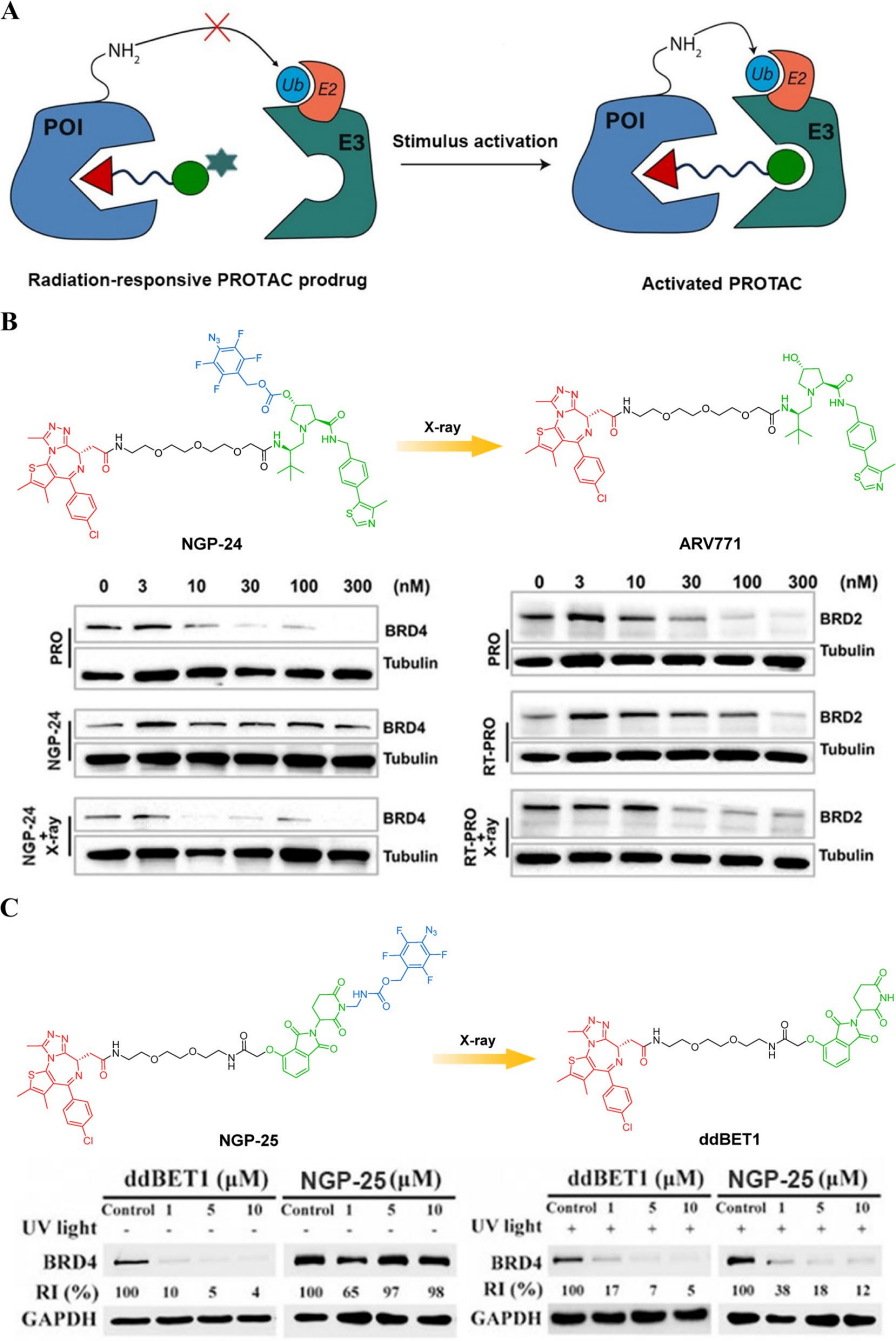
**A** Cartoon showing the structure of the radiation-responsive PROTAC prodrugs (adapted from [68]).** B** Chemical structures of the radiation-responsive PROTAC (adapted from [76]).** C** Chemical structures of the radiation-responsive PROTAC (adapted from [77])

Yang et al*.* have made a new innovation in the field of radiotherapy by developing a novel approach called radiation-responsive PROTAC (Fig. 10A) [76]. This innovative technique involves incorporating an azide-caged into the VHL ligand of the PROTAC moiety, enabling precise and spatiotemporal degradation of specific proteins. To demonstrate the feasibility of their approach, the researchers designed a derivative of ARV771 serving as an exemplary PROTAC prodrug model. This involved modifying the hydroxyl group of VHL ligand with a (4-azido-tetrafluorophenyl) methanol mask group through a carbonate bond. The purpose of this modification was to block the interaction between radiation-responsive PROTAC and E3 ligase. Upon exposure to X-ray radiation, the mask moiety underwent reduction, resulting in the formation of 4-(hydroxymethyl)-tetrafluoroaniline. Subsequently, NGP-24 (Fig. 10B) was further eliminated through a decarboxylation and elimination reaction. Consequently, NGP-24 was restored to its original form as ARV771 for efficient degradation of the BET. The cleavage of the caged group induced by X-ray radiation was confirmed through ultra-performance liquid chromatography analysis. Western blot assay revealed that NGP-24 had minimal impact on the expression of the POI in the absence of radiation. However, in contrast, significant degradation of POI occurred under conditions of X-ray radiation. Importantly, it is noteworthy that a synergistic inhibition of tumor growth was observed when combining X-ray radiation with NGP-24 treatment in an MCF-7 breast tumor-bearing mouse model. Overall, their pioneering work on developing radiation-responsive PROTAC holds immense promise for improving cancer treatment outcomes through precise and spatiotemporal protein degradation. Their innovative approach may revolutionize how we combat not only cancer but also other diseases characterized by aberrant protein levels.

In a new study, An et al*.* have made significant advancements in the field of controllable targeted drug design by developing a novel radiation-responsive PROTAC prodrug NGP-25 (Fig. 10C) for traceless release [77]. This innovative approach utilizes carbamate-bearing cages that exhibit excellent chemical and proteolytic stabilities, ensuring the stability of NGP-25 in blood. In comparison to other molecules bearing carbonate or ester bonds, these cages provide enhanced stability. The researchers conducted experiments to confirm the efficacy and safety of their radiation-responsive PROTAC prodrug. They found that precise and efficient activation of the prodrug could be achieved through safe dosages of X-ray irradiation. This activation specifically targeted BRD4 degradation in tumors while having minimal impact on normal organs. Such selective targeting is crucial for minimizing side effects and maximizing therapeutic benefits. One particularly noteworthy aspect of this study is its potential application beyond BRD4 degradation in tumors. The caging strategy employed by An et al*.* can be extended to other disease-related biomarkers such as cathepsin B, aminopeptidase N, and β-galactosidase. This opens up exciting possibilities for designing activatable PROTACs, antibody–drug conjugates (ADCs), prodrugs, and biomaterials tailored towards personalized treatment approaches. Overall, this research represents an important step forward in the development of targeted therapies with improved stability and selectivity. By harnessing radiation responsiveness and utilizing advanced linker chemistry techniques, An et al*.*'s work has paved the way for more effective treatments that minimize off-target effects while maximizing therapeutic outcomes across various diseases.

### Tumor microenvironment-responsive PROTAC prodrugs

#### Enzyme-responsive PROTAC prodrugs

**Fig. 11:**
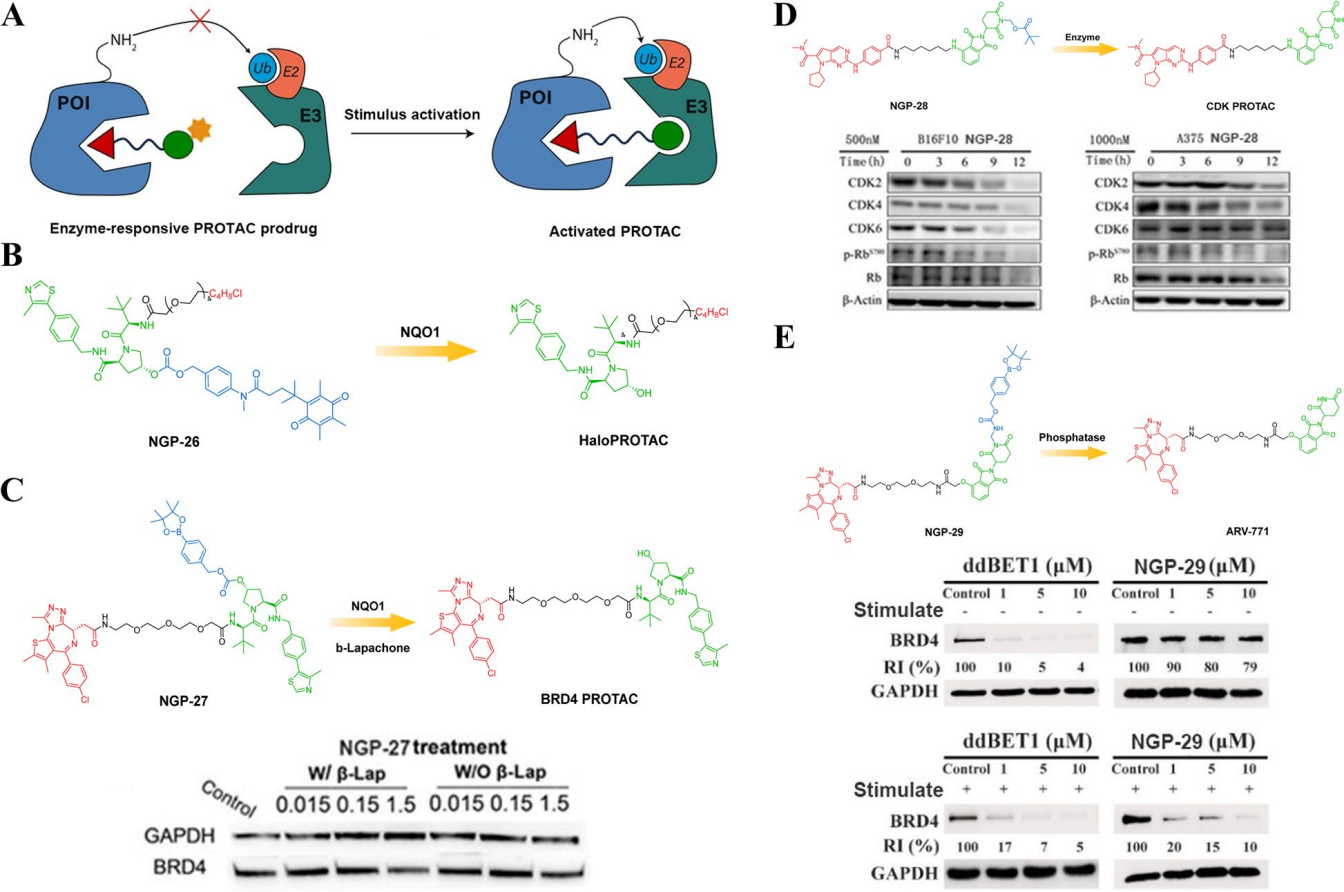
**A** Cartoon showing the structure of the enzyme-responsive PROTAC prodrugs (adapted from [68]).** B** Chemical structures of the enzyme-responsive PROTAC (adapted from [81]).** C** Chemical structures of the enzyme-responsive PROTAC (adapted from [81]).** D** Chemical structures of the enzyme-responsive PROTAC (adapted from [82]).** E** Chemical structures of the enzyme-responsive PROTAC (adapted from [77])

Due to the very different microenvironment in which diseased and normal tissues are located, cancer cells exhibit several endogenous hallmarks that differentiate them from healthy cells [78, 79]. One of these hallmarks is the overexpression of a large variety of enzymes [80]. The overexpression of enzymes in cancer cells opens up new possibilities for targeted therapies. By designing PROTAC prodrugs that are specifically activated by these overexpressed enzymes, researchers can selectively degrade proteins within cancer cells while sparing normal cells. This approach holds great promise in improving the efficacy and safety of cancer treatments. Harnessing the abundance of overexpressed enzymes in cancer cells presents an exciting avenue for developing novel therapeutics based on enzyme-responsive PROTAC prodrugs (Fig. 11A). By capitalizing on this opportunity, scientists can advance our understanding of cancer biology while potentially revolutionizing treatment options for patients affected by this devastating disease.

Given that, Liang et al*.* developed a controllable protein degradation strategy that could selectively target cancer cells [81]. To achieve this, they combined enzyme-responsive chemistry with a PROTAC approach. The researchers incorporated a trimethyl-locked quinone group into the BRD4-targeted PROTAC, which they named Pro-PROTAC (NGP-26, Fig. 11B). The unique feature of the trimethyl-locked quinone group is its ability to be reduced and removed by an enzyme called NAD(P)H quinone dehydrogenase 1 (NQO1), which is known to be overexpressed in tumor cells. This design allowed the NGP-26 to remain inactive or "inert" in normal tissues where it binds to E3 ligase VHL. However, once inside cancer cells with elevated levels of NQO1, the masked quinone group was reduced by NQO1, leading to self-immolating cleavage and release of the active HaloPROTAC. By incorporating this enzymatic activation mechanism into their design, Liang et al*.* were able to achieve cell-selective protein degradation. The presence of NQO1 in cancer cells triggered the removal of the trimethyl-locked quinone group from NGP-26, thereby restoring its ability to degrade targeted proteins through interaction with E3 ligase VHL. This innovative strategy holds great promise for future therapeutic applications as it allows for precise control over protein degradation specifically within cancer cells while sparing normal tissues. Further research will undoubtedly explore how this approach can be optimized and applied in various disease contexts for more effective treatment options.

To further enhance the selectivity of protein degradation mediated by NQO1, Liang et al*.* developed a novel approach that utilizes an NQO1-regulated cascade reaction to specifically activate small-molecule PROTACs in cancer cells [81]. The key idea behind this strategy is to differentiate and potentiate the intracellular environment of cancer cells, making them more susceptible to the action of PROTACs. In previous studies, it has been demonstrated that the reduction of β-Lapachone by NQO1 can generate abundant ROS within living cells [83–85]. This observation inspired the researchers to design a NQO1-responsive PROTAC called NGP-27 (Fig. 11C). In this design, BRD4 PROTAC was chemically caged using an aryl boronic ester that can be cleaved by ROS. Only when both NQO1 and β-Lapachone are overexpressed simultaneously in cancer cells, the NGP-27 becomes activated and effectively degrades BRD4. Compared to the activation mechanism of another type of PROTAC called NQO1-HaloPROTAC, which solely relies on cellular NQO1 activity for removing trimethyl-locked quinone, the activation of NGP-27 requires not only the presence of NQO1 but also β-Lapachone. This dual requirement significantly improves the cell selectivity of NGP-27 as it ensures that only cancer cells with both elevated levels of NQO1 and β-Lapachone will undergo targeted protein degradation. The development and application of such innovative strategies hold great promise for advancing precision medicine approaches in treating various diseases including cancer.

Apart from NQO1, there is a wide range of other highly expressed enzymes in diseased cells that can be harnessed for enzyme-responsive PROTAC prodrug strategies [82]. These enzymes offer an expanded repertoire in the field of chemical biology, enabling cell-specific protein degradation and potentially revolutionizing targeted drug discovery. By leveraging these abundant overexpressed enzymes, researchers can design PROTAC prodrugs that selectively target disease-associated proteins within specific cell types. This approach holds great promise for developing more effective therapies with reduced off-target effects. For proof-of-concept, Wei et al*.* synthesized a new Cyclin-Dependent Kinases (CDK) degrader prodrug NGP-28 (Fig. 11D) as a model enzyme-responsive PROTAC prodrug [82]. The CRBN ligand’ amino group of NGP-28 was modified with the mask moiety of methyl pivalate via mild condition to block the interaction between the CDK PROTAC and E3 ligase. At the tumor site in vivo, the mask moiety was partially removed under the catalysis of the enzyme, releasing the active E3 ubiquitin ligase ligand molecule. Subsequently, NGP-28 was restored to active CDK PROTAC for degradation of the POI. Western blot assay demonstrated that NGP-28 barely affected CDK expression in vitro. In contrast, the CDK was markedly degraded in vivo. It was worth noting that NGP-28 synergistically inhibited tumor growth in B16F10 melanoma-bearing mouse model. This is the first development of an orally bioavailable NGP-28 with high bioavailability for oral administration testing in animals. It may also provide a generic solution for oral administration of PROTAC molecules.

An et al*.* developed a novel and versatile approach for synthesizing a phosphatase-responsive PROTAC prodrug with diverse molecular blocks that possess both robustness and cleavable linkers [77]. This strategy allows for the precise manipulation of protein degradation by introducing a "turn on" feature. By taking advantage of the pathological cue of elevated phosphatase levels, the researchers were able to achieve site-specific activation and untraceable release of the original PROTAC molecule through de-caging and subsequent self-immolative cleavage. This breakthrough enables selective uptake and controlled degradation of target proteins in vitro. The study indicated that this particular NGP-29 (Fig. 11E) demonstrated long plasma exposure and high solubility, making it an ideal candidate for targeted therapy. What makes NGP-29 even more remarkable is its specific activation by tumor cells that overexpress phosphatase enzymes. The activation of NGP-29 leads to efficient protein degradation within the tumor cells, ultimately resulting in potent tumor remission. This finding opens up new possibilities for personalized treatment approaches in cancer therapy. Furthermore, as more reactive biomarkers are being discovered through clinical practice, there is a growing need for versatile tools to design activatable PROTACs, smart biomaterials, and prodrugs. In this context, the caging library developed by researchers could serve as a valuable resource.

#### GSH-responsive PROTAC prodrugs

Tumor microenvironment-responsive strategy is a promising approach that allows for specific control of the PROTAC's on-target degradation activity. This strategy takes advantage of the unique characteristics of tumor cells, such as their increased levels of GSH [86–89]. GSH has been reported to be significantly elevated in tumor cells compared to normal tissues [90–92]. In the context of breast cancer (BC) therapeutics, ERα-targeting PROTACs have emerged as a promising and novel modality. These PROTACs are designed to selectively degrade ERα. However, one concern with ERα PROTACs is their potential off-tissue toxicity, meaning they may induce unwanted degradation in normal tissues.

**Fig. 12:**
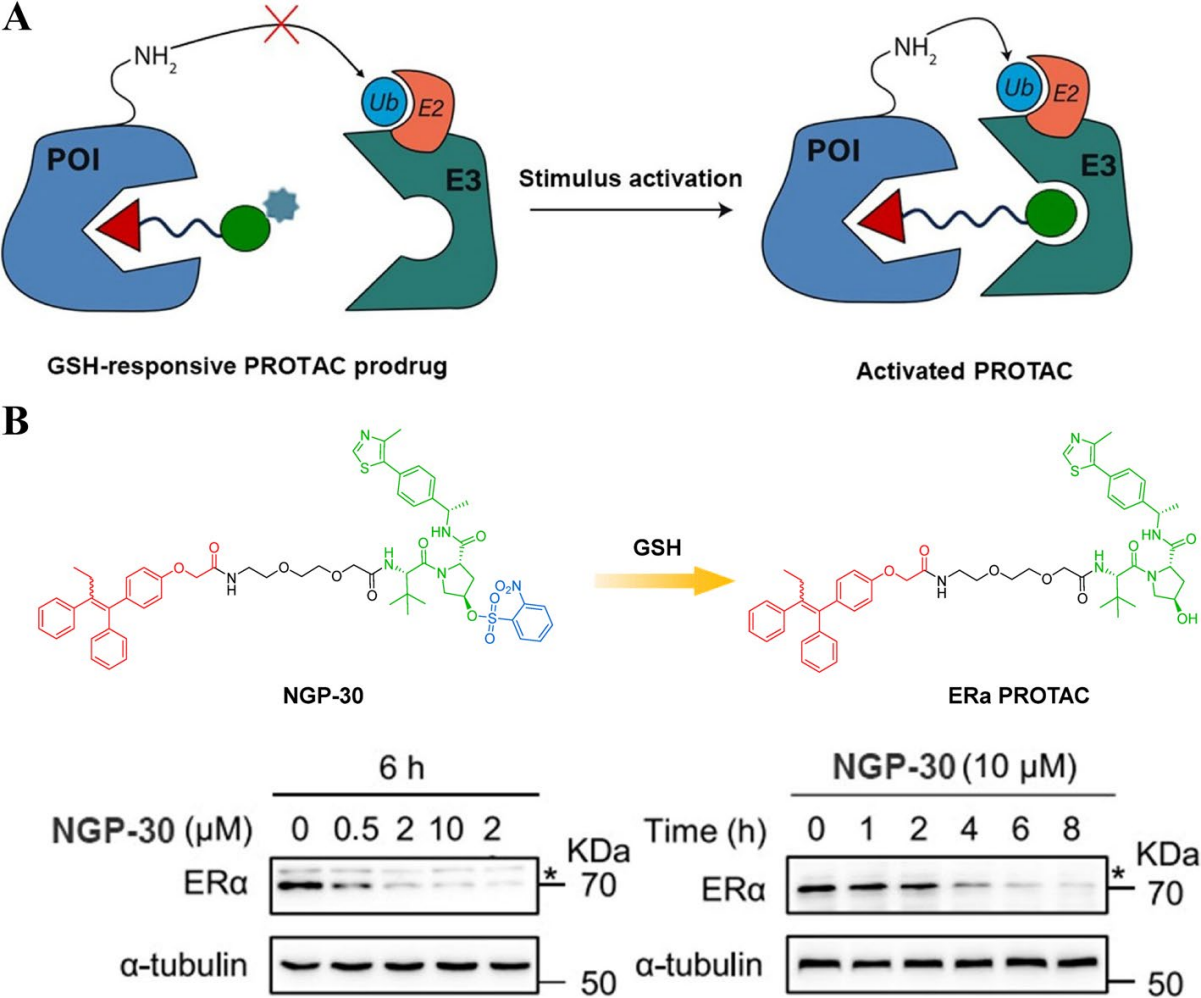
**A** Cartoon showing the structure of the GSH-responsive PROTAC prodrugs (adapted from [68]).** B** Chemical structures of the GSH-responsive PROTAC prodrug (adapted from [93])

To address this concern, Zhou et al*.* developed a GSH-responsive ERα PROTAC (Fig. 12A) [93]. They achieved this by conjugating an *o*-benzenesulfonyl group to the hydroxyl group of PROTAC targeting ERα through a nucleophilic substitution reaction. This design allows for selective activation and degradation of ERα only in tumor cells with high GSH levels. The* o*-benzenesulfonyl group, serving as a protective moiety, effectively impedes the bioactivity of ERα PROTAC, which can be specifically recognized and eliminated by the abundant presence of GSH in cancer cells. One major advantage of using GSH-responsive PROTACs like NGP-30 (Fig. 12B) is their ability to selectively target cancer cells while sparing normal cells from toxic effects. This selectivity reduces potential side effects and improves the overall safety profile of the treatment. This study highlights the potential value of utilizing tumor microenvironment-responsive PROTACs for BC treatment. By exploiting specific characteristics or components present within cancer cells, such as high levels of GSH, these innovative therapeutic strategies offer new possibilities for more effective and personalized approaches against BC.

#### Hypoxia-responsive PROTAC prodrugs

Hypoxia is a distinctive characteristic observed in numerous solid tumors and is associated with unfavorable prognosis and drug resistance [94]. The expression of Nitroreductase (NTR) is elevated in hypoxic solid tumors compared to healthy tissues under normoxic conditions, thereby conferring NTR-responsive prodrugs with excellent selectivity between normal cells and tumor cells [95]. Evofosfamide (TH-302 or Evo), a hypoxia-responsive prodrug incorporating a 2-nitroimidazole group, has exhibited favorable safety profiles and demonstrated potent antitumor efficacy in clinical trials [96]. Consequently, the distinctive feature of solid tumors provides an opportune platform for the development of specific PROTAC prodrugs that can effectively target pathological tissues while sparing normal sites.

**Fig. 13:**
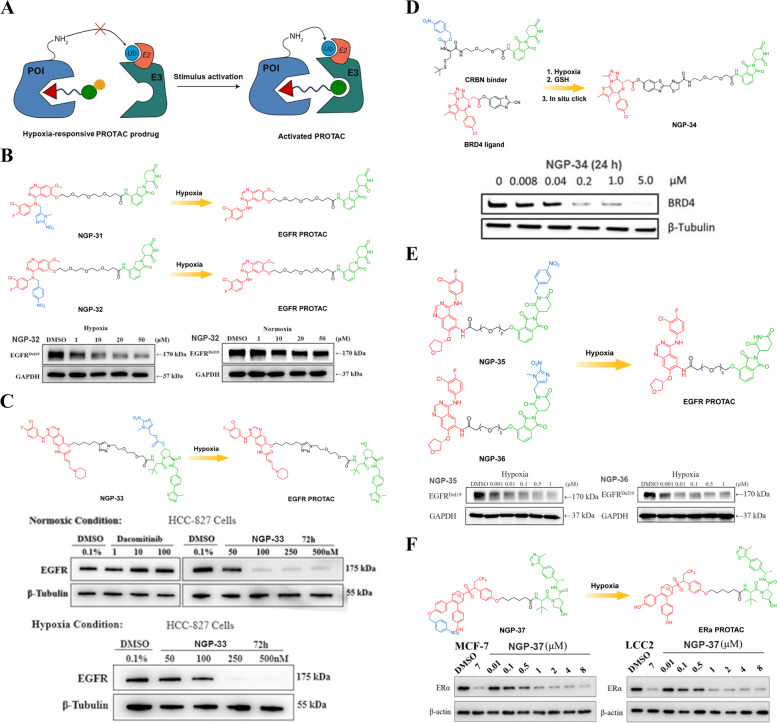
**A** Cartoon showing the structure of the hypoxia-responsive PROTAC prodrugs (adapted from [68]).** B** Chemical structures of the hypoxia-responsive PROTACs (adapted from [97]).** C** Chemical structures of the hypoxia-responsive PROTAC (adapted from [98]).** D** Chemical structures of the hypoxia-responsive PROTAC (adapted from [99]).** E** Chemical structures of the hypoxia-responsive PROTACs (adapted from [100]).** F** Chemical structures of the hypoxia-responsive PROTAC (adapted from [101])

Cheng et al*.* focused on developing hypoxia-responsive PROTACs (Fig. 13A) that specifically target the Epidermal Growth Factor Receptor (EGFR) [97]. To achieve this, they introduced hypoxia activated leaving groups (HALGs) into the 4-NH position of a gefitinib-based EGFR PROTAC, resulting in the synthesis of precursors NGP-31 (Fig. 13B) and NGP-32 (Fig. 13B). As expected, when compared to the parent compound gefitinib, both precursors showed a significant decrease in binding affinity against EGFR^Del19^. This suggests that the introduction of HALGs affected their ability to interact with the receptor. However, it is worth noting that NGP-32 exhibited a remarkable capability to induce degradation of EGFR^Del19^ in HCC4006 cells specifically under hypoxic conditions, while remaining ineffective under normoxic conditions. To further investigate the stability and release mechanism of these hypoxia-responsive PROTACs, UPLC-MS/MS analysis was conducted. The results confirmed that both NGP-31 and NGP-32 remained stable in the absence of NTR, an enzyme responsible for activating hypoxia-responsive PROTACs. However, after incubation with NTR for 20 min, active PROTAC was released from both precursors. Thus, this research presents a novel strategy for developing tumor-targeting PROTAC prodrugs by incorporating HALGs into EGFR degraders. The findings highlight the potential application of hypoxia activation as a means to selectively degrade cancer-associated proteins such as EGFR^Del19^ under specific physiological conditions like hypoxia. Further research is warranted to explore and optimize this approach for targeted cancer therapy.

Shi et al*.* proposed a hypoxia-responsive PROTAC prodrug strategy to introduce HALG technology into other POI ligands, offering a more straightforward approach for the development of novel therapeutics [98]. In their study, they successfully synthesized NGP-33 (Fig. 13C) by conjugating a VHL binding moiety and nitroimidazole caging group onto an EGFR PROTAC. The unique design of NGP-33 allowed it to specifically release the active PROTAC under hypoxic conditions, which are commonly found in solid tumors due to inadequate oxygen supply. This targeted activation resulted in dose-dependent degradation of EGFR, a key receptor involved in cancer cell growth and survival. The researchers observed robust antiproliferative activity of NGP-33 in HCC-827 cells, indicating its potential as an effective treatment option for lung cancer. Moreover, NGP-33 demonstrated good plasma stability and PK profile, suggesting its suitability for further preclinical and clinical development. To evaluate its therapeutic efficacy in vivo, the researchers conducted experiments using the HCC-827 xenograft tumor model. Remarkably, administration of NGP-33 at a dosage of 20 mg/kg every two days led to significant tumor growth inhibition (TGI) with an impressive TGI rate of 86%. These findings highlight the promising potential of NGP-33 as a novel hypoxia-activated therapy for lung cancer treatment. The successful application of HALG technology not only expands researchers' understanding of ligand-guided drug delivery systems but also provides valuable insights into developing targeted therapies that can selectively act on specific cellular environments within tumors while minimizing off-target effects on healthy tissues.

Recently, Do et al*.* made significant progress in the development of a new PROTAC approach [99]. This innovative method involves the localized formation of an active degrader, known as NGP-34 (Fig. 13D), through the orthogonal cross-linking of two molecules: BRD4 ligand and CRBN binder. Activation with GSH and NTR under hypoxic conditions enables the precise targeting and degradation of BRD4. The application of NGP-34 has shown promising results in inducing specific degradation of BRD4 protein in various cancer cell lines. This targeted degradation mechanism is particularly effective under hypoxia, making it a valuable tool for studying cancer biology and developing new therapeutic strategies. Furthermore, this novel strategy has demonstrated its efficacy not only in living cells but also in zebrafish models and mice with solid tumors. The ability to achieve hypoxia-dependent activity highlights the potential clinical relevance of hypoxia-responsive PROTACs as they can specifically target tumor cells that thrive under low oxygen conditions. One notable advantage offered by this approach is its potential to overcome some limitations associated with conventional large molecular weight PROTACs. By utilizing hypoxia for click chemistry-based conjugation reactions, hypoxia-responsive PROTACs may offer improved pharmacological properties such as enhanced stability, reduced off-target effects, and increased selectivity towards disease-related targets. These findings present an exciting avenue for the advancement of novel therapeutics targeting cancer and various other ailments.

To enhance target selectivity, Cheng et al*.* incorporated HALGs into the CRBN ligand site of EGFR^Del19^ PROTAC degrader and successfully designed and synthesized tumor hypoxia-responsive PROTACs NGP-35 (Fig. 13E) and NGP-36 (Fig. 13E) [100]. Western blot analyses revealed that NGP-35 and NGP-36 effectively and selectively degraded EGFR^Del19^ specifically in tumor hypoxia conditions. Moreover, these compounds exhibited significantly stronger inhibitory activity on cell viability in tumor hypoxia (half maximal inhibitory concentration (IC_50_) = 1.1 μM and 0.6 μM, respectively) compared to normoxic conditions (IC_50_ = 1.9 μM and 1.1 μM, respectively). Furthermore, a cell migration inhibitory assay demonstrated pronounced effects of both compounds under tumor hypoxia conditions. In addition, it was observed that under tumor hypoxia conditions, NGP-35 and NGP-36 exhibited a higher induction of cellular apoptosis compared to normoxia. The reductive activation assay provided confirmation that the active EGFR PROTAC could be released from either NGP-35 or NGP-36. However, the relatively low quantity of the active drug prompted us to consider further optimization. These findings validate the feasibility of incorporating HALGs into CRBN E3 ligands for developing hypoxia-responsive PROTACs as a means to enhance the selectivity of PROTACs.

Xie et al*.* made a groundbreaking discovery in BC therapy by developing an ERα-targeted hypoxia-responsive PROTAC NGP-37 (Fig. 13F) [101]. The innovative PROTAC NGP-37 was designed to specifically target the tumor microenvironment and enhance its safety profile. The researchers incorporated the nitrobenzene hypoxia-activating group into the ERα ligand of active PROTAC. This modification allowed for the selective activation of PROTAC under hypoxic conditions commonly found in solid tumors. By exploiting this characteristic of the tumor microenvironment, the hypoxia-responsive PROTAC NGP-37 demonstrated excellent responsiveness to low oxygen levels and effectively degraded ERα. One significant advantage of NGP-37 is its ability to mitigate cytotoxicity in normal cells. The bioactivity studies conducted by Xie et al*.* confirmed that these caged compounds possess remarkable potential for precise functional control of PROTAC drugs in BC treatment. Their ability to respond specifically to hypoxic environments not only enhances therapeutic efficacy but also minimizes off-target toxicity. This research opens up exciting possibilities for future development and optimization of targeted therapies using PROTCAs for BC treatment. Additionally, the findings from this study may pave the way for further exploration into the use of similar strategies in other types of cancer treatment, offering hope for more effective and personalized approaches to combating this disease.

#### ROS-responsive PROTAC prodrugs

In addition to inherent tumor characteristics such as hypoxia, solid tumors are characterized by an elevated level of ROS. The ROS present in the tumor microenvironment typically encompass superoxide, hydroxyl radical, and hydrogen peroxide (H_2_O_2_) [102, 103]. Tumor cells demonstrate significantly elevated levels of H_2_O_2_ up to 100 μmol/L compared to normal cells [104]. Over the years, extensive efforts have been made to exploit the heightened ROS level in solid tumors for the development of ROS activatable drug delivery systems or prodrugs aimed at enhancing cancer therapy. The development and utilization of ROS activatable prodrugs and targeted drug delivery systems possess immense potential in enhancing the efficacy of cancer therapy. They offer a more precise approach towards eradicating malignant cells while reducing systemic toxicity associated with conventional chemotherapy drugs. Understanding and leveraging the elevated levels of ROS present in solid tumors provide valuable insights into novel strategies for combating cancer effectively. Continued research efforts focused on harnessing this unique characteristic will undoubtedly contribute significantly towards advancing personalized medicine approaches for improved patient outcomes in oncology treatments.

**Fig. 14:**
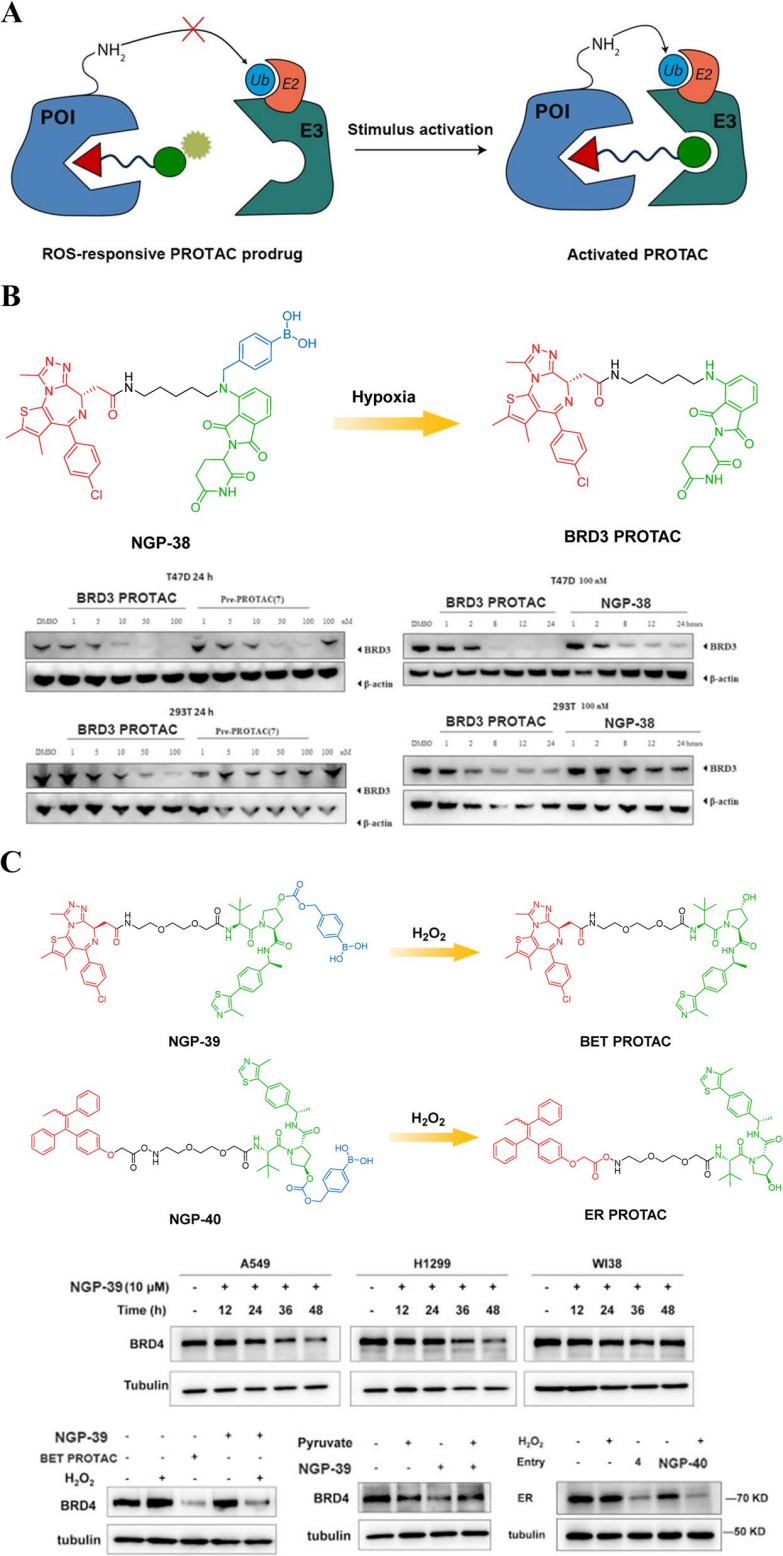
**A** Cartoon showing the structure of the ROS-responsive PROTAC prodrug (adapted from [68]).** B** Chemical structures of the ROS-responsive PROTAC prodrug (adapted from [105]).** C** Chemical structures of the ROS-responsive PROTAC prodrugs (adapted from [106])

For example, Liu et al*.* initially introduced a ROS-responsive PROTAC (Fig. 14A) for the degradation of tumor-specific proteins [105]. NGP-38 (Fig. 14B) was synthesized through a process of grafting an arylboronic acid onto the amine or glutarimide of CRBN ligand of PROTAC. This innovative approach allows for targeted protein degradation within cells. To restore the PROTAC molecule, the boronic acid group is cleaved from the NGP-38 using H_2_O_2_. Comparative studies were conducted between the parent PROTAC and the designed NGP-38 to evaluate their efficacy in degrading target proteins. The results showed that while both compounds effectively degraded target proteins, there was a notable difference in their responsiveness to ROS. The parent PROTAC exhibited no response to ROS, whereas the designed NGP-38 demonstrated sensitivity. Further investigations were carried out using 293 T human embryonic kidney cells and T47D tumor cells as experimental models. It was observed that both cell types experienced comparable degradation of target proteins when treated with the parent PROTAC. However, when exposed to the designed NGP-38, specifically targeting BRD3 protein, T47D BC cells displayed dose-dependent and time-dependent degradation patterns. These findings suggest that this newly developed NGP-38 has great potential for tumor-targeted protein degradation. By selectively degrading specific proteins involved in cancer progression, it may offer a promising therapeutic strategy for treating BC and potentially other types of tumors as well. Further research is imperative to delve into its complete potential and enhance its efficacy in clinical applications.

Recently, the development of ROS-responsive PROTAC precursors NGP-39 (Fig. 14C) and NGP-40 (Fig. 14C) by Yu et al*.* has opened up a new avenue for targeted protein degradation in cancer cells [106]. These precursors can be activated by endogenous H_2_O_2_ in cancer cells to release active PROTACs (BET PROTAC and ER PROTAC), which effectively degrade targeted proteins while leaving normal cells almost unaffected. The higher BRD4 degradation activity and cytotoxicity of NGP-39 towards cancer cells is mainly due to the higher endogenous concentration of H_2_O_2_ in these cells, as characterized by the H_2_O_2_-responsive fluorescence probe. This method has been validated through Western blot assays and cytotoxicity experiments that demonstrate its effectiveness in degrading BRD4 more effectively and being more cytotoxic in H_2_O_2_-rich cancer cells than in H_2_O_2_-deficient normal ones. Moreover, this strategy has also been extended to ER-PROTAC precursor NGP-40, showing its ability to induce ER degradation dependent on the presence of H_2_O_2_. Thus, this new approach offers a promising way for inducing targeted protein degradation specifically within cancerous tissues without affecting healthy ones. This research represents an important step forward towards developing effective therapies for treating cancers with minimal side effects on healthy tissues. The use of endogenous molecules such as hydrogen peroxide offers a unique advantage over traditional methods that rely on exogenously administered drugs or chemicals.

## Biomacromolecule-PROTAC conjugates

### Antibody-PROTAC conjugates

**Fig. 15:**
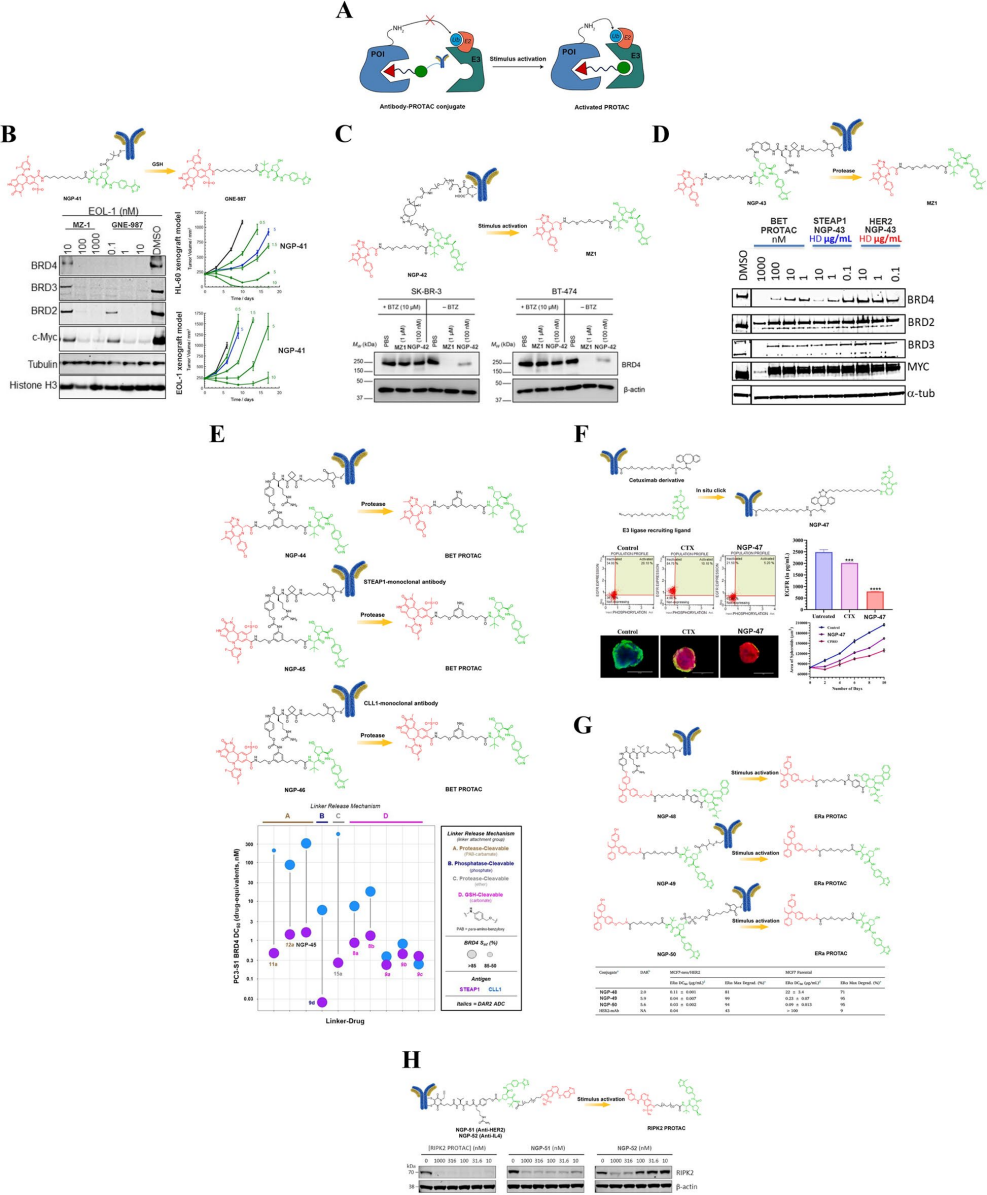
**A** Cartoon showing the structure of the antibody-PROTAC conjugates (adapted from [68]).** B** Chemical structures of the antibody-PROTAC conjugate (adapted from [112]). **C** Chemical structures of the antibody-PROTAC conjugate (adapted from [113]). **D** Chemical structures of the antibody-PROTAC conjugate (adapted from [114]). **E** Chemical structures of the antibody-PROTAC conjugates (adapted from [115]).** F** Chemical structures of the antibody-PROTAC conjugate (adapted from [116]). **G** Chemical structures of the antibody-PROTAC conjugates (adapted from [117]). **H** Chemical structures of the antibody-PROTAC conjugates (adapted from [118])

ADCs have become a highly promising category of anticancer drugs due to their ability to selectively deliver cytotoxic agents to malignant cells while sparing healthy tissues [107–109]. The success of trastuzumab deruxtecan (DS-8201), which was recently approved by the FDA for the treatment of various cancer types, has further fueled interest in this field [110, 111]. Conventional ADCs are composed of an antibody that targets a specific antigen on the surface of cancer cells, a cytotoxic payload that kills the cell upon internalization, and a chemical linker that connects them. This innovative approach enables precise administration of highly potent cytotoxic medications directly to malignant cells, leading to enhanced effectiveness and diminished toxicity in contrast to conventional chemotherapy. In addition to their potent antitumor activity, ADCs also exhibit desirable PK properties similar to those of monoclonal antibodies. They are stable in circulation and can be engineered for prolonged T_1/2_ and improved tissue penetration. Inspired by the success of ADCs, drug scientists have begun exploring new ways to harness the advantages of antibodies for other therapeutic modalities. One such approach is antibody-PROTAC conjugates (Fig. 15A), which combine the specificity and selectivity of antibodies with the ability to induce protein degradation through targeted recruitment of E3 ubiquitin ligases. These developments highlight the growing importance and potential impact of antibody-based therapeutics in oncology research and beyond.

To address the challenges posed by the well-established GNE-987 [119], which is a very potent BRD degrader but exhibits off-tumor toxicity in vivo, Pillow et al*.* have undertaken innovative measures [112]. They successfully designed and synthesized the first BRD antibody-PROTAC conjugate NGP-41, as depicted in Fig. 15B, with a drug-to-antibody ratio (DAR) of 6. This breakthrough was achieved by conjugating the C-type lectin-like molecule-1 (CLL1)-lead antibody to BRD degrader employing a novel disulfide-bonded linker. Remarkably, the success of the novel linker tailored for NGP-41 is evident in its stability over a period of seven days. This extended stability ensures that the NGP-41 compound remains intact and active, allowing for effective targeting and degradation of pathogenesis-related POIs. Furthermore, in vivo studies conducted on mice after a single intravenous injection revealed a PK profile with a T_1/2_ exceeding 14 days. The significance of NGP-41 lies not only in its stability and PK profile but also in its remarkable efficacy against tumors. Multiple xenograft studies have consistently shown tumor regression when treated with NGP-41, highlighting its potential as an effective therapeutic agent. Importantly, this tumor regression was found to be both antigen-dependent and dose-dependent, further emphasizing the specificity and potency of NGP-41. In contrast to the impressive activity exhibited by NGP-41, the parent PROTAC did not demonstrate any significant effects. This stark difference underscores the importance of optimizing PROTAC degrader candidates into potent bifunctional compounds like antibody-PROTAC conjugates. The challenges associated with undesirable physicochemical properties and PK profiles of non-conjugated degraders can be overcome by converting inadequate PROTAC degraders into stable and efficacious molecules like antibody-PROTAC conjugates.

Next, Maneiro et al*.* designed and synthesized a new antibody-PROTAC conjugate (NGP-42, DAR = 4, Fig. 15C), which targets BRD4 extremely well by utilising an unbreakable linker [113]. NGP-42 has an important function in specifically targeting the HER2/neu receptor on tumor cells and selectively degrading BRD4 in those cells, particularly in the BT-474 and SK-BR-3 cell lines. This occurs without affecting the levels of BRD4 in normal cells or in the MCF-7 and MDA-MB-231 cell lines. NGP-42 was designed not only for its specific therapeutic benefits, but also to serve as a labeled antibody-PROTAC conjugate derivative that can be used to observe the transport and internalization processes in living cells through confocal microscopy by utilizing fluorescence. This allowed researchers to track the intracellular fate of NGP-42 and gain insights into its mechanism of action at a cellular level. The development of NGP-42 represents a promising advancement in targeted cancer therapy, offering both specific recognition of tumor cell surface receptors and selective degradation of oncogenic proteins within those cells. The ability to monitor its intracellular trafficking further enhances the understanding of how this novel antibody–drug conjugate functions within the complex microenvironment of tumors.

The Surface Antigen Prostate-6-Transmembrane Epithelial Antigen-1 (STEAP-1) has been identified as a crucial marker for the diagnosis and treatment of prostate cancer. It is often overexpressed in patients with this disease, making it an attractive target for therapeutic intervention. Recently, Dragovich et al*.* developed an antibody-PROTAC conjugate, NGP-43 (Fig. 15D), which was designed to degrade STEAP1 protein levels within cells [114]. This unique strategy allowed NGP-43 to achieve a high DAR (DAR = 6). Interestingly, the study's results suggested that a high DAR may be necessary to achieve effective protein degradation. Specifically, NGP-43 demonstrated better intracellular BRD4 degradation than other antibody-PROTAC conjugates did. However, despite these promising findings, the antiproliferative effect of NGP-43 on prostate cancer PC3 cells with elevated levels of STEAP-1 in vitro was not evident. Overall, while NGP-43 represents an innovative approach towards targeting STEAP1 in prostate cancer therapy, further research is needed to determine its efficacy in vivo and potential clinical applications.

Dragovich et al*.* conducted structural modifications on MZ1, focusing on hybridisation of amine molecular functional groups with BET-binding molecules, PROTAC linkers, or VHL ligands. These chemical treatments were identified as promising targets for modification [115]. By introducing amide or carbamate linkages, they successfully linked the linker moieties to the MZ1 derivatives efficiently. One notable antibody-PROTAC conjugate that was constructed is NGP-44 (Fig. 15E), which uses an amine PROTAC linker with a DAR of 6. This particular antibody-PROTAC conjugate demonstrated enhanced degradation of BRD4 compared to another antibody-PROTAC conjugate (DAR = 2). The results highlight the importance of achieving necessary degrader loading in order to enable effective protein degradation through antibody-PROTAC conjugate-mediated mechanisms. Dragovich et al*.* designed and developed NGP-45 (DAR = 6, Fig. 15E), in which MZ1 was replaced by the BET-binding molecule GNE-987, which exerted an effective antiproliferative effect on PC3-S1 cells and significantly increased the degradation activity of BRD4. Moreover, in in vivo experiments, NGP-45 exhibited TGI that was dose- and antigen-dependent. Additionally, when conjugated with the CLL1-monoclonal antibody, the same parent drug resulted in robust antigen-dependent anti-tumor effects in in vivo experiments in mice, as demonstrated by NGP-46 (DAR = 6, Fig. 15E). The above experimental results showed the utilization of this specific antibody-PROTAC conjugate holds substantial potential for treating diverse forms of tumors.

With the continued development of PROTAC technology, Vartak et al*.* have made significant progress in developing a novel approach to target and degrade the EGFR. Their study aimed to develop a potent antibody-PROTAC conjugate NGP-47 (Fig. 15F) for the treatment of non-small cell lung cancer (NSCLC) [116]. To achieve this, they utilized a combination of lysine conjugation and azide-alkyne cyclization click chemistry techniques to bind together two important molecules—cetuximab, an antibody targeting EGFR, and pomalidomide, a small molecule with proteasome inhibitory activity. This innovative combinatorial approach allowed for the creation of NGP-47, which effectively degraded EGFR. The researchers then evaluated the efficacy of NGP-47 by comparing its IC_50_ values with those of cetuximab in both EGFR-resistant H1650 cells and EGFR-sensitive HCC827 cells. The results showed that NGP-47 exhibited significantly lower IC_50_ values compared to cetuximab alone, indicating its enhanced potency in inhibiting EGFR-mediated signaling pathways. Furthermore, the team conducted multicellular 3D spheroid assays to assess the impact of NGP-47 on cell proliferation. The results demonstrated that NGP-47 not only significantly inhibited cell growth but also induced apoptosis when compared to both antibody treatment alone and control groups. This study represents an important advancement in PROTAC research by successfully developing a novel combinatorial approach for degrading EGFR using an antibody-PROTAC conjugate. The promising results obtained from cellular assays suggest that further exploration is warranted towards utilizing such strategies as potential therapeutic interventions against NSCLC or other cancers driven by aberrant protein expression or activation.

Dragovich et al*.* conducted a comprehensive investigation on various antibody-PROTAC conjugate degraders that specifically target the ERα protein [117]. Among these, they successfully developed NGP-48 (DAR = 2, Fig. 15G), which is a PROTAC designed to recruit XIAP in order to target ERα. NGP-48 efficiently released active PROTAC intracellularly through protease-mediated cleavage of the Val-Cit-PAB linker. The researchers observed that NGP-48 exhibited remarkable antigen-triggered degradation capability when tested against HER2/MCF7-neo cells in vitro. However, when tested in vivo, it unexpectedly showed insufficient PK properties. This limitation was likely attributed to biotransformation occurring at the XIAP binding site. To overcome this challenge and enhance the PK profile of their protein degraders, Dragovich et al*.* developed two additional antibody-PROTAC conjugates derived from VHL-based PROTACs. These new compounds aimed to improve stability and optimize drug-like properties for potential therapeutic applications. NGP-49 (DAR = 6, Fig. 15G) and NGP-50 (DAR = 6, Fig. 15G) were synthesized by incorporating a disulphide-containing or phosphatase-cleavable linker onto the parent PROTAC, respectively. Both antibody-PROTAC conjugates exhibited ERα degradation in an antigen-dependent manner and demonstrated satisfactory in vivo stability. In conclusion, this study expands the scope of antibody-PROTAC conjugate technology for efficient delivery of ER PROTACs to tumor cells, emphasizing the crucial roles played by both ADC linker technology and parent PROTAC properties in the preparation of antibody-PROTAC conjugates.

Although antibody-PROTAC conjugates have shown promising results in selectively degrading BRD4, EGFR, and ER within cells, there are still several important aspects that need further exploration. One such aspect is the targeting of linking chemistries through antibody conjugation. The choice of linker chemistry plays a crucial role in determining the stability and efficacy of the conjugate. Different linkers may exhibit varying degrees of stability or release rates, which can impact the overall effectiveness of targeted protein degradation. In 2023, Chan et al*.* conducted a study that further advanced the application of antibody-PROTAC conjugates by demonstrating their ability to selectively degrade the Receptor-Interacting Protein Kinase 2 (RIPK2) in HER2 + cell lines [118]. They developed a novel antibody-PROTAC conjugate called NGP-51, which consisted of a protease-hydrolysable linker connecting the antibody and the degrader. Through their experiments, Chan et al*.* observed successful degradation of RIPK2 in HER2 + cell lines when treated with NGP-51 (DAR = 4, Fig. 15H). Interestingly, they also tested an equivalent anti-IL4 antibody-PROTAC conjugate called NGP-52 (DAR = 3.7, Fig. 15H) but found no degradation at treatment-relevant concentrations. This highlights the specificity and selectivity of antibody-PROTAC conjugates in targeting particular proteins for degradation. Importantly, neither NGP-51 nor NGP-52 showed any significant effect on RIPK2 levels in HER2- cell lines. This suggests that these bioconjugates have the potential for cell-selective delivery, as they only induce protein degradation in cells expressing high levels of specific characteristic proteins like HER2. The results of this research offer invaluable perspectives into the prospective utilization of PROTAC-based therapies for precise protein degradation. By utilizing antibodies as carriers for PROTACs, researchers can achieve selective delivery to specific cell types based on surface protein expression profiles. This opens up new possibilities for developing personalized treatments tailored to individual patients or diseases characterized by distinct molecular markers.

### Aptamer-PROTAC conjugates

**Fig. 16:**
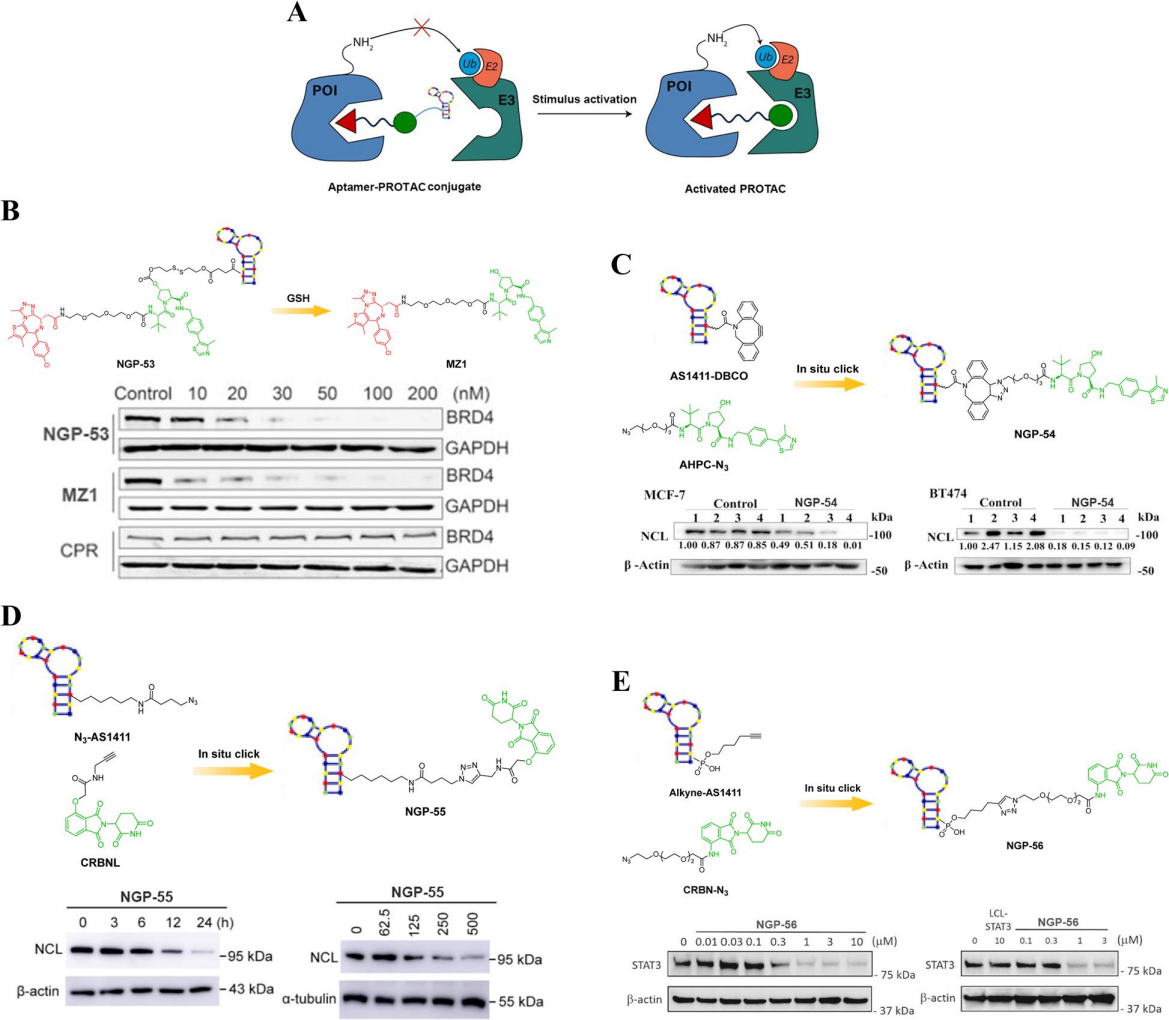
**A** Cartoon showing the structure of the aptamer-PROTAC conjugates (adapted from [68]).** B** Chemical structures of the antibody-PROTAC conjugate (adapted from [123]).** C** Chemical structures of the antibody-PROTAC conjugate (adapted from [124]).** D** Chemical structures of the antibody-PROTAC conjugate (adapted from [125]). **E** Chemical structures of the antibody-PROTAC conjugate (adapted from [126])

Aptamers, as single-stranded oligonucleotide sequences designed to selectively bind with target proteins, have gained significant attention in the field of biomedical research [120]. These "chemical antibodies" offer several advantages over traditional antibodies, including low immunogenicity and high tissue penetration. Researchers have explored the potential of using aptamer-drug conjugates based on AS1411 (the most promising aptamer) for targeted delivery of anticancer drugs such as doxorubicin [121, 122]. By attaching the aptamer to these drugs, researchers aim to enhance their tumor-specific delivery and improve their efficacy against cancer cells. Furthermore, there is growing interest in utilizing aptamers for enhancing the antitumor efficacy and tumor-targeting ability of PROTACs. The conjugation of an aptamer with a PROTAC molecule (Fig. 16A) may provide a dual targeting approach by specifically recognizing cancer cell surface markers through the aptamer while simultaneously promoting protein degradation through PROTAC action. These advancements highlight the potential of using aptamers not only as therapeutic agents themselves but also as tools for improving targeted drug delivery strategies in cancer therapy. Continued exploration and innovation in this domain offer immense potential for propelling the frontiers of precision medicine methodologies and enhancing patient outcomes within the realm of oncology.

The development of the first aptamer-PROTAC conjugate NGP-53 (Fig. 16B) by He et al*.* represents a significant advancement in targeted cancer therapy [123]. By linking MZ1 and AS1411 through an ester-disulfide linkage, NGP-53 is able to selectively target nucleolin (NCL)-rich tumor cells while avoiding healthy cells. This is achieved through the preferential breaking of the disulfide link by endogenous GSH upon import into tumor cells. In addition to its selectivity, NGP-53 has also demonstrated promising anti-tumor effects without significant adverse effects in preclinical studies using the MCF-7 xenograft model. The effective degradation of BRD4 in NCL + MCF-7 cells further highlights the potential therapeutic benefits of this novel approach. While promising, further research is required to enhance the strategy's specificity for tumor tissue and its therapeutic efficacy. Nevertheless, aptamer-PROTAC conjugation offers a new avenue for developing targeted cancer therapies that may overcome some of the limitations associated with traditional approaches. With continued advancements in this field, we may see even greater success in treating various types of cancers with minimal side effects on healthy tissues.

By employing the aptamer AS1411 as a specific binding agent for the nucleosome protein NCL and linking it to the VHL E3 ligand, Zhang et al*.* have made progress in the development of aptamer-PROTAC conjugates [124]. In their study, they successfully designed and synthesized a novel aptamer-PROTAC conjugate called NGP-54 (Fig. 16C). One of the key advantages of NGP-54 is its excellent serum stability and water solubility, which are crucial for its potential therapeutic applications. They found that NGP-54 specifically attaches to and internalizes into BC cells but not normal cells. This selective targeting is attributed to the varying expression levels of NCL on the surface of these cells. By exploiting this differential expression pattern, NGP-54 holds great promise as a specific therapeutic agent for BC treatment. Furthermore, they demonstrated that NGP-54 effectively induces the degradation of NCL both in vitro and in vivo. This degradation process was shown to be mediated by the formation of a VHL-NGP-54-NCL ternary complex within BC cells. The disruption of NCL function through targeted degradation has been proven to inhibit BC cell proliferation and migration. This groundbreaking work highlights the potential use of aptamers in designing specific PROTACs with high selectivity towards target proteins like NCL. Aptamers offer several advantages over traditional small-molecule compounds, including their ability to bind targets with high specificity and affinity while being easily modified for different applications.

Chen et al*.* also reported a unique approach, utilizing aptamers as targeting warheads, to induce degradation of non-druggable proteins [125]. To demonstrate the proof of concept, the researchers developed several aptamer-PROTAC conjugates specifically tailored for the degradation of a carcinogen known as NCL. Among these compounds, NGP-55 (Fig. 16D) showed remarkable efficacy in degrading the NCL protein through a ubiquitin proteasome-dependent mechanism. Furthermore, it was noted that NGP-55 demonstrated effective inhibition of NCL-mediated proliferation in breast cancer cells. Building upon this success, the scientists went on to develop a photo-controllable version of NGP-55 called opto-NGP-55. By introducing a photo easily digestible oligonucleotide into the structure of NGP-55, NGP-55 was able to reduce potential on-target toxicity. Upon UVA irradiation, opto-NGP-55 underwent cleavage and released active NGP-55 molecules. This activation led to efficient degradation of NCL and further validated the versatility and controllability of aptamer-PROTAC conjugates. These exciting results highlight that aptamer-PROTAC conjugates represent an innovative and feasible approach for selectively degrading proteins of interest. The ability to utilize aptamers as targeting warheads provides flexibility in targeting non-druggable proteins that traditional small molecule inhibitors cannot effectively address. With further advancements in this field, PROTAC-based strategies may hold great promise for therapeutic interventions against various diseases where specific protein targets need to be degraded or modulated for desired outcomes.

Lately, Shih et al*.* also effectively induced the degradation of STAT3 using a novel aptamer-PROTAC conjugate technology [126]. Their study demonstrated that the decoy aptamer, which was specifically engineered for STAT3, exhibited a high affinity for binding to the E3 conjugate. This interaction resulted in the formation of potent PROTACs, which were able to selectively recruit different E3 ubiquitin ligases. It is worth noting that the efficacy of NGP-56 (Fig. 16E) in promoting STAT3 degradation is a significant finding in the field of targeted protein degradation. The specific mechanism by which NGP-56 recruits CRBN as its E3 ubiquitin ligase partner sheds light on the potential for developing novel therapeutic strategies for diseases associated with dysregulated STAT3 signaling. Furthermore, the importance of the aptamer sequence in facilitating STAT3 degradation cannot be overstated. By disrupting this sequence and introducing a decoy aptamer targeting STAT3, the effectiveness of NGP-56 was significantly diminished. Moreover, NGP-56-induced STAT3 degradation was inhibited by bortezomib, thalidomide, siRNA, and MLN7243-mediated CRBN deletion. The findings from this study provide valuable insights into the mechanism of STAT3 degradation and its potential as a target for cancer therapy. The identification of CRBN as the responsible E3 ubiquitin ligase sheds light on the specific pathway through which STAT3 is degraded, offering a potential avenue for developing targeted therapies. Furthermore, the observed reduction in NCI-H2087 cell survival after treatment with NGP-56 highlights the potential of this compound as a cytocidal agent for cancer cells that rely heavily on STAT3 signaling. This suggests that targeting STAT3 degradation could be an effective strategy for combating cancers driven by aberrant STAT3 activity. The use of decoy inducer-PROTAC conjugates to induce protein degradation against oncogenic transcription factors such as STAT3 represents an innovative approach to cancer therapy. By specifically targeting these key regulators of tumor cell survival and growth, this strategy holds promise for the development of novel anti-cancer therapies with potentially fewer off-target effects compared to traditional treatments. Overall, these discoveries underscore the therapeutic potential of targeting protein degradation pathways in cancer cells and pave the way for further research into exploiting this approach for developing more effective and selective anti-cancer treatments.

## Nano-PROTAC polymers

**Fig. 17:**
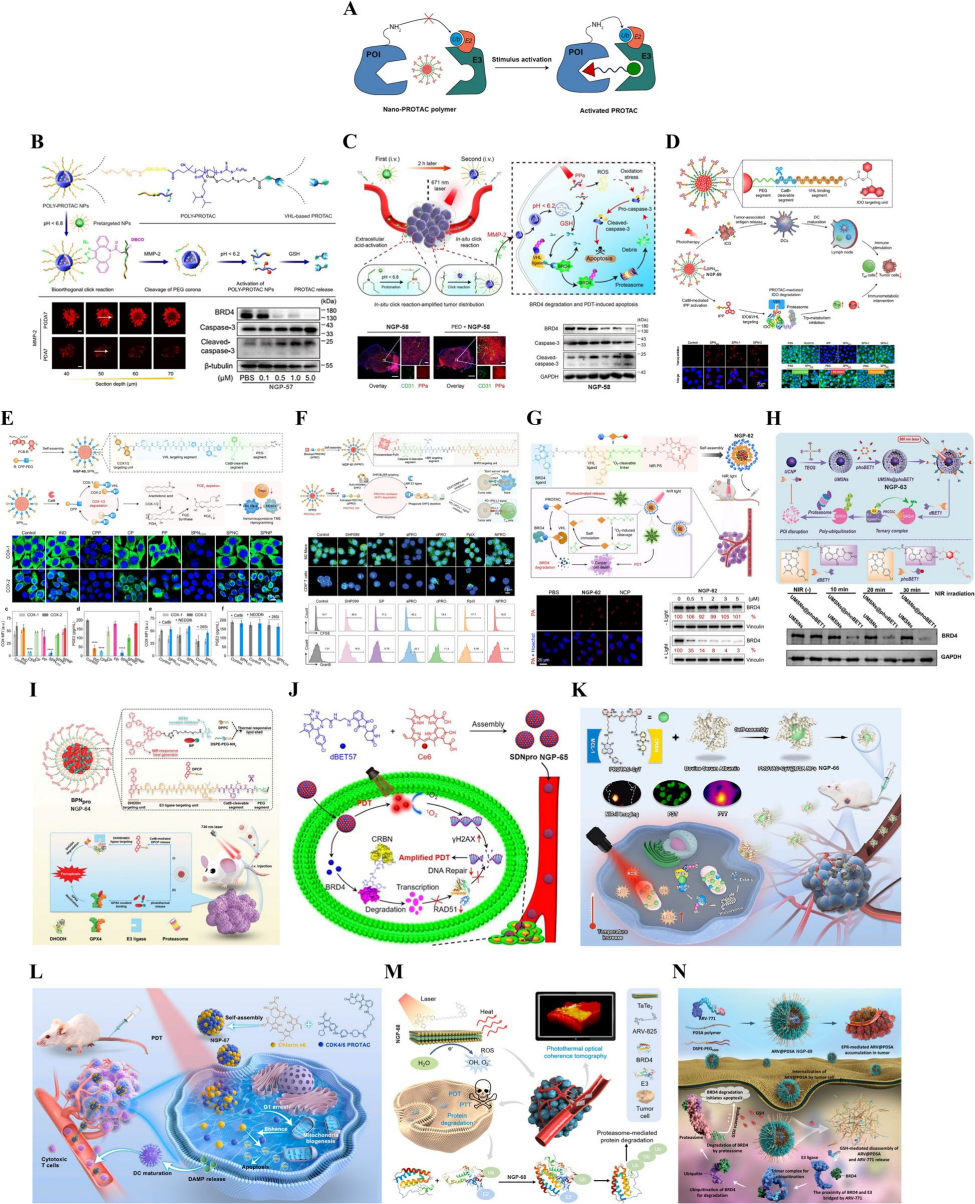
**A** Cartoon showing the structure of the nano-PROTAC polymers (adapted from [68]).** B** Chemical structures of the structure of the nano-PROTACs (adapted from [135]).** C** Chemical structures of the structure of the nano-PROTACs (adapted from [136, 137]).** D** Chemical structures of the structure of the nano-PROTACs (adapted from [138]). **E** Chemical structures of the structure of the nano-PROTACs (adapted from [139]). **F** Chemical structures of the structure of the nano-PROTACs (adapted from [140]).** G** Chemical structures of the structure of the nano-PROTACs (adapted from [141]).** H** Chemical structures of the structure of the nano-PROTACs (adapted from [142]).** I** Chemical structures of the structure of the nano-PROTACs (adapted from [143]).** J** Chemical structures of the structure of the nano-PROTACs (adapted from [144]).** K** Chemical structures of the structure of the nano-PROTACs (adapted from [145]).** L** Chemical structures of the structure of the nano-PROTACs (adapted from [146]). **M** Chemical structures of the structure of the nano-PROTACs (adapted from [147]). **N** Chemical structures of the structure of the nano-PROTACs (adapted from [148])

Nanosized drug delivery systems (NDDS) have emerged as a promising approach for cancer therapy, offering numerous advantages over traditional small molecular drugs. NDDS have been extensively studied and exploited due to their ability to improve the PK profiles of therapeutic agents [127–132]. Recently, researchers have turned their attention towards investigating the engagement between NDDS and PROTAC prodrugs. By combining NDDS with PROTAC prodrugs, several benefits can be achieved. Firstly, by encapsulating PROTACs within nanocarriers, their blood circulation time can be significantly extended compared to free-form counterparts. This allows for sustained release of active compounds at tumor sites over an extended period. Secondly, utilizing NDDS can enhance tumor distribution by facilitating passive targeting via the enhanced permeability and retention (EPR) effect. Last but importantly, cellular uptake of PROTACs can also be facilitated by employing NDDS strategies. Nanoparticles possess unique characteristics that promote efficient internalization into cells through various mechanisms such as endocytosis or receptor-mediated pathways. Exploring the interaction between NDDS and PROTAC prodrugs holds immense promise in enhancing therapeutic performance against cancerous diseases (Fig. 17A). Through increasing tumor distribution efficiency, elongating blood circulation time, and facilitating cellular uptake, the combination could potentially revolutionize cancer treatment strategies by maximizing efficacy while minimizing side effects on healthy tissues [133, 134].

Gao et al*.* introduced a groundbreaking concept in their research by developing nano-PROTAC polymer NGP-57 (Fig. 17B) [135]. These innovative polymers consist of an amphiphilic structure, with a hydrophilic PEG segment and a hydrophobic segment conjugated with PROTAC molecules. This unique design allows the nano-PROTAC to self-assemble into micellar nanoparticles. The presence of the hydrophilic PEG segment on the surface of the nanoparticles enhances their stability in the bloodstream, preventing rapid clearance by immune cells. As a result, NGP-57 can circulate for extended periods, increasing their chances of reaching tumor tissues. Furthermore, NGP-57 exhibits enhanced accumulation at tumor sites through an EPR effect. Once accumulated at the tumor site, these nano-PROTAC nanoparticles are designed to release active PROTAC molecules for precise degradation of POIs. They chose ARV-771-based PROTAC targeting BRD4 protein as their model PROTAC molecule. By grafting it onto the polymer backbone through a disulfide chain spacer, they ensured controlled release at specific locations. They also incorporated sheddable PEG corona into these nano-PROTAC particles to further enhance their functionality. Activation and dissociation mechanisms were developed using intracellular acidic conditions and extracellular matrix metalloproteinase-2 (MMP-2) respectively. MMP-2 activation triggers nanoparticle dissociation outside cells while GSH-triggered cleavage inside cells releases active PROTAC molecules from the disulfide bond spacer. In vivo experiments demonstrated that NGP-57 effectively degraded BRD4 protein within tumor tissues and exhibited potent inhibition against MDA-MB231 tumor growth models. Their study showcases how nanotechnology can be harnessed to improve targeted therapy strategies, offering promising prospects for precision medicine approaches in cancer treatment.

To further enhance the accumulation of nano-PROTAC nanoparticles specifically in tumor cells, researchers developed a bioorthogonal nano-PROTAC known as N3@nano-PROTAC (NGP-58, Fig. 17C) [136, 137]. This innovative approach involves modifying the azide group on the PEG head of the nanoparticle. The dibenzocyclooctyne (DBCO) groups, which possess high reactivity towards azide-containing molecules, were efficiently delivered to the tumor mass using a sophisticated system of nanoparticles that respond to the extracellular acidity of tumors. The encapsulation of DBCO groups within the hydrophobic core of the pre-targeted nanoparticle allows for their protection in normal conditions. However, when exposure to the acidic tumor microenvironment, these DBCO groups become exposed and ready for further reaction. This unique feature enables the specific trapping of azide group-modified nano-PROTAC nanoparticles in tumor tissue through a click reaction with the pre-delivered DBCO groups. By employing this bioorthogonal strategy, there is a significant increase of fourfold greater tumor localization of PROTAC compared to using free PROTAC alone as a control. Moreover, it is worth noting that the NGP-58 has been further modified by grafting PPa photosensitizer onto its polymer backbone. This addition endows the nanoparticle with PDT properties, which can enhance apoptosis in tumor cells upon activation by light. In vivo antitumor study suggested that combining PDT and BRD4 degradation resulted in highly efficient induction of tumor cell apoptosis and inhibition of tumor growth. The synergistic effect between these two mechanisms provides a promising approach for combating cancer and potentially overcoming drug resistance commonly observed with single-agent therapies. This research showcases a novel approach utilizing targeted nanoparticles loaded with both PROTACs and PDT agents to achieve improved therapeutic outcomes against tumors.

Zhang et al*.* engineered a semiconducting nano-PROTAC polymer NGP-59 (namely SPNpro, Fig. 17D) by integrating its properties of activatable protein degradation activity and photo-immunometabolic efficacy [138]. NGP-59 is composed of two parts, a PEG shell that connects to the peptide-derived PROTAC, and a semiconducting polymer with PDT efficacy that serves as the core via a chelator protein B (CatB) cleavage peptide. CatB was utilized as the stimulus for liberating PROTAC from NGP-59 due to its status as a cancer biomarker that is commonly overexpressed in various tumor cells, melanoma, encompassing prostate cancer, and BC [149]. The semiconducting polymer component of NGP-59 exhibited PDT efficacy at the tumor site when subjected to laser irradiation, thereby directly inducing immunogenic cell death (ICD) and eliminating tumor cells in these malignant entities. At the same time, the peptide-derived PROTAC was retrieved from NGP-59 at the tumor site via CatB, resulting in the degradation of the immunosuppressive enzyme indoleamine 2,3-dioxygenase (IDO). In a mutually reinforcing manner, PDT-induced immunogenic cell death (ICD) enhanced the immunogenicity of the tumor while simultaneously relieving immunosuppression through IDO degradation. Immunofluorescence assays demonstrated the ability of NGP-59 nanoparticles and peptide-derived PROTAC to degrade IDO via the ubiquitin–proteasome pathway. Significant reduction in IDO levels was observed in both NGP-59 and IPP groups; however, groups lacking either the VHL ligand or target unit exhibited minimal changes in IDO expression. Moreover, co-administration of inhibitors effectively prevented IDO degradation. As a result, the localized immunometabolic intervention mediated by NGP-59, which combines immune stimulation with activatable protein degradation through photo-immune therapy, effectively suppressed tumor growth in a mouse model. In addition to targeting IDO protein, this innovative protein hydrolysis technology can also be utilized to uniquely degrade other immunosuppressive proteins associated with tumors, thereby enhancing cancer therapy by seamlessly linking corresponding moieties endowed with exceptional targeting capabilities.

Given the remarkable agility exhibited by nano-PROTAC polymers, it becomes conceivable to effortlessly manipulate alternative target proteins through the modification of peptide-derived PROTACs' targeted units. Thus, Zhang et al*.* developed a novel nano-PROTAC (SPN_COX_, NGP-60, Fig. 17E) comprising of a peptide-derived PROTAC targeting Cyclooxygenase 1/2 (COX-1/2), a semiconducting polymer core, and a CatB-cleaved peptide as the linker [139]. The selection of COX-1/2 as the target was based on its metabolite prostaglandin E2 (PGE2) which has been shown to exert beneficial effects on immune suppressor cells including M2-type macrophages (M2 Macs), myeloid-derived suppressor cells (MDSCs), and regulatory T cells (Tregs). Consequently, by eliminating COX-1/2 and reducing the accumulation of PGE2, this approach holds promise for reprogramming the immunosuppressive tumor microenvironment and downregulating immune suppressor cells. Hence, the reprogramming of the suppressive tumor microenvironment would be achieved through the elimination of COX-1/2 enzymes, thereby mitigating the accumulation of their metabolite (PGE2) and subsequently attenuating immune suppressor cells. In the meantime, the utilization of semiconducting polymer core-mediated PDT not only enables direct elimination of tumor cells but also induces ICD, thereby eliciting an immune response. The author convincingly demonstrates that NGP-60 effectively regresses tumor growth and prevents recurrence through a synergistic approach involving photo-metabolic cancer immunotherapy via PDT and activatable degradation of COX-1/2. This groundbreaking study represents the pioneering development of tumor microenvironment-reprogramming PROTACs, which holds immense potential for advancing cancer immunotherapy applications.

Zhang et al*.* conducted a new study where they demonstrated the effectiveness of a novel nano-PROTAC NGP-61 (NPRO, Fig. 17F) for degrading immune checkpoints [140]. NGP-61 consisted of two components: a photosensitizer called protoporphyrin IX (PpIX) and a PROTAC peptide that targeted Src Homology 2 Domain-Containing Phosphatase 2 (SHP2), which was cleaved by caspase 3. They found that NGP-61 was able to induce ICD through PDT. Additionally, they observed an upregulation of caspase 3 expression in tumor cells, which further promoted the activation of the peptide-derived PROTAC in macrophages and CD8 + T cells. This activation led to the depletion of SHP2, an important protein involved in immunosuppressive checkpoint signaling pathways such as CD47/SIRPa and programmed cell death-1/programmed cell death ligand 1 (PD-1/PD-L1). By blocking these immunosuppressive checkpoint signaling pathways, the degradation of SHP2 reinvigorated antitumor macrophages and T cells. This combination approach of protein degradation-based modulation of checkpoint signals and PDT holds great potential for cancer immunotherapy. They have made significant progress in developing a comprehensive PROTAC platform for cancer immunotherapy. Their findings provide valuable insights into how nanotechnology can be utilized to enhance immune responses against tumors by targeting specific proteins involved in immune checkpoint regulation.

In order to confine the protein degradation activity of PROTACs to cancer lesions, Wang et al*.* developed a novel approach by preparing nano-PROTAC, known as NAP (NGP-62, Fig. 17G) [141]. NGP-62 was created through the self-assembly of an amphiphilic conjugate of PROTAC linked with near-infrared (NIR) photosensitizer (PS) using a self-immolative thioketal linker. The nanoformulation of NGP-62 significantly enhanced the accumulation of PROTAC in tumor tissues. This targeted delivery system ensured that the therapeutic effects were specifically localized to cancer lesions while minimizing off-target effects on healthy cells. Both in vitro and in vivo experiments showcased that only upon NIR photoirradiation could NGP-62 produce singlet oxygen (^1^O_2_), which effectively broke the linkage and released active PROTAC molecules. This light-triggered release mechanism allowed for precise control over BRD4 degradation, a key target involved in tumor growth regulation. This work not only opens up new possibilities for photo-regulation of PROTACs in vivo but also provides valuable insights into designing activatable platforms for targeted protein degradation therapies. The ability to precisely control protein levels within cancer cells offers promising prospects for safely and effectively suppressing tumor growth without causing significant harm to normal tissues.

Recently, He et al*.* reported an innovative approach to achieve controllable target protein degradation using a nano-PROTAC system [142]. They designed a photocaged-PROTAC called phoBET1 and incorporated it into mesoporous silica nanoparticles based on upconversion nanoparticles (UCNPs) to create UMSNs@phoBET1 (NGP-63, (Fig. 17H) nanocages. These nanocages can be activated by NIR light with a wavelength of 980 nm. When exposed to NIR light, the NGP-63 nanocages release active PROTAC in a controlled manner. This active PROTAC specifically targets BRD4, which plays a crucial role in cancer cell growth and survival. By inducing BRD4 degradation, the nano-PROTAC effectively triggers apoptosis in MV-4–11 cancer cells. To evaluate the potential of this novel nanoplatform for clinical applications, in vivo experiments were conducted. The results demonstrated that when NIR light was applied to tumor tissues containing NGP-63 nanocages, BRD4 degradation occurred, leading to significant suppression of tumor growth. This breakthrough technology addresses some limitations associated with existing short-wavelength light-controlled PROTACs. Short-wavelength light often suffers from poor tissue penetration and potential damage to surrounding healthy tissues. In contrast, NIR light has deeper tissue penetration capabilities and is considered safer for biomedical applications. The development of this NIR light activatable PROTAC nanoplatform opens up new possibilities for precise regulation of targeted protein degradation within living tissues. It provides researchers with an advanced tool for studying cellular processes involving specific proteins and offers potential therapeutic strategies for diseases such as cancer where abnormal protein expression plays a critical role.

Ferroptosis, a programmed cell death mechanism, plays a key role in the pathogenesis of numerous diseases [150–152]. The key players in cellular resistance to ferroptosis are glutathione peroxidase 4 (GPX4) and dihydroorotate dehydrogenase (DHODH) [153, 154]. Consequently, targeting the inactivation of these proteins presents an exceptional opportunity for efficacious synergistic cancer therapy based on ferroptosis. Yao et al*.* developed a multifunctional nano-PROTAC called BPNpro (NGP-64, Fig. 17I), which holds immense potential for targeted treatment of tumors [143]. NGP-64 is created using a nanoprecipitation method that involves the use of a thermoresponsive liposome. This unique formulation allows for the encapsulation of a GPX4 targeting boron dipyrromethene (Bodipy) probe (BP) inside the liposome, while on its outer surface, a DHODH ROTAC with cathepsin B (CatB)-cleavable peptide modification (DPCP) is attached. One remarkable feature of NGP-64 is its response to near-infrared (NIR) photoirradiation. When exposed to NIR light, NGP-64 undergoes melting and releases BP specifically within tumor cells. Once released, BP acts as an inhibitor by forming a covalent bond with selenocysteine at the active site of GPX4 enzyme activity. This inhibition disrupts the antioxidant defense mechanism employed by cancer cells and renders them vulnerable to oxidative stress-induced cell death. Furthermore, DPCP plays an essential role in achieving enduring degradation of DHODH through activation by CatB that is overexpressed within tumor cells. By selectively degrading DHODH, DPCP contributes to impairing cancer cell metabolism and inhibiting their proliferation. The combined action of GPX4 inhibition through BP and sustained degradation of DHODH via DPCP leads to extensive ferroptosis—an iron-dependent form of regulated cell death characterized by lipid peroxidation and ROS generation. Ferroptosis induction ultimately results in efficient eradication of tumor cells. Both in vivo and in vitro studies have demonstrated promising outcomes for this novel ferroptosis-based therapy approach proposed by Yao et al*.*. The antitumor effect exhibited by this therapeutic strategy has shown excellent efficacy against various types of cancers tested so far. This breakthrough not only opens up new possibilities for targeted cancer therapies but also highlights the importance and potential applications of nanotechnology in medicine. Further research will undoubtedly focus on optimizing this innovative nano-agent design and exploring its full potential as an effective treatment option for patients battling cancer worldwide.

Therapy-induced DNA damage represents a prevailing approach to impede tumor cell proliferation; however, the therapeutic effectiveness is constrained by the intricate machinery of DNA repair. Carrier-free nano-PROTAC, known as SDNpro (NGP-65, Fig. 17J), has emerged as a groundbreaking approach to enhance the effectiveness of PDT in cancer treatment. Researchers have developed NGP-65 by combining two key components: chlorine e6 (Ce6), a potent photosensitizer with excellent light absorption properties; and dBET57, a specific PROTAC molecule designed to degrade BRD4 protein involved in DNA repair processes [144]. Through self-assembly mediated by noncovalent interactions between Ce6 and dBET57, NGP-65 was formed with favorable dispersibility and uniform nanosize distribution without the need for additional drug excipients. Upon exposure to light irradiation at an appropriate wavelength, NGP-65 efficiently produced abundant ROS within tumor cells. These highly reactive molecules induce significant oxidative damage to cellular components including DNA. Simultaneously, the presence of dBET57 leads to degradation of BRD4 protein through targeted proteolysis. This dual mechanism synergistically enhances the efficacy of PDT by intensifying oxidative DNA damage while interrupting crucial DNA repair pathways mediated by BRD4. The unique advantage offered by NGP-65 lies in its ability to suppress tumor growth while minimizing systemic side effects commonly associated with conventional chemotherapy drugs or carrier-based delivery systems. By specifically targeting BRD4 for degradation within cancer cells during PDT treatment, NGP-65 provides a promising strategy for improving clinical outcomes in tumor therapy. In conclusion, the development of carrier-free nano-PROTACs such as NGP-65 represents an innovative approach towards enhancing PDT efficacy against tumors. With further research and refinement, these advancements hold great potential for advancing PROTAC-based therapies into clinical practice for effective tumor treatment without compromising patient safety or well-being.

Hu et al*.* reported a PROTAC-Cy7, tridentate molecular probe, using a three-in-one molecular design strategy, which incorporated the E3 ligase ligand pomalidomide, a warhead targeting MCL-1, and a heptamethine cyanine linkage connecting the warhead and the ligand within the same scaffold [145]. To overcome water insolubility, bovine serum albumin (BSA) nanoparticles with exceptional biocompatibility, nonantigenicity, and biosafety were employed to encapsulate PROTAC-Cy7. The resulting PROTACCy7@BSA (NGP-66, Fig. 17K) exhibited tumor-targeting capabilities and effectively induced degradation of MCL-1 by specifically binding to overexpressed MCL-1 receptors in various tumors. Furthermore, NGP-66 demonstrated successful utilization in noninvasive biomedical vascular imaging within the NIR-II range and enable real-time intraoperative imaging guidance for tumor resection. When exposed to 808 nm laser irradiation, NGP-66 exhibited a synergistic Chemo-Phototherapy effect, resulting in impeccable tumor suppression and a remarkable 100% survival rate throughout the entire two-month monitoring period. This research presents a straightforward and viable approach for the development of multifunctional agents that enable precise imaging-guided therapy targeting tumors through multiple modalities.

The activation of cell cycle progression in cancer cells renders CDK4/6 inhibition-based cell cycle arrest a potent therapeutic strategy for cancer treatment. To optimize therapeutic efficacy, it is imperative to explore combination strategies based on CDK4/6 inhibition. Wang et al*.* conducted a groundbreaking study that demonstrated the potential of combining CDK4/6 PROTAC with Chlorin e6-based PDT for treating cancer [146]. When these two therapeutic approaches were combined, leading to enhanced anti-cancer activity. The synergistic impact was facilitated through the accumulation and activation of mitochondria, resulting in augmented generation of ROS and induction of apoptosis. To facilitate the translation of their findings into clinical practice, Wang et al*.* developed a self-assembled nano-PROTAC system that could co-deliver CDK4/6 PROTAC and Chlorin e6-based PDT agents without the need for additional carriers. This carrier-free nanoparticle system not only improved drug delivery efficiency but also ensured precise targeting of cancer cells. Remarkably, NGP-67 (Fig. 17L) exhibited even higher levels of apoptosis induction compared to individual treatments alone. This combination therapy had an added benefit—it cooperatively induced ICD and chemotaxis of immune cells. Additionally, NGP-67 remodeled the immunosuppressive tumor microenvironment by enhancing anti-tumor immunity through various mechanisms such as attracting immune cells towards tumors and modulating immunosuppressive factors present in the tumor microenvironment. Overall, this study presents a promising strategy for combining G1 cell cycle blockage with PDT using CDK4/6 PROTACs and Chlorin e6-based agents. The development of carrier-free nanoparticles provides a practical solution for delivering these therapeutics simultaneously while maximizing their efficacy against cancer cells.

PROTACs represent a promising approach for protein degradation via the proteasome pathway, holding significant potential in nanotherapy. However, their efficacy in degrading target proteins remains suboptimal, impeding the achievement of desired outcomes and ultimately leading to therapeutic failure in tumor treatment. Two-dimensional (2D) nanosystem, also known as 2D-nano-PROTAC (NGP-68, Fig. 17M), has been developed using tantalum telluride (TaTe2) nanosheet loaded with PROTAC (ARV-825) [147]. The main objective of constructing NGP-68 is to increase the contact probability between the E3 ligase and target protein, thereby enhancing the protein degradation performance. By efficiently degrading BRD4 and C-MYC proteins, NGP-68 can significantly improve the efficacy of photothermal and PDT for tumors. One remarkable feature of NGP-68 is its ability to serve as a photothermal optical coherence tomography (PT-OCT) contrast agent. This means that it can provide high-resolution three-dimensional images of tumor tissue at a micron level. These detailed images are crucial in guiding multimodal tumor therapy approaches. The construction of therapeutic strategies involving NGP-68 has proven to be highly effective in improving the degradation efficiency of PROTACs towards target proteins. This enhanced efficiency ultimately leads to an improved therapeutic effect when utilizing nano preparations for treating tumors. In summary, by harnessing the unique properties of TaTe2 nanosheet and loading them with PROTAC, researchers have successfully developed NGP-68. These systems not only enhance protein degradation performance but also enable precise imaging through PT-OCT technology. With their potential applications in guided multimodal tumor therapy, these advancements hold great promise for improving cancer treatment outcomes.

Liu et al*.* also introduced a GSH-scavenging nano-PROTAC strategy, which enhances the bioavailability of PROTACs and optimizes their potential to degrade intracellular POIs for tumor therapy [148]. The nano-PROTACs are formulated by encapsulating PROTACs within disulfide amide GSH-responsive poly polymeric (PDSA) nanoparticles, demonstrating that ARV@PDSA nano-PROTAC (NGP-69, Fig. 17N), a nanoengineered BRD4 PROTAC known as ARV-771, effectively promotes BRD4 degradation while reducing downstream oncogene c-Myc expression. By leveraging the GSH-scavenging capability to induce cell cycle arrest and amplify c-Myc-related ferroptosis, NGP-69 demonstrates enhanced anti-tumor efficacy when administered at low doses and exhibits exceptional in vivo biocompatibility. These findings unveil the potential of the nano-PROTAC NGP-69 in treating various diseases through targeted dismantling of associated pathogenic proteins.

## Conclusion

In recent years, heterobifunctional PROTACs have emerged as a highly promising therapeutic modality. These innovative molecules offer fascinating potentials in the field of drug development. One of the most significant benefits offered by PROTACs lies in their exceptional capacity to selectively target and degrade POIs that were previously deemed impervious to pharmacological intervention. This opens up new possibilities for treating diseases caused by these elusive protein targets. Another exciting aspect of PROTACs is their potential to degrade protein targets catalytically. By harnessing this mechanism, PROTACs can effectively combat drug resistance induced by mutations in the targeted proteins. This provides hope for patients who have developed resistance to traditional drugs and are in need of alternative treatment options. Despite these inspiring advances, there are still challenges when it comes to translating PROTACs into clinical applications. One issue is that always-on PROTACs often exhibit on-target but off-tissue effects. This means that while they effectively degrade the intended protein target, they may also cause systemic toxicity by degrading proteins in normal tissues. Additionally, traditional PROTACs face limitations due to their poor water solubility, high polarity, and limited membrane permeability. These properties hinder their oral administration and limit their potential as convenient treatment options for patients. Therefore, extensive efforts have been dedicated to improving the formulation and delivery methods of PROTACs with the aim of enhancing their bioavailability and reducing side effects.

In addition to optimizing the molecular structure of PROTAC, extensive researches have been conducted on small-molecule PROTAC prodrugs in order to achieve precise control over spatiotemporally tunable protein degradation. The development of intracellular assembled PROTACs (click-release PROTACs) has shown promise in overcoming the limitations caused by the large molecular weight of traditional PROTAC molecules. By utilizing two rationally designed precursor ligands, these click-release PROTACs are able to avoid the disadvantages associated with their size. However, it is important to consider the in vitro reaction efficacy and suitable molecular ratio of these precursors owing to the distinct PK profiles exhibited by each unit. In an effort to enhance the functionality of PROTAC molecules, photo-responsive masks have been integrated into their structure, resulting in photo-activatable PROTAC prodrugs. Unfortunately, one major setback in this approach is that UV light lacks sufficient tissue penetration ability, which hinders the development and application of these photo-activatable prodrugs. To address this issue, researchers have explored alternative strategies for activating PROTAC prodrugs within solid tumors. They have leveraged endogenous hallmarks such as hypoxia and elevated levels of enzymes like GSH and ROS to achieve precise protein degradation. While these approaches show promise, there is still a need for additional responsive moieties in prodrug design. This inevitably leads to an increase in molecular weight for PROTACs, which may pose challenges when it comes to their druggability.

Ligand-modified PROTAC prodrugs have emerged as promising therapeutic agents for targeted protein degradation in tumor cells. These prodrugs, such as folate-targeting PROTAC prodrugs and biomacromolecule-PROTAC conjugates, exhibit high tumor specificity due to their ability to selectively bind to specific receptors or biomolecules expressed on the surface of cancer cells. One key advantage of these ligand-modified PROTAC prodrugs is their ability to deliver the degrader cargoes directly to the diseased tissues. By exploiting the target selection property of the ligands, these conjugates can effectively transport the degraders specifically to cancerous sites while minimizing exposure to healthy tissues. This targeted delivery approach holds great potential for overcoming poor cell permeability issues commonly encountered with traditional small-molecule drugs. Furthermore, ligand modification also allows for responsive release features in these PROTAC prodrugs. This means that once internalized by cancer cells, they can efficiently release their active degrader molecules at the desired site of action. This controlled release mechanism not only enhances efficacy but also minimizes off-tissue toxicities by confining exposure solely within the diseased tissue. However, designing effective ligand-modified PROTAC prodrugs requires careful consideration of several factors. Firstly, selecting an appropriate ligand that exhibits high affinity and selectivity towards its target receptor is crucial for achieving optimal therapeutic outcomes. Additionally, ensuring stability in circulation is essential to prevent premature degradation or loss of activity before reaching the intended target site. Moreover, it is important to design ligands that facilitate efficient release of active degraders upon internalization into cancer cells. The timing and extent of this release should be carefully controlled to maximize therapeutic efficacy while minimizing any potential adverse effects.

The development of advanced PROTACs has been a subject of extensive research, with the nano-PROTAC polymers being investigated as potential candidates. These polymers have shown promising results in their ability to enhance the efficacy and reduce toxicity in disease therapeutics. To achieve this, the PROTACs are reversibly conjugated onto the backbone of an amphiphilic copolymer. This unique design allows for self-assembly into nanoparticles, which can then be utilized as nanomedicine. The advantage of using nanomedicine is its ability to selectively accumulate at the tumor site due to its specific targeting properties. Once accumulated at the tumor site, these nanomedicine systems release their integrated PROTACs inside the tumor cells. This targeted delivery ensures that only cancerous cells are affected by POI degradation, minimizing damage to healthy tissues surrounding the tumor. Thus, the nano-PROTAC polymers have made significant advancements in terms of PK, preferential accumulation in diseased tissues, and ultimately enabling a synergistic enhancement of efficacy while reducing toxicity in disease therapeutics. In addition to collaborating with each other, delivery systems can also benefit from the integration of various responsive mechanisms. This integration allows for a more sophisticated and precise control over the release of PROTACs, leading to enhanced therapeutic outcomes. By orchestrating protein degradation with other therapeutic approaches, nanomedicine systems have the potential to optimize treatment efficacy and mitigate adverse effects. However, despite their promising potential, nano-PROTAC polymers face certain challenges that limit their broad application. One such challenge is ensuring the biocompatibility of the selected materials used in these delivery systems. It is crucial to carefully evaluate and select materials that are safe for use in vivo and do not cause any adverse reactions or toxicity. Another important consideration is quality control reliability. As these delivery systems involve complex formulations and processes, it becomes essential to establish robust quality control measures throughout production. This ensures consistency in drug release profiles and maintains product integrity. Moreover, practicality of volume production is another factor that needs attention when considering widespread application of nano-PROTAC polymers. The scalability and cost-effectiveness of manufacturing processes should be evaluated to ensure efficient large-scale production without compromising product quality.

To further facilitate the clinical application of the new-generation of advanced PROTACs (Table 2), it is important to consider various exogenous stimuli that can be used in clinical therapy. For example, ultrasound and radiation are two potential options that could be explored. These methods have been widely used in medical treatments and have proven to be effective in targeting specific areas within the body. In addition to selecting appropriate exogenous stimuli, optimizing the choice of endogenous hallmark of diseased tissue is also crucial for improving disease specificity. This requires researchers to gain a deep understanding of different diseases and their unique characteristics. By doing so, they can identify specific markers or proteins that are present only in diseased tissues and use them as targets for PROTACs. Furthermore, the administration approach of PROTACs plays an important role in their clinical promotion. Patients prefer oral administration over other routes such as injection or infusion due to its convenience and ease-of-use. Therefore, developing smart and precise PROTACs for oral administration should be a top priority for researchers.

**Table 2:** Summary of the various properties of the new-generation advanced PROTACs

Advanced PROTACs	Triggering stimuli	Advantage	Disadvantage	Usage
Click-release PROTAC prodrugs	-	Increased solubility and permeability; targeting effect	Weak activity; PK optimization	Multiple tumors
Folate-targeting PROTAC prodrugs	Hydrolases, etc	Increased solubility and permeability; targeting effect	Higher molecular weight; PK optimization; significant synthetic effort	Multiple tumors
Photo-activatable PROTAC prodrugs	Light	Noninvasive and rapid local activation; targeting effect	Limited light penetration depth; PK optimization; significant synthetic effort	Skin cancer
Radiation-responsive PROTAC prodrugs	Radiation	Deep tissue penetration; targeting effect	Potential DNA damage; PK optimization; significant synthetic effort	Multiple tumors
Enzyme-responsive PROTAC prodrugs	Enzyme	Increased solubility and permeability; targeting effect	PK optimization; significant synthetic effort	Multiple tumors
GSH-responsive PROTAC prodrugs	GSH	Increased solubility and permeability; targeting effect	PK optimization; significant synthetic effort	Multiple tumors
Hypoxia-responsive PROTAC prodrugs	Hypoxia	Increased solubility and permeability; decent in vivo antitumor effect	PK optimization; significant synthetic effort	Multiple tumors
ROS-responsive PROTAC prodrugs	ROS	Increased solubility and permeability; targeting effect	PK optimization; significant synthetic effort	Multiple tumors
Antibody-PROTAC conjugates	GSH, etc	Prolonged circulation; endocytosis-mediated internalization; targeting effect	Potential immunogenicity; optimizing and manufacturing difficulties; restricted loading efficiency	Multiple tumors
Aptamer-PROTAC conjugates	GSH, etc	Endocytosis-mediated internalization; targeting effect	Aptamer diversity; restricted loading efficiency and metabolic stability	NCL overexpre-ssing tumors
Nano-PROTAC polymers	Multiple conditions	Endocytosis-mediated internalization; prolonged circulation; increased solubility; targeting effect	Potential toxicity and immunogenicity caused by carrier materials	Multiple tumors

This review highlights the significant progress made in the development of new-generation advanced PROTACs for cancer therapy. These innovative prodrugs have been engineered to achieve targeted protein degradation while minimizing side effects, by responding to various endogenous or external stimuli such as light, hypoxia, enzyme, X-ray, ROS, and GSH. One of the key advantages of these stimuli-responsive structures is that they allow for incorporation of multifunctional ligands into the PROTAC prodrug. This enables cell selectivity through targeting specific receptors such as antibodies, folate, or aptamers. By combining this with nanomedicine delivery systems, PROTAC prodrugs can further improve PK profiles and enhance tumor tissue accumulation. The potential therapeutic efficacy of these new-generation PROTACs is particularly exciting when combined with other treatment modalities. The synergistic effect achieved through combination therapies has shown great promise in preclinical studies and could lead to more effective treatments for cancer patients. Given the integration of medicinal chemistry, nanomedicine, and materials science, the development of new-generation advanced PROTACs holds great promise for precise protein degradation within tumor cells. This innovative approach has the potential to revolutionize cancer therapy by enabling targeted treatment strategies. In addition to PROTACs, there are several other emerging technologies that offer alternative methods for manipulating protein levels. For example, AUTACs (autophagy-targeting chimeras) and ATTECs (autophagosome-tethering compounds) are designed to target proteins for degradation through the autophagy pathway. These compounds can be used to selectively remove specific proteins from cells, providing researchers with a powerful tool for studying protein function. Another emerging technology is LYTACs (lysosome-targeting chimeras), which are designed to target proteins for degradation in the lysosome. By directing specific proteins to the lysosome for degradation, LYTACs provide researchers with a new approach for controlling protein levels within cells. It is worth noting that all these protein degradation strategies rely on heterobifunctional chimeras similar to PROTACs. While they hold immense potential in advancing precision medicine, it is crucial to acknowledge that challenges may arise during their translation into clinical applications. Issues such as off-target effects or limited efficacy could hinder their successful implementation. Therefore, it becomes imperative to systematically explore and evaluate the new-generation advanced PROTACs alongside other protein degraders. By doing so, we can not only enhance our understanding of these novel approaches but also pave the way for improved therapies across various disease areas beyond cancer. The continuous advancement in this field will undoubtedly contribute towards expanding our toolbox of targeted therapies and ultimately benefit patients worldwide. Through rigorous research and collaboration between scientists from different disciplines, we can unlock the full potential of these innovative techniques and bring about a new era in personalized medicine.

## Data Availability

No datasets were generated or analysed during the current study.

## References

[1] Sakamoto KM, Kim KB, Kumagai A, Mercurio F, Crews CM, Deshaies RJ (2001). Protacs: chimeric molecules that target proteins to the Skp1-Cullin-F box complex for ubiquitination and degradation. Proc Natl Acad Sci USA.

[2] Chirnomas D, Hornberger KR, Crews CW (2023). Protein degraders enter the clinic - a new approach to cancer therapy. Nat Rev Clin Oncol.

[3] Békés M, Langley DR, Crews CM (2022). PROTAC targeted protein degraders: The past is prologue. Nat Rev Drug Discov.

[4] Schneider M, Radoux CJ, Hercules A, Ochoa D, Dunham I, Zalmas LP, Hessler G, Ruf S, Shanmugasundaram V, Hann MM, Thomas PJ, Queisser MA, Benowitz AB, Brown K, Leach AR (2021). The PROTACtable genome. Nat Rev Drug Discov.

[5] Nalawansha DA, Crews CM (2020). PROTACs: an emerging therapeutic modality in precision medicine. Cell Chem Biol.

[6] Li K, Crews CM (2022). PROTACs: Past, present and future. Chem Soc Rev.

[7] Li M, Zhi Y, Liu B, Yao Q (2023). Advancing strategies for proteolysis-targeting chimera design. J Med Chem.

[8] Wang C, Zhang Y, Wu Y, Xing D (2021). Developments of CRBN-based PROTACs as potential therapeutic agents. Eur J Med Chem.

[9] Burslem GM, Crews CM (2020). Proteolysis-targeting chimeras as therapeutics and tools for biological discovery. Cell.

[10] An S, Fu L (2018). Small-molecule PROTACs: An emerging and promising approach for the development of targeted therapy drugs. EBioMedicine.

[11] Wang C, Zhang Y, Wang J, Xing D (2022). VHL-based PROTACs as potential therapeutic agents: Recent progress and perspectives. Eur J Med Chem.

[12] Sun X, Gao H, Yang Y, He M, Wu Y, Song Y, Tong Y, Rao Y (2019). PROTACs: Great opportunities for academia and industry. Signal Transduct Target Ther.

[13] He M, Cao C, Ni Z, Liu Y, Song P, Hao S, He Y, Sun X, Rao Y (2022). PROTACs: Great opportunities for academia and industry (an update from 2020 to 2021). Signal Transduct Target Ther.

[14] Mullard A (2021). Targeted protein degraders crowd into the clinic. Nat Rev Drug Discov.

[15] Garber K (2022). The PROTAC gold rush. Nat Biotechnol.

[16] Wang Y, Jiang X, Feng F, Liu W, Sun H (2020). Degradation of proteins by PROTACs and other strategies. Acta Pharm Sin B.

[17] Kong NR, Jones LH (2023). Clinical translation of targeted protein degraders. Clin Pharmacol Ther.

[18] Bondeson DP, Mares A, Smith IE, Ko E, Campos S, Miah AH, Mulholland KE, Routly N, Buckley DL, Gustafson JL, Zinn N, Grandi P, Shimamura S, Bergamini G, Faelth-Savitski M, Bantscheff M, Cox C, Gordon DA, Willard RR, Flanagan JJ, Casillas LN, Votta BJ, den Besten WI, Famm K, Kruidenier L, Carter PS, Harling JD, Churcher I, Crews CM (2015). Catalytic in vivo protein knockdown by small-molecule PROTACs. Nat Chem Biol..

[19] Churcher I (2018). Protac-induced protein degradation in drug discovery: Breaking the rules or just making new ones?. J Med Chem.

[20] Gu S, Cui D, Chen X, Xiong X, Zhao Y (2018). PROTACs: An emerging targeting technique for protein degradation in drug discovery. BioEssays.

[21] Smith BE, Wang SL, Jaime-Figueroa S, Harbin A, Wang J, Hamman BD, Crews CM (2019). Differential PROTAC substrate specificity dictated by orientation of recruited E3 ligase. Nat Commun.

[22] Lai AC, Crews CM (2017). Induced protein degradation: An emerging drug discovery paradigm. Nat Rev Drug Discov.

[23] Xiong Y, Zhong Y, Yim H, Yang X, Park KS, Xie L, Poulikakos PI, Han X, Xiong Y, Chen X, Liu J, Jin J (2022). Bridged proteolysis targeting chimera (PROTAC) enables degradation of undruggable targets. J Am Chem Soc.

[24] Moreau K, Coen M, Zhang AX, Pachl F, Castaldi MP, Dahl G, Boyd H, Scott C, Newham P (2020). Proteolysis-targeting chimeras in drug development: A safety perspective. Br J Pharmacol.

[25] Wang C, Zhang Y, Xing D, Zhang R (2021). PROTACs technology for targeting non-oncoproteins: Advances and perspectives. Bioorg Chem.

[26] Koroleva OA, Dutikova YV, Trubnikov AV, Zenov FA, Manasova EV, Shtil AA, Kurkin AV (2022). PROTAC: Targeted drug strategy. Principles and limitations. Russ Chem Bull.

[27] Gao J, Yang L, Lei S, Zhou F, Nie H, Peng B, Xu T, Chen X, Yang X, Sheng C, Rao Y, Pu K, Jin J, Xu Z, Yu H (2023). Stimuli-activatable PROTACs for precise protein degradation and cancer therapy. Sci Bull (Beijing).

[28] Chen C, Yang Y, Wang Z, Li H, Dong C, Zhang X (2023). Recent advances in pro-PROTAC development to address on-target off-tumor toxicity. J Med Chem.

[29] Raina K, Lu J, Qian Y, Altieri M, Gordon D, Rossi AM, Wang J, Chen X, Dong H, Siu K, Winkler JD, Crew AP, Crews CM, Coleman KG (2016). PROTAC-induced BET protein degradation as a therapy for castration-resistant prostate cancer. Proc Natl Acad Sci USA.

[30] Edmondson SD, Yang B, Fallan C (2019). Proteolysis targeting chimeras (PROTACs) in 'beyond rule-of-five' chemical space: Recent progress and future challenges. Bioorg Med Chem Lett.

[31] Matsson P, Kihlberg J (2017). How big is too big for cell permeability?. J Med Chem.

[32] Kiely-Collins H, Winter GE, Bernardes GJL (2021). The role of reversible and irreversible covalent chemistry in targeted protein degradation. Cell Chem Biol.

[33] Pike A, Williamson B, Harlfinger S, Martin S, McGinnity DF (2020). Optimising proteolysis-targeting chimeras (PROTACs) for oral drug delivery: A drug metabolism and pharmacokinetics perspective. Drug Discov Today.

[34] Chen Y, Tandon I, Heelan W, Wang Y, Tang W, Hu Q (2022). Proteolysis-targeting chimera (PROTAC) delivery system: Advancing protein degraders towards clinical translation. Chem Soc Rev.

[35] Zeng S, Huang W, Zheng X, Liyan C, Zhang Z, Wang J, Shen Z (2021). Proteolysis targeting chimera (PROTAC) in drug discovery paradigm: Recent progress and future challenges. Eur J Med Chem.

[36] Guenette RG, Yang SW, Min J, Pei B, Potts PR (2022). Target and tissue selectivity of PROTAC degraders. Chem Soc Rev.

[37] Guedeney N, Cornu M, Schwalen F, Kieffer C, Voisin-Chiret AS (2023). PROTAC technology: A new drug design for chemical biology with many challenges in drug discovery. Drug Discov Today.

[38] Liu J, Peng Y, Wei W (2021). Light-controllable PROTACs for temporospatial control of protein degradation. Front Cell Dev Biol.

[39] Dragovich PS (2022). Degrader-antibody conjugates. Chem Soc Rev.

[40] Salerno A, Seghetti F, Caciolla J, Uliassi E, Testi E, Guardigni M, Roberti M, Milelli A, Bolognesi ML (2022). Enriching proteolysis targeting chimeras with a second modality: When two are better than one. J Med Chem.

[41] Benowitz AB, Jones KL, Harling JD (2021). The therapeutic potential of PROTACs. Expert Opin Ther Pat.

[42] Paiva SL, Crews CM (2019). Targeted protein degradation: elements of PROTAC design. Curr Opin Chem Biol.

[43] Li X, Song Y (2020). Proteolysis-targeting chimera (PROTAC) for targeted protein degradation and cancer therapy. J Hematol Oncol.

[44] Zou Y, Ma D, Wang Y (2019). The PROTAC technology in drug development. Cell Biochem Funct.

[45] Alabi SB, Crews CM (2021). Major advances in targeted protein degradation: PROTACs, LYTACs, and MADTACs. J Biol Chem.

[46] Lipinski CA (2004). Lead- and drug-like compounds: The rule-of-five revolution. Drug Discov Today Technol.

[47] Raina K, Crews CM (2017). Targeted protein knockdown using small molecule degraders. Curr Opin Chem Biol.

[48] Deshaies RJ (2015). Protein degradation: Prime time for PROTACs. Nat Chem Biol.

[49] Lebraud H, Wright DJ, Johnson CN, Heightman TD (2016). Protein degradation by in-cell self-assembly of proteolysis targeting chimeras. ACS Cent Sci.

[50] Cyrus K, Wehenkel M, Choi EY, Han HJ, Lee H, Swanson H, Kim KB (2011). Impact of linker length on the activity of PROTACs. Mol Biosyst.

[51] Huang J, Yao Z, Li B, Ping Y (2023). Targeted delivery of PROTAC-based prodrug activated by bond-cleavage bioorthogonal chemistry for microneedle-assisted cancer therapy. J Control Release.

[52] Chang M, Gao F, Pontigon D, Gnawali G, Xu H, Wang W (2023). Bioorthogonal PROTAC prodrugs enabled by on-target activation. J Am Chem Soc.

[53] Bi T, Liang P, Zhou Y, Wang H, Huang R, Sun Q, Shen H, Yang S, Ren W, Liu Z (2023). Rational design of bioorthogonally activatable PROTAC for tumor-targeted protein degradation. J Med Chem.

[54] Ruff E, Poh S (2023). Folate targeting peptide conjugates for inflammatory response suppression. Curr Drug Metab.

[55] George S, Srinivasan A, Tulimilli SV, Madhunapantula SV, Palantavida S (2023). Folate targeting self-limiting hyperthermic nanoparticles for controlled photothermal therapy. J Mater Chem B.

[56] Scaranti M, Cojocaru E, Banerjee S, Banerji U (2020). Exploiting the folate receptor α in oncology. Nat Rev Clin Oncol.

[57] Liu J, Chen H, Liu Y, Shen Y, Meng F, Kaniskan H, Jin J, Wei W (2021). Cancer selective target degradation by folate-caged PROTACs. J Am Chem Soc.

[58] Chen H, Liu J, Kaniskan H, Wei W, Jin J (2021). Folate-guided protein degradation by immunomodulatory imide drug-based molecular glues and proteolysis targeting chimeras. J Med Chem.

[59] Dolmans DE, Fukumura D, Jain RK (2003). Photodynamic therapy for cancer. Nat Rev Cancer.

[60] Tao Y, Chan HF, Shi B, Li M, Leong KW (2020). Light: A magical tool for controlled drug delivery. Adv Funct Mater.

[61] Verma S, Manna D (2020). Controlling PROTACs with light. ChemMedChem.

[62] Hu Z, Crews CM (2022). Recent developments in PROTAC-mediated protein degradation: From bench to clinic. ChemBioChem.

[63] Wu P, Manna D (2020). Optochemical control of protein degradation. ChemBioChem.

[64] Xue G, Wang K, Zhou D, Zhong H, Pan Z (2019). Light-induced protein degradation with photocaged PROTACs. J Am Chem Soc.

[65] Naro Y, Darrah K, Deiters A (2020). Optical control of small molecule-induced protein degradation. J Am Chem Soc.

[66] Liu J, Chen H, Ma L, He Z, Wang D, Liu Y, Lin Q, Zhang T, Gray N, Kaniskan H, Jin J, Wei W (2020). Light-induced control of protein destruction by opto-PROTAC. Sci. Adv..

[67] Kounde CS, Shchepinova MM, Saunders CN, Muelbaier M, Rackham MD, Harling JD, Tate EW (2020). A caged E3 ligase ligand for PROTAC-mediated protein degradation with light. Chem Commun (Camb).

[68] Pfaff P, Samarasinghe KTG, Crews CM, Carreira EM (2019). Reversible spatiotemporal control of induced protein degradation by bistable photoPROTACs. ACS Cent Sci.

[69] Reynders M, Matsuura BS, Bérouti M, Simoneschi D, Marzio A, Pagano M, Trauner D (2020). PHOTACs enable optical control of protein degradation. Sci Adv..

[70] Jin YH, Lu MC, Wang Y, Shan WX, Wang XY, You QD, Jiang ZY (2020). Azo-PROTAC: Novel light-controlled small-molecule tool for protein knockdown. J Med Chem.

[71] Zhang Q, Kounde CS, Mondal M, Greenfield JL, Baker JR, Kotelnikov S, Ignatov M, Tinworth CP, Zhang L, Conole D, De Vita E, Kozakov D, McCluskey A, Harling JD, Fuchter MJ, Tate EW (2022). Light-mediated multi-target protein degradation using arylazopyrazole photoswitchable PROTACs (AP-PROTACs). Chem Commun (Camb).

[72] Vozenin MC, Bourhis J, Durante M (2022). Towards clinical translation of FLASH radiotherapy. Nat Rev Clin Oncol.

[73] Allen C, Her S, Jaffray DA (2017). Radiotherapy for cancer: Present and future. Adv Drug Deliv Rev.

[74] Gong L, Zhang Y, Liu C, Zhang M, Han S (2021). Application of radiosensitizers in cancer radiotherapy. Int J Nanomedicine.

[75] Petroni G, Cantley LC, Santambrogio L, Formenti SC, Galluzzi L (2022). Radiotherapy as a tool to elicit clinically actionable signalling pathways in cancer. Nat Rev Clin Oncol.

[76] Yang C, Yang Y, Li Y, Ni Q, Li J (2023). Radiotherapy-triggered proteolysis targeting chimera prodrug activation in tumors. J Am Chem Soc.

[77] An K, Deng X, Chi H, Zhang Y, Li Y, Cheng M, Ni Z, Yang Z, Wang C, Chen J, Bai J, Ran C, Wei Y, Li J, Zhang P, Xu F, Tan W (2023). Stimuli-responsive protacs for controlled protein degradation. Angew Chem Int Ed Engl.

[78] Chang J, Cai W, Liang C, Tang Q, Chen X, Jiang Y, Mao L, Wang M (2019). Enzyme-instructed activation of pro-protein therapeutics in vivo. J Am Chem Soc.

[79] Fouladi F, Steffen KJ, Mallik S (2017). Enzyme-responsive liposomes for the delivery of anticancer drugs. Bioconjug Chem.

[80] Fejerskov B, Jarlstad Olesen MT, Zelikin AN (2017). Substrate mediated enzyme prodrug therapy. Adv Drug Deliv Rev..

[81] Liang C, Zheng Q, Luo T, Cai W, Mao L, Wang M (2022). Enzyme-catalyzed activation of pro-PROTAC for cell-selective protein degradation. CCS Chemistry.

[82] Wei M, Zhao R, Cao Y, Wei Y, Li M, Dong Z, Liu Y, Ruan H, Li Y, Cao S, Tang Z, Zhou Y, Song W, Wang Y, Wang J, Yang G, Yang C (2021). First orally bioavailable prodrug of proteolysis targeting chimera (PROTAC) degrades cyclin-dependent kinases 2/4/6 in vivo. Eur J Med Chem.

[83] Parkinson EI, Hergenrother PJ (2015). Deoxynyboquinones as NQO1-activated cancer therapeutics. Acc Chem Res.

[84] Bey EA, Reinicke KE, Srougi MC, Varnes M, Anderson VE, Pink JJ, Li LS, Patel M, Cao L, Moore Z, Rommel A, Boatman M, Lewis C, Euhus DM, Bornmann WG, Buchsbaum DJ, Spitz DR, Gao J, Boothman DA (2013). Catalase abrogates β-lapachone-induced PARP1 hyperactivation-directed programmed necrosis in NQO1-positive breast cancers. Mol Cancer Ther.

[85] Kapalatiya H, Madav Y, Tambe VS, Wairkar S (2022). Enzyme-responsive smart nanocarriers for targeted chemotherapy: An overview. Drug Deliv Transl Res.

[86] Hsu PH, Almutairi A (2021). Recent progress of redox-responsive polymeric nanomaterials for controlled release. J Mater Chem B.

[87] Yang Y, Sun W (2022). Recent advances in redox-responsive nanoparticles for combined cancer therapy. Nanoscale Adv.

[88] Chen M, Liu D, Liu F, Wu Y, Peng X, Song F (2021). Recent advances of redox-responsive nanoplatforms for tumor theranostics. J Control Release.

[89] Liu P, Hao L, Liu M, Hu S (2023). Glutathione-responsive and -exhausting metal nanomedicines for robust synergistic cancer therapy. Front Bioeng Biotechnol.

[90] Cheng R, Feng F, Meng F, Deng C, Feijen J, Zhong Z (2011). Glutathione-responsive nano-vehicles as a promising platform for targeted intracellular drug and gene delivery. J Control Release.

[91] Liu C, Jia S, Tu L, Yang P, Wang Y, Ke S, Shi W, Ye S (2022). GSH-responsive and hypoxia-activated multifunctional nanoparticles for synergetically enhanced tumor therapy. ACS Biomater Sci Eng.

[92] Ma Z, Gao X, Raza F, Zafar H, Huang G, Yang Y, Shi F, Wang D, He X (2022). Design of GSH-responsive curcumin nanomicelles for oesophageal cancer therapy. Pharmaceutics.

[93] Zhou Z, Fan H, Yu D, Shi F, Li Q, Zhang Z, Wang X, Zhang X, Dong C, Sun H, Mi W (2023). Glutathione-responsive PROTAC for targeted degradation of ERα in breast cancer cells. Bioorg Med Chem.

[94] Li Y, Zhao L, Li XF (2021). Targeting hypoxia: Hypoxia-activated prodrugs in cancer therapy. Front Oncol.

[95] Wilson WR, Hay MP (2011). Targeting hypoxia in cancer therapy. Nat Rev Cancer.

[96] Borad MJ, Reddy SG, Bahary N, Uronis HE, Sigal D, Cohn AL, Schelman WR, Stephenson J, Chiorean EG, Rosen PJ, Ulrich B, Dragovich T, Del Prete SA, Rarick M, Eng C, Kroll S, Ryan DP (2015). Randomized phase II trial of gemcitabine plus TH-302 versus gemcitabine in patients with advanced pancreatic cancer. J Clin Oncol.

[97] Cheng W, Li S, Wen X, Han S, Wang S, Wei H, Song Z, Wang Y, Tian X, Zhang X (2021). Development of hypoxia-activated PROTAC exerting a more potent effect in tumor hypoxia than in normoxia. Chem Commun (Camb).

[98] Shi S, Du Y, Zou Y, Niu J, Cai Z, Wang X, Qiu F, Ding Y, Yang G, Wu Y, Xu Y, Zhu Q (2022). Rational design for nitroreductase (NTR)-responsive proteolysis targeting chimeras (PROTACs) selectively targeting tumor tissues. J Med Chem.

[99] Do TC, Lau JW, Sun C, Liu S, Kha KT, Lim ST, Oon YY, Kwan YP, Ma JJ, Mu Y, Liu X, Carney TJ, Wang X, Xing B (2022). Hypoxia deactivates epigenetic feedbacks via enzyme-derived clicking proteolysis-targeting chimeras. Sci. Adv..

[100] Cheng W, Li S, Han S, Miao R, Wang S, Liu C, Wei H, Tian X, Zhang X (2023). Design, synthesis and biological evaluation of the tumor hypoxia-activated PROTACs bearing caged CRBN E3 ligase ligands. Bioorg Med Chem.

[101] Xie B, Xu B, Xin L, Wei Y, Guo X, Dong C (2023). Discovery of estrogen receptor α targeting caged hypoxia-responsive PROTACs with an inherent bicyclic skeleton for breast cancer treatment. Bioorg Chem.

[102] Yu H, Jin F, Liu D, Shu G, Wang X, Qi J, Sun M, Yang P, Jiang S, Ying X, Du Y (2020). ROS-responsive nano-drug delivery system combining mitochondria-targeting ceria nanoparticles with atorvastatin for acute kidney injury. Theranostics.

[103] Tao W, He Z (2018). ROS-responsive drug delivery systems for biomedical applications. Asian J Pharm Sci.

[104] Barnham KJ, Masters CL, Bush AI (2004). Neurodegenerative diseases and oxidative stress. Nat Rev Drug Discov.

[105] Liu H, Ren C, Sun R, Wang H, Zhan Y, Yang X, Jiang B, Chen H (2022). Reactive oxygen species-responsive pre-PROTAC for tumor-specific protein degradation. Chem Commun (Camb).

[106] Yu D, Fan H, Zhou Z, Zhang Y, Sun J, Wang L, Jia Y, Tian J, Campbell A, Mi W, Sun H (2023). Hydrogen peroxide-inducible PROTACs for targeted protein degradation in cancer cells. ChemBioChem.

[107] Teicher BA, Morris J (2022). Antibody-drug conjugate targets, drugs, and linkers. Curr Cancer Drug Targets.

[108] Fu Z, Li S, Han S, Shi C, Zhang Y (2022). Antibody drug conjugate: The "biological missile" for targeted cancer therapy. Signal Transduct Target Ther.

[109] Jin Y, Schladetsch MA, Huang X, Balunas MJ, Wiemer AJ (2022). Stepping forward in antibody-drug conjugate development. Pharmacol Ther.

[110] Iwata TN, Ishii C, Ishida S, Ogitani Y, Wada T, Agatsuma T (2018). A HER2-targeting antibody-drug conjugate, trastuzumab deruxtecan (DS-8201a), enhances antitumor immunity in a mouse model. Mol Cancer Ther.

[111] Ogitani Y, Hagihara K, Oitate M, Naito H, Agatsuma T (2016). Bystander killing effect of DS-8201a, a novel anti-human epidermal growth factor receptor 2 antibody-drug conjugate, in tumors with human epidermal growth factor receptor 2 heterogeneity. Cancer Sci.

[112] Pillow TH, Adhikari P, Blake RA, Chen J, Del Rosario G, Deshmukh G, Figueroa I, Gascoigne KE, Kamath AV, Kaufman S, Kleinheinz T, Kozak KR, Latifi B, Leipold DD, Sing-Li C, Li R, Mulvihill MM, O'Donohue A, Rowntree RK, Sadowsky JD, Wai J, Wang X, Wu C, Xu Z, Yao H, Yu SF, Zhang D, Zang R, Zhang H, Zhou H, Zhu X, Dragovich PS (2020). Antibody conjugation of a chimeric bet degrader enables in vivo activity. Chem Med Chem.

[113] Maneiro MA, Forte N, Shchepinova MM, Kounde CS, Chudasama V, Baker JR, Tate EW (2020). Antibody-PROTAC conjugates enable HER2-dependent targeted protein degradation of BRD4. ACS Chem Biol.

[114] Dragovich PS, Pillow TH, Blake RA, Sadowsky JD, Adaligil E, Adhikari P, Bhakta S, Blaquiere N, Chen J, Dela Cruz-Chuh J, Gascoigne KE, Hartman SJ, He M, Kaufman S, Kleinheinz T, Kozak KR, Liu L, Liu L, Liu Q, Lu Y, Meng F, Mulvihill MM, O'Donohue A, Rowntree RK, Staben LR, Staben ST, Wai J, Wang J, Wei B, Wilson C, Xin J, Xu Z, Yao H, Zhang D, Zhang H, Zhou H, Zhu X (2021). Antibody-mediated delivery of chimeric BRD4 degraders. Part 1: Exploration of antibody linker, payload loading, and payload molecular properties. J Med Chem..

[115] Dragovich PS, Pillow TH, Blake RA, Sadowsky JD, Adaligil E, Adhikari P, Chen J, Corr N, Dela Cruz-Chuh J, Del Rosario G, Fullerton A, Hartman SJ, Jiang F, Kaufman S, Kleinheinz T, Kozak KR, Liu L, Lu Y, Mulvihill MM, Murray JM, O'Donohue A, Rowntree RK, Sawyer WS, Staben LR, Wai J, Wang J, Wei B, Wei W, Xu Z, Yao H, Yu SF, Zhang D, Zhang H, Zhang S, Zhao Y, Zhou H, Zhu X (2021). Antibody-mediated delivery of chimeric BRD4 degraders Part 2: Improvement of in vitro antiproliferation activity and in vivo antitumor efficacy. J Med Chem..

[116] Vartak R, Deore B, Sanhueza CA, Patel K (2023). Cetuximab-based PROteolysis targeting chimera for effectual downregulation of NSCLC with varied EGFR mutations. Int J Biol Macromol.

[117] Dragovich PS, Adhikari P, Blake RA, Blaquiere N, Chen J, Cheng YX, Den Besten W, Han J, Hartman SJ, He J, He M, Rei Ingalla E, Kamath AV, Kleinheinz T, Lai T, Leipold DD, Li CS, Liu Q, Lu J, Lu Y, Meng F, Meng L, Ng C, Peng K, Lewis-Phillips G, Pillow TH, Rowntree RK, Sadowsky JD, Sampath D, Staben L, Staben ST, Wai J, Wan K, Wang X, Wei B, Wertz IE, Xin J, Xu K, Yao H, Zang R, Zhang D, Zhou H, Zhao Y (2020). Antibody-mediated delivery of chimeric protein degraders which target estrogen receptor alpha (ERα). Bioorg Med Chem Lett..

[118] Chan K, Sathyamurthi PS, Queisser MA, Mullin M, Shrives H, Coe DM, Burley GA (2023). Antibody-proteolysis targeting chimera conjugate enables selective degradation of receptor-interacting serine/threonine-protein kinase 2 in HER2+ cell lines. Bioconjug Chem.

[119] Sang X, Zhang Y, Fang F, Gao L, Tao Y, Li X, Zhang Z, Wang J, Tian Y, Li Z, Yao D, Wu Y, Chu X, Zhang K, Ma L, Lu L, Chen Y, Yu J, Zhuo R, Wu S, Zhang Z, Pan J, Hu S (2022). BRD4 inhibitor GNE-987 exerts anticancer effects by targeting super-enhancer-related gene LYL1 in acute myeloid leukemia. J Immunol Res.

[120] Nimjee SM, White RR, Becker RC, Sullenger BA (2017). Aptamers as therapeutics. Annu Rev Pharmacol Toxicol.

[121] Bates PJ, Laber DA, Miller DM, Thomas SD, Trent JO (2009). Discovery and development of the G-rich oligonucleotide AS1411 as a novel treatment for cancer. Exp Mol Pathol.

[122] Zhu G, Niu G, Chen X (2015). Aptamer-drug conjugates. Bioconjug Chem.

[123] He S, Gao F, Ma J, Ma H, Dong G, Sheng C (2021). Aptamer-PROTAC conjugates (APCs) for tumor-specific targeting in breast cancer. Angew Chem Int Ed Engl.

[124] Zhang L, Li L, Wang X, Liu H, Zhang Y, Xie T, Zhang H, Li X, Peng T, Sun X, Dai J, Liu J, Wu W, Ye M, Tan W (2022). Development of a novel PROTAC using the nucleic acid aptamer as a targeting ligand for tumor selective degradation of nucleolin. Mol Ther Nucleic Acids.

[125] Chen M, Zhou P, Kong Y, Li J, Li Y, Zhang Y, Ran J, Zhou J, Chen Y, Xie S (2023). Inducible degradation of oncogenic nucleolin using an aptamer-based PROTAC. J Med Chem.

[126] Shih PC, Naganuma M, Tsuji G, Demizu Y, Naito M (2023). Development of decoy oligonucleotide-warheaded chimeric molecules targeting STAT3. Bioorg Med Chem.

[127] Yang K, Yang Z, Yu G, Nie Z, Wang R, Chen X (2022). Polyprodrug nanomedicines: An emerging paradigm for cancer therapy. Adv Mater.

[128] Li Z, Xiao C, Yong T, Li Z, Gan L, Yang X (2020). Influence of nanomedicine mechanical properties on tumor targeting delivery. Chem Soc Rev.

[129] Jain RK, Stylianopoulos T (2010). Delivering nanomedicine to solid tumors. Nat Rev Clin Oncol.

[130] Liang C, Xu L, Song G, Liu Z (2016). Emerging nanomedicine approaches fighting tumor metastasis: Animal models, metastasis-targeted drug delivery, phototherapy, and immunotherapy. Chem Soc Rev.

[131] Yan WL, Lang TQ, Yuan WH, Yin Q, Li YP (2022). Nanosized drug delivery systems modulate the immunosuppressive microenvironment to improve cancer immunotherapy. Acta Pharmacol Sin.

[132] Lv L, Shi Y, Wu J, Li G (2021). Nanosized drug delivery systems for breast cancer stem cell targeting. Int J Nanomedicine.

[133] Gao J, Wang WQ, Pei Q, Lord MS, Yu HJ (2020). Engineering nanomedicines through boosting immunogenic cell death for improved cancer immunotherapy. Acta Pharmacol Sin.

[134] Moon Y, Jeon SI, Shim MK, Kim K (2023). Cancer-specific delivery of proteolysis-targeting chimeras (PROTACs) and their application to cancer immunotherapy. Pharmaceutics.

[135] Gao J, Hou B, Zhu Q, Yang L, Jiang X, Zou Z, Li X, Xu T, Zheng M, Chen YH, Xu Z, Xu H, Yu H (2022). Engineered bioorthogonal POLY-PROTAC nanoparticles for tumour-specific protein degradation and precise cancer therapy. Nat Commun.

[136] Xie Z, Fan T, An J, Choi W, Duo Y, Ge Y, Zhang B, Nie G, Xie N, Zheng T, Chen Y, Zhang H, Kim JS (2020). Emerging combination strategies with phototherapy in cancer nanomedicine. Chem Soc Rev.

[137] Wu D, Yang K, Zhang Z, Feng Y, Rao L, Chen X, Yu G (2022). Metal-free bioorthogonal click chemistry in cancer theranostics. Chem Soc Rev.

[138] Zhang C, Zeng Z, Cui D, He S, Jiang Y, Li J, Huang J, Pu K (2021). Semiconducting polymer nano-PROTACs for activatable photo-immunometabolic cancer therapy. Nat Commun.

[139] Zhang C, He S, Zeng Z, Cheng P, Pu K (2022). Smart Nano-PROTACs reprogram tumor microenvironment for activatable photo-metabolic cancer immunotherapy. Angew Chem Int Ed Engl.

[140] Zhang C, Xu M, He S, Huang J, Xu C, Pu K (2023). Checkpoint nano-PROTACs for activatable cancer photo-immunotherapy. Adv Mater.

[141] Wang W, Zhu C, Zhang B, Feng Y, Zhang Y, Li J (2023). Self-assembled nano-PROTAC enables near-infrared photodynamic proteolysis for cancer therapy. J Am Chem Soc.

[142] He Q, Zhou L, Yu D, Zhu R, Chen Y, Song M, Liu X, Liao Y, Ding T, Fan W, Yu W (2023). Near-infrared-activatable PROTAC nanocages for controllable target protein degradation and on-demand antitumor therapy. J Med Chem.

[143] Yao L, Yang N, Zhou W, Akhtar MH, Zhou W, Liu C, Song S, Li Y, Han W, Yu C (2023). Exploiting cancer vulnerabilities by blocking of the DHODH and GPX4 pathways: A multifunctional bodipy/PROTAC Nanoplatform for the efficient synergistic ferroptosis therapy. Adv Healthc Mater.

[144] Zhao LP, Rao XN, Zheng RR, Huang CY, Kong RJ, Cheng H, Li B, Li SY (2023). Carrier-free nano-PROTACs to amplify photodynamic therapy induced DNA damage through BRD4 degradation. Nano Lett.

[145] Hu Z, Li R, Cui X, Hu C, Chen Z (2023). Tailoring albumin-based theranostic PROTACs nanoparticles for enhanced NIR-II bioimaging and synergistic cancer chemo-phototherapy. Chem Eng J.

[146] Wang T, Zhang Y, Chen K, Huang Y, Liu Y, Xu S, Wang W (2023). CDK4/6 nano-PROTAC enhances mitochondria-dependent photodynamic therapy and anti-tumor immunity. Nano Today.

[147] Liu H, Chen C, Chen H, Mo L, Guo Z, Ye B, Liu Z (2022). 2D-PROTACs with augmented protein degradation for super-resolution photothermal optical coherence tomography guided momentary multimodal therapy. Chem Eng J.

[148] Liu HJ, Chen W, Wu G, Zhou J, Liu C, Tang Z, Huang X, Gao J, Xiao Y, Kong N, Joshi N, Cao Y, Abdi R, Tao W (2023). Glutathione-scavenging nanoparticle-mediated PROTACs delivery for targeted protein degradation and amplified antitumor effects. Adv Sci (Weinh).

[149] Olson OC, Joyce JA (2015). Cysteine cathepsin proteases: Regulators of cancer progression and therapeutic response. Nat Rev Cancer.

[150] Lei G, Mao C, Yan Y, Zhuang L, Gan B (2021). Ferroptosis, radiotherapy, and combination therapeutic strategies. Protein Cell.

[151] Xu H, Ye D, Ren M, Zhang H, Bi F (2021). Ferroptosis in the tumor microenvironment: Perspectives for immunotherapy. Trends Mol Med.

[152] Yang F, Xiao Y, Ding JH, Jin X, Ma D, Li DQ, Shi JX, Huang W, Wang YP, Jiang YZ, Shao ZM (2023). Ferroptosis heterogeneity in triple-negative breast cancer reveals an innovative immunotherapy combination strategy. Cell Metab.

[153] Mao C, Liu X, Zhang Y, Lei G, Yan Y, Lee H, Koppula P, Wu S, Zhuang L, Fang B, Poyurovsky MV, Olszewski K, Gan B (2021). DHODH-mediated ferroptosis defence is a targetable vulnerability in cancer. Nature.

[154] Xu L, Liu Y, Chen X, Zhong H, Wang Y (2023). Ferroptosis in life: To be or not to be. Biomed Pharmacother.

